# Factors affecting the infestation status of African sugarcane stalk borer (*Eldana saccharina* Walker) (Lepidoptera: Pyralidae) in Tanzania

**DOI:** 10.1371/journal.pone.0355844

**Published:** 2026-08-31

**Authors:** Hamis Daniel Wambura, Gration Mutashoberwa Rwegasira, Martin John Martin

**Affiliations:** 1 Department of Crop Science and Horticulture, Sokoine University of Agriculture, Morogoro, Tanzania; 2 Mwalimu Nyerere University of Agriculture and Technology, Butiama, Tanzania; 3 Institute of Pest Management, Sokoine University of Agriculture, Morogoro, Tanzania; Veracruzana University: Universidad Veracruzana, MEXICO

## Abstract

Sugarcane is an important crop that contributes substantially to sugar production globally and across Sub-Saharan Africa. However, its productivity is constrained by stem borer infestations, particularly *Eldana saccharina.* The pest reduces sugarcane yield by up to 30% and about 18% of sucrose content is compromised. Nonetheless, the influence of ecological parameters on its pest status in Tanzania remains poorly understood. This study evaluated the influence of season, altitude, crop age, and sugarcane variety on *E. saccharina* infestation in Tanzania. A stratified multistage cross-sectional survey was conducted across three altitudes representing low, medium, and high altitudes. In total, 711 blocks were surveyed, and infestation was assessed from 50 randomly sampled stalks per block. The infestation was quantified as the total number of *E. saccharina* larvae recovered per 50 stalks alongside other damage parameters. Data were analyzed using generalized linear mixed models in R, with likelihood ratio tests and ${Type\;III\;Wald\;x}^{\text{2}}$ tests. Infestation was highest at high altitude in both seasons and was significantly influenced by season x altitude x crop-age interaction ${(\;X}^{\text{2}}=\text{9}.\text{5}\text{8},\text{ P}<\text{0}.\text{0}\text{0}\text{8}\text{3}$). Internal stalk-tissue- damage was significantly greater at high-altitude during the rainy season (P = 0.04). Boring intensity was significantly higher at medium and high-altitudes during the rainy season (P =0.013;P = 0.004, respectively), with the bottom stalk part most affected (P = 0.001). Higher infestation was observed in mixed-variety blocks and in cultivars N41 and N49. These findings show that altitude, season, and crop-age are determinants of *E. saccharina* infestation in Tanzania and should guide pest management strategies.

## 1. Introduction

Sugarcane is a perennial, high-value commercial crop cultivated globally to meet increasing sugar demand [1,2]. Current production exceeds two billion metric tons annually, with Brazil leading global output at approximately 798 million metric tons, followed by India at 421.15 million metric tons [3]. In Sub-Saharan Africa, South Africa is the dominant producer, contributing about 17.94 million metric tons, while in East Africa, Uganda leads regional production with approximately 6.19 million metric tons [2]. In Tanzania, sugarcane is an important commercial crop, contributing substantially to domestic sugar production and rural livelihoods [4]. National survey data show that the crop is cultivated on approximately 53,398 ha, indicating its wide production base in the country [4]. More recent reports indicate that the sugar industry produced 431,736.74 tons of sugar in the 2024/25 season [5]. In addition to its contribution to national sugar supply, the sector is economically significant because it generates employment, with about 28,500 direct and 95,000 indirect employment opportunities estimated across the sugarcane value chain [5,6]. Despite its economic significance, sugarcane productivity is constrained by multiple biotic and abiotic factors [7,8]. Abiotic factors such as drought, extreme temperature and soil nutrient limitations affect sugarcane growth and sucrose accumulation [9]. Biotic factors, particularly insect pests, pose substantial threats to sugarcane yields. Among these, stem borers are recognized as the most destructive pests in sugarcane production. Key species of global importance include *Eldana saccharina* in Africa*, Diatraea saccharalis* in the Americas*, Chilo sacchariphagus* in Asia and the Indian Ocean region, and *Scirpophaga exerptalis* in South Asia [10,11]. In Sub-Saharan Africa, *E. saccharina* is regarded as the most economically important stem borer because it causes substantial losses in cane yield and sucrose content [9]. Like other stem borers, *E. saccharina* is also difficult to manage effectively, as its larvae develop internally within sugarcane stalk tissues, where they are protected from contact insecticides and less accessible to natural enemies, thus, reducing the effectiveness of conversional control measures [12].

The pest was first recorded in West Africa more than a century ago and subsequently spread across major sugarcane-producing regions including Mozambique, South Africa, Zimbabwe, Benin, Kenya, Uganda and Tanzania [13]. In South Africa, *E. saccharina* became established in the early 1900s and was formally recognized as a serious economic pest by 1939 after documented yield reductions up to 30% and sucrose losses approaching 18% [14]. Similar economic importance was later reported in East Africa, where the pest was recorded in Tanzania in the early twentieth century before spreading regionally [15].

The distribution of *E. saccharina* has not remained static. Historically, confined largely to coastal and lowland regions in countries like South Africa and Ethiopia [16–18], the pest has progressively expanded into inland and higher altitude sugarcane-producing zones [19]. Such shifts have been associated with environmental and climatic changes [20,21]. Seasonal rainfall patterns, altitude-related temperatures, crop age and sugarcane variety susceptibility have been reported to influence infestation level, larval survivability, and the extent of internal stalk damage [14,22–24]. For instance, increased infestation levels have been documented during dry seasons in lowland areas of South Africa and Ethiopia [16,17,19], whereas seasonal infestation patterns in other agroecological settings, including some cooler or higher-altitude regions may vary depending on local environmental conditions [14]. Moreover, excessive nitrogen application can increase *Eldana saccharina* infestation because it promotes lush, nitrogen-rich cane tissues that improve larval growth and survival, particularly when the crop is under water stress [25,26]. In contrast, moisture-conserving practices can reduce infestation by minimizing plant stress, which is closely associated with increased *E. saccharina* damage [25,27]. Other agronomic practices that influence *E. saccharina* infestation include the use of resistant varieties, avoidance of prolonged carry-over cane, targeted residue and stubble management in heavily infested fields, and habitat-management approaches such as push-pull systems [25]. Older sugarcane (> 12 months) is frequently associated with higher larval densities and greater internal tissue redness, possibly because older plants provide more favorable microclimatic conditions and more suitable oviposition substrates for *E. saccharina* including dead leaf sheaths and dry leaf material [1,28,29]. In addition, cultivation of susceptible varieties, including N46 and N49, has been linked to increased infestation intensity and concentrated damage in bottom stalk part [25].

Despite extensive regional documentation, information on the status and spatial distribution of *E. saccharina* in Tanzania remained obscured. The existed information were mainly speculations on the history of occurrence and a few unpublished reports that were insufficiently quantitative with limited evidence on infestation incidence, intensity, and within-stalk-damage patterns across Tanzanian agroecological zones. Given the environmental variability and recent expansion on sugarcane cultivation into new production areas, an updated assessment of the pest’s status was required. Therefore, this study aimed at establishing the status of *E. saccharina* in Tanzania by quantifying infestation across high, medium, and low altitude agroecological zones. Specifically, the study assessed larval density (number of *E. saccharina* per 50 stalks), percentage of stalks bored, percentage of internal stalk tissue damage, and distribution of bored plant parts to determine spatial variation in infestation intensity and damage severity [22,25,30]. The study also explored the sugarcane varieties that contributed to the increased *E. saccharina* infestations across the altitudes. By providing a cross-sectional evaluation across environmental gradients, the findings have direct implications for pest surveillance and field-level management, thus, contribute fundamental information necessary for deployment of strategic zone-specific management of the pest in Tanzania sugarcane production systems.

## 2. Materials and methods

### 2.1. Description of the study area

**Fig 1:**
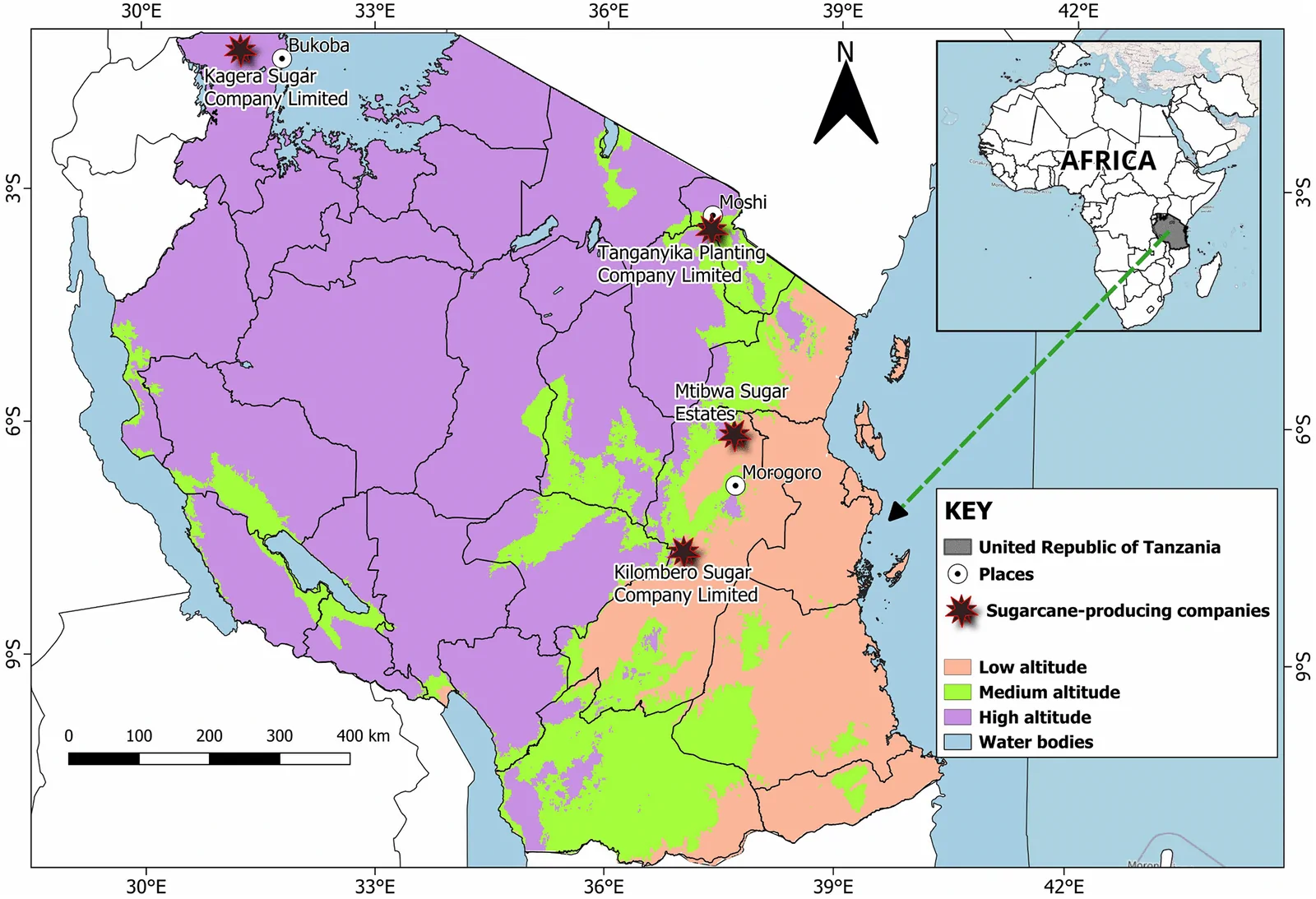
A map of the study sites showing altitude classes and sugarcane-producing company locations in Tanzania. The map was created by the authors using QGIS software (version 3.44.8) from geographical coordinates collected during the study. Administrative boundary data were obtained from shape files from the National Bureau of Statistics (NBS) available at (https://www.nbs.go.tz/statistics/geographic-information-system-gis), a public national institution, and are publicly available for use under CC BY 4.0 or equivalent open license.

The study was conducted across three agroecological zones categorized by altitude: high, medium, and low [3]. The low-altitude area included sugarcane fields situated at altitudes below 600 meters above sea level (m a.s.l), while fields at elevations between 601 and 1000 m a.s.l were classified as medium altitude. The high-altitude zone comprised sugarcane fields located above 1000 m a.s.l [31]. Each altitude zone represented sugarcane fields belonging to different sugarcane producing companies (Table 1). The companies included Kagera Sugar Company Limited, located at een betwhigh altitude S 1° 00’ to 2° 45’, and E 30° 25’ to 32° 40’ at altitude of 1400 m a.s.l in Kagera Region northwestern part of Tanzania; Tanganyika Planting Company Limited, located at medium altitude S 3° 45’ and E 37° 37’ at an altitude of 950 m a.s.l in Kilimanjaro Region in northeastern Tanzania; and two companies, Kilombero Sugar Company Limited and Mtibwa Sugar Estates at low-altitude zone, located at S 7° 44’, E 37° 00’ and S 6° 09’, E 37° 50’ respectively, in Morogoro Region in eastern Tanzania (Fig 1). High-altitude zone is characterized by relatively cooler conditions, temperature ranging from 20 °C to 28 °C, and receives biannual rainfall of about 500–2000 mm annually [22,32]. The medium-altitude zone experiences intermediate climatic conditions where annual temperatures range from 17 °C to 29 °C and rainfall is also bimodal, ranging from 600 to 2100 mm [33]. The zone receives long rains from March to May and short rains from October to December. The low-altitude zone is relatively warmer, with temperatures of 18–30 °C, receiving bimodal rainfall ranging from 600 to 1800 mm annually [34]. Across these altitudes, environmental differences in temperature and rainfall distribution create distinct ecological settings for assessing *E. saccharina* infestation.

**Table 1:** Main characteristics of the study area representing low, medium, and high altitude agroecological zones in Tanzania.

Altitude	Company/Estate	Region	Approximate altitude (m a.s.l)	Environmental characteristics	Number of sampled blocks
High	Kagera Sugar Company Limited	Kagera	1400	Cooler conditions, higher elevation, distinct rainy and dry periods	325
Medium	Tanganyika Planting Company Limited (TPC	Kilimanjaro	950	Intermediate elevation and temperature conditions	175
Low	Kilombero Sugar Company Limited	Morogoro	<600	Warmer lowland conditions	70
	Mtibwa Sugar Estates	Morogoro	<600	Warmer lowland conditions	141

### 2.2. Experimental design

A stratified, multistage cross-sectional sampling design was implemented in the survey undertaken to assess and quantify *E. saccharina* infestation across altitudes [35]. Altitude (high, medium, and low) constituted the primary strata, within each stratum, sugarcane fields in a company serving as the sampling frame. Sugarcane block disposition maps were obtained from each company and used to generate a complete list of cultivated units. This was followed with selection of blocks using a finite population sampling approach. The required number of blocks per stratum was estimated using finite population formula by Yamane [36] as follows:


$$\begin{array}{c} n=\frac{N}{(\text{1}+N(e{)}^{\text{2}}}. \end{array}$$ (1)

Whereby n is the required sample size, N is the total number of cultivated blocks in respective sugarcane-producing company, and ``e" is the allowable margin of error (set at 5%).

From the total numbers of cultivated blocks which was 2933 blocks computed from 2022 units in the high altitude, 317 units in the medium altitude, and 594 units in the low altitude, the established sample sizes were; 325 blocks in the high altitude, 175 blocks in the medium altitude, and 211 blocks in the low altitude zones.

Field surveys were conducted during locally defined rainy and dry seasons in 2025 to assess *E. saccharina* infestation under contrasting environmental conditions. These sampling periods were based on the rainfall patterns of each altitude class and were not assumed as priori to represent peak infestation periods. Assessments were performed in February-March (rainy season) and May-June (dry season) at high altitude zone, March-April (rainy season) and July-August (dry season) at the medium altitude and March (rainy season) and August-September (dry season) at the low altitude. This seasonal framework was used because rainfall influences sugarcane growth, crop moisture status, and plant stress, which in turn affect the suitability of the crop to *E. saccharina* oviposition, larval establishment, survival, and internal stalk tissue damage [14,25,37]. Since rainfall amount and season distribution differ among locations, locally defined rainy and dry periods were used to compare infestation patterns across the three study altitude classes.

### 2.3. Sampling and sampling design

Random sampling was made from the complete list of identified blocks to ensure spatial coverage across the entire estate. Sugarcane crop aged ≥ 3 months were selected for assessment targeting the stalks that have developed sufficient internodes and tissue structure to allow *E. saccharina* larval boring and establishment for detection of infestation and internal damage as per other workers [25,38]. Each randomly selected block constituted a primary sampling unit. Because the study was designed to quantify *E. saccharina* infestation under existing commercial production conditions, crop age (months after planting) and sugarcane variety were not used as pre-stratification criteria during block selection. Instead, these variables were recorded for each randomly selected blocks at the time of sampling. Consequently, the distribution of crop ages (months after planting) and varieties was not uniform across altitude classes, but reflected the actual planting structure within the surveyed estates. In this study, crop age refers to the number of moths after planting at the time of sampling, rather than cutting or harvest age. Assessment of *E. saccharina* infestation within blocks followed the standardized field survey protocol developed by SASRI (2023). Within each selected block, sampling was conducted along diagonal transects to ensure representative spatial coverage. A total of 50 sugarcane stalks were randomly selected per block, distributed evenly along the transects extending from one corner of the block to the opposite corner. To capture both edge and central field conditions, 40 stalks were sampled along the block peripheral (edges) at regular intervals, while an additional 10 stalks were sampled along the central transect. At each sampling point, stalks were selected at random and assessed for infestation and damage parameters following the established protocol. This systematic spatial altitude reduced edge or clustering effects and ensured coverage of both peripheral and interior block sections.

Sampling was conducted at the block level, with block sizes ranging from 5 to 22 ha. Although the SASRI protocol recommends sampling up to 100 stalks in blocks of ≤ 5 ha, estate management restricted destructive sampling to a maximum of 50 stalks per block. Consequently, a fixed sample size of 50 stalks was applied uniformly across all blocks, regardless of block size. This approach ensured methodological consistency and comparability of infestation estimates across blocks and altitudes, as each block constituted the primary management unit in the estates and was therefore treated as the independent sampling unit for infestation assessment.

### 2.4. Data collection

**Fig 2:**
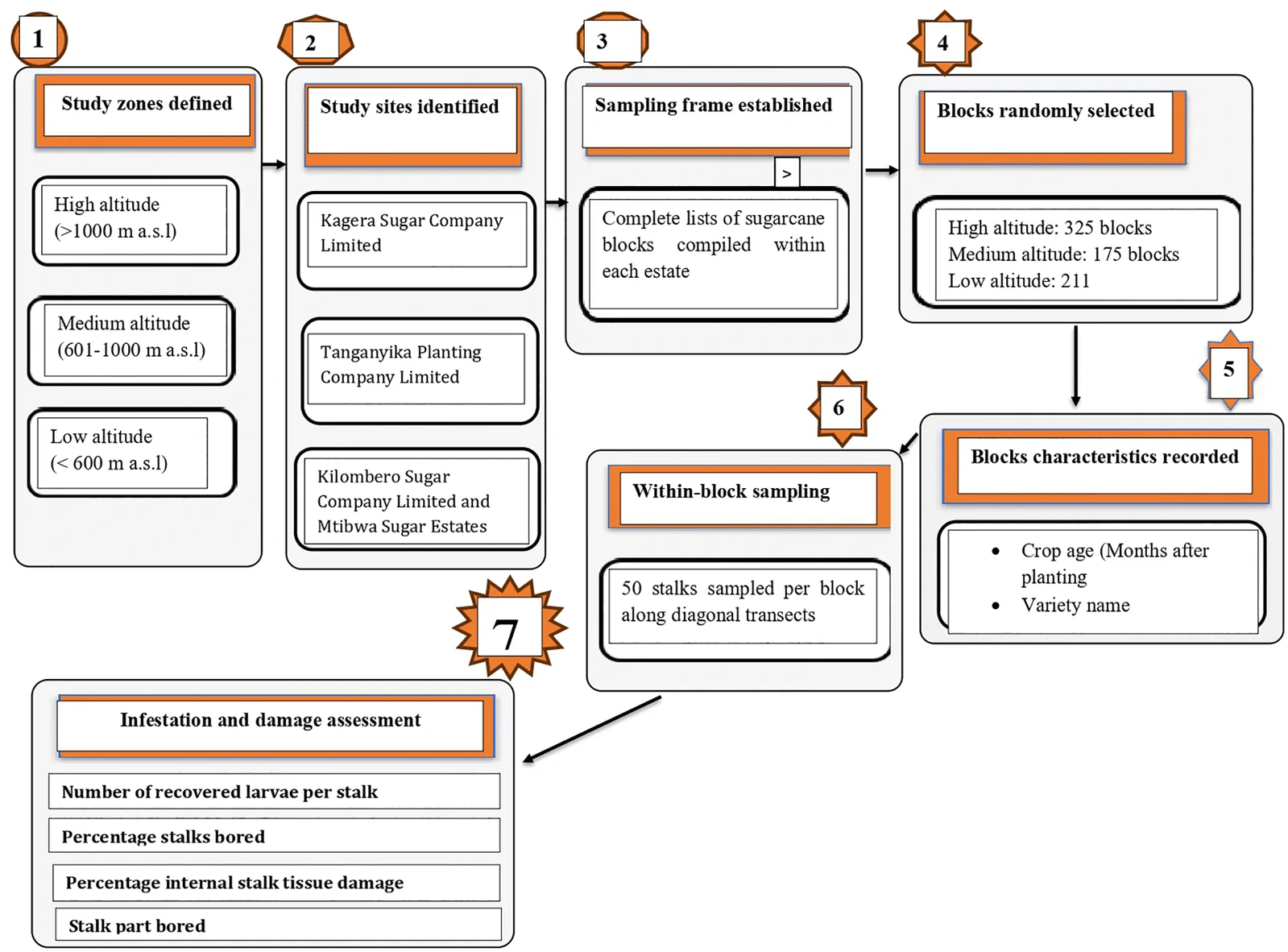
Flow chart of the sampling protocol used to assess *E. saccharina* infestation in sugarcane fields across three altitude classes in Tanzania.

Within each sampled block, 50 sugarcane stalks were randomly selected and assessed individually, each stalk was cut approximately 5 cm above ground level and initially examined for visible signs of *E. saccharina* infestation, including characteristic entry or exit holes. The position of damage along the stalk was recorded as bottom, middle, or top. Each stalk was then split longitudinally into two halves (half of four parts) to allow internal inspection. The total number of internodes per stalk and the number of bored internodes were recorded. The presence and number of *E. saccharina* larvae and/or pupae were documented. Internal tissue damage was quantified by measuring the length of red discoloration within the stalk (stalk length red (m), which served as proxy for pest-induced internal tissue damage. The recorded data therefore included block name, sugarcane variety, collection date, crop age (months after planting), stalk length (m), number of internodes per stalk, number of internodes bored, stalk length red (m), position of damage along the stalk, and number of *E. saccharina* larvae and/or pupae detected. The damage recorded in this study represents natural field incidence of *E. saccharina* infestation rather than experimentally induced injury. Field symptoms included bored stalks, internal reddish discoloration of stalk tissues, and larval occurrence within stalks [18,25]. These symptoms are consistent with stalk-boring damage caused by E. saccharina. Stepwise flow-chart of the sampling protocol is presented in Fig 2 below for clarity.

### 2.5. Ethics statement

This study methodology was approved by the Ethics Review Board of the College of Agriculture, Sokoine University of Agriculture, Tanzania (Approval Number: SUA/DPRTC/PCS/D/2022/0003/04). The study did not involve human participants, human biological materials, personal data, or vertebrate animals. Therefore, informed written or verbal consent from individual participants was not applicable. The study involved field assessment of *Eldana saccharina* infestation and sugarcane stalk damage in commercial sugarcane fields. Permission to access the fields and conduct destructive sugarcane stalk sampling was obtained verbally from the management of the respective sugarcane-producing companies before data collection.

### 2.6. Data analysis

Raw field data were first summarized into infestation and damage metrics following standard procedures described by the South African Sugarcane Research Institute SASRI (2023). Infestation intensity was expressed as the number of *Eldana saccharina* larvae and/or pupae per 50 stalks (e/50 stalks). Boring intensity was calculated as the proportion of internodes bored per stalk, while internal tissue damage was quantified as the proportion of the stalk length exhibiting red discoloration (total stalk length red/total stalk length). In addition, the presence of boring along the stalk was categorized by position (bottom, middle, top) for subsequent analysis (S1 Table).

Prior to modelling, the distribution of the response variables was examined using graphical and diagnostic methods to guide model selection. All responses were analysed within a generalized linear mixed modelling (GLMM) framework to account for abnormal distribution of data and the hierarchical sampling design. Count data were modelled using a negative binomial error distribution with a log link function, as this distribution accommodates the discrete, non-normal and overdispersion characteristics of count responses. Proportion data (bounded between 0 and 1) were analysed using GLMMs with a beta error distribution and logit link function.

Specifically, infestation intensity (e/50 stalks) was analysed using GLMMs with a negative binomial distribution and log link function in R using glmmTMB package [39]. Season (rainy and dry), altitude (high, medium and low), and crop age (centred continuous variable) were included as fixed effects with their interactions, while Block names were included as random factor to account for the hierarchical sampling structure and non-independence of observations within blocks. The fitted model was:


$$\begin{array}{c} \text{log }(\mu )=\;\beta_{\text{0}}+\beta_{\text{1}}\text{season}+\beta_{\text{2}}\text{altitude}+\beta_{\text{3}}\text{crop age}+\text{ interactions}+b_{block} \end{array}$$ (2)

Where (µ) is the expected number of *E. saccharina* individuals per 50 stalks, $\beta_{\text{0}}$ is the intercept, β terms represent fixed effects and their interactions, and $b_{block}$ is the random effect associated with block.

A count-based sensitivity analysis was conducted in R statistical software to evaluate the adequacy of the 50 stalks sampling protocol. Since infestation was analyzed as *Eldana saccharina* larval and/or pupae count per 50 stalks, detection probability was estimated on the count scale using the fitted negative binomial distribution model. The probability of detecting at least one larva/pupa was calculated across a range of expected mean counts and compared between the observed 50-stalk and a hypothetical 100-stalk sampling protocol. This analysis assessed whether 50 stalks were sufficient for detecting field-level infestation patterns and identified the potential risk of under-detecting very low of highly localized infestation.

Proportion-based response variables (internal tissue damage and boring intensity) were analyzed using GLMMs with a beta error distribution and logit link function. Seasons, altitude and crop age (centered continuous) were included as fixed effects, together with their interactions to evaluate whether damage severity varied across altitudes and developmental stages while block name was fitted as a random factor. The fitted models were:

i Beta model for percentage stalk length red

$$\begin{array}{c} \text{logit }(\pi )=\text{season x altitude x poly }\left(\text{crop ag}{\text{e}}_{\text{c}},\;\text{2}\right)+{\text{b}}_{\text{block}} \end{array}$$ (3)

Where 𝜋 is the expected percentage of stalk length reddened and$\;b_{block}$ is the random effect of the block. The polynomial term for crop age was included to accommodate potential non-linear age effects.

ii Binomial model for percentage stalk bored

$$\begin{array}{c} \text{logit }(\theta )=\text{season x altitude x crop ag}{\text{e}}_{\text{c}}+{\text{b}}_{\text{block}} \end{array}$$ (4)

Where θ is expected proportion of the internodes bored and ${\text{b}}_{\text{block}}$ is the random block effect

Boring incidence along sugarcane stalk parts (bottom, middle, and top) was also analysed using a generalized linear mixed model (GLMM) with a binomial error distribution and logit function, as the response variable was binary (presence/absence of boring). Stalk part, season, altitude, and their interactions were included as fixed effects to evaluate dependent variation in infestation along the sugarcane stalk. Block name was fitted as a random factor to account for non-independence of observations within sampling units. The variation in boring incidence along sugarcane stalk parts was modelled as:


$$\begin{array}{c} \text{Logi}{\text{t}}_{\text{pijkl}}=\;\beta_{\text{0}}+\;\beta_{\text{1}}\text{par}{\text{t}}_{\text{i}}+\beta_{\text{2}}\text{seaso}{\text{n}}_{\text{j}}+\;\beta_{\text{3}}\text{altitud}{\text{e}}_{\text{k}}+\;\beta_{\text{4}}\text{crop ag}{\text{e}}_{\text{l}}+\text{interactions}+{\text{b}}_{\text{block}} \end{array}$$ (5)

Where pijkl is the probability of boring occurrence, β_0_ is the intercept, β terms represent fixed effects and their interactions, and $b_{block}$ is the random effect associated with block.

The same identified blocks were assessed during the rainy and dry seasons. Each observation represented one block-season determination based on 50 sampled sugarcane stalks (S2 Table). Because the survey was conducted under commercial field conditions, planting dates differed among estates and blocks; therefore, the exact crop ages (months after planting) represented the dataset varied among altitudes and seasons (S2 Table). Thus, crop age was treated as a continuous covariate in the GLMM rather than a categorical treatment [39,40]. Each block-level record represented one infestation determination based on 50 sampled stalks, and all observations were analysed jointly in a single model including season, altitude, crop age (months after planting), and their interactions [39].

Model adequacy was assessed using simulation-based residual diagnostics implemented in the DHARMa package [41], including tests for dispersion, zero inflation, and deviations from model assumptions, supported by visual inspection of residual patterns The statistical significance of fixed effects and their interactions for infestation intensity and proportional based responses was assessed using likelihood ratio tests. For the binomial model of boring incidence along stalk parts, statistical significance was assessed using ${\text{Type III Wald x}}^{\text{2}}$ tests implemented in the car package [42]. Where significant effects were detected, post hoc pairwise comparisons of estimated marginal means were performed using the emmeans package [43], with Tukey-adjusted p-values. Estimated marginal means were presented with 95% confidence intervals (p ≤ 0.05).

An internal validation analysis was also conducted to assess whether the field damage indicators were biologically consistent with observed *E. saccharina* infestation. The number of larvae and/or pupae recorded per 50 stalks was correlated with measured damage variables, including percentage stalks bored, percentage stalk length red, total red stalk length, and total number of internodes bored. Because larval count and damage variables were non-normally distributed and included zero values, Spearman’s rank correlation was used. The Benjamin-Hochberg procedure was applied to adjust P values for multiple comparisons.

The influence of sugarcane variety on *E. saccharina* infestation was analyzed separately from the main ecological model because varieties were unevenly distributed across altitude categories [39]. Since several varieties occurred only within specific altitude class (S3 Table), variety was treated as nested within altitude by creating an altitudexvariety interaction factor. Infestation intensity was then analyzed using the number of *E. saccharina* larvae per 50 stalks as the response variable. Prior to analysis, altitude-variety combinations with fewer than five observations and ≥ 90% zero counts were excluded to avoid unstable estimates and convergence problems [40,44]. A negative binomial generalized linear mixed model was fitted using altitude, season, their interaction, and nested variety as fixed effects, with block include as a random factor to account for non-independence of observations within sampling block. A zero-inflated negative binomial model was also fitted and compared with the standard negative binomial model using Akaike’s Information Criterion. Model fit was evaluated using simulated residual diagnostics in DHARMa R package. Estimated marginal means were then generated for varieties within each altitude class, and Tukey-adjusted pairwise comparisons were summarized using compact letter displays. Predicted means and 95% confidence intervals were plotted to show relative varietal infestation levels within each altitude class. Thus, the varietal influence on *E. saccharina* infestation was modelled as:


$$\begin{array}{c} Log_{\mu ijklm}=\beta_{0}+\beta_{1}season_{i}+\beta_{2}altitude_{j}+\beta_{3}crop\;age_{k}+\beta_{4}\left(altitude_{j}:variety_{l}\right)+b_{block_{m}} \end{array}$$ (6)

Where $\mu_{ijklm}$ is the expected number of *E. saccharina* larvae per 50 sampled stalks, $variety_{j}$ represents variety nested within altitude, and $b_{block_{m}}$ is the random block effect to account for non-independence among observations collected from the same sugarcane block and to capture unmeasured block-level variability such as management history, soil conditions, microclimate, and local pest pressure.

## 3. Results

### 3.1. Sensitivity analysis of the 50-stalk sampling protocol

The count-based sensitivity analysis showed that detection probability increased with expected mean *E. saccharina* larval count per 50 stalks. When expected infestation was very low, detection probability under the 50-stalk protocol was limited. For instance, at expected mean counts of 0.5 and 1 larva per 50 stalks, the probability of detecting at least one individual were 36.4% and 56.4%, respectively. Detection improved under a hypothetical 100-stalk protocol, reaching 59.5% and 81% at the same expected mean counts. However, at an expected mean count of 5 individuals per 50 stalks, detection probability was already high under the 50-stalk protocol, reaching 93%, compared with 99.5% under 100-stalk sampling protocol. At expected mean counts of 10 and 20 individuals per 50 stalks, detection probabilities under 50-stalk sampling were 97.9% and 99.5%, respectively. These results indicate that the 50-stalk sampling protocol was adequate for detecting moderate to high infestation levels, but less sensitive for very low or highly localized *E. saccharina* infestation.

### 3.2. Model-fit statistics for negative binomial generalized linear mixed model fitted to *Eldana saccharina* infestation count for 50 stalks per block

The model was fitted using 1,422 observations and 698 block-level random-effect groups. The model converged successfully, with the lower value of Akaike Information Criterion (AIC) of 3515.31, Bayesian Information Criterion (BIC) of 3588.95, log-likelihood of −1743.66, and a negative-binomial dispersion parameter of 2.30. The random-intercept variance for block identity was 1.953, corresponding to a standard deviation of 1.398, indicating substantial unexplained block-level heterogeneity in *Eldana saccharina* infestation.

### 3.3. Variations in *E. saccharina* infestation across altitudes

Field infestation was confirmed by the presence of bored stalks, internal stalk tissue red, and larvae within sugarcane stalks, indicating natural *E. saccharina* incidence in the surveyed fields. The Likelihood Ratio Test (LRT) from the negative binomial generalized linear mixed model (GLMM) revealed that *E. saccharina* infestation varied significantly (P < 0.05) with season, altitude, and crop age (Table 2). The fixed effects of season, altitude, and crop age were all significant (P < 0.001). Significant two-way interactions were also observed between season and crop age $\left({\text{X}}^{\text{2}}=\text{23}.\text{57},\text{ P }<\;\text{0}.\text{001}\right)$ and between altitude and crop age ($X^{\text{2}}=\text{1}\text{2}.\text{8}\text{1},\;P=\text{0}.\text{0}\text{0}\text{1}\text{7}$), indicating that the effect of crop age on infestation differed across seasons and altitude classes. In contrast, the season X altitude interaction was not significant ${(\;x}^{\text{2}}=\text{0}.\text{3}\text{9},\text{ P}=\text{0}.\text{8}\text{2}$) suggesting that seasonal differences in infestation were not consistent across altitude classes when crop age was not considered (Table 2). Further to this, a three-way interaction between season, altitude, and crop age was significant ${(\;x}^{\text{2}}=\text{9}.\text{5}\text{8},\text{ P}<\text{0}.\text{0083})$.

**Table 2:** Likelihood Ratio Test (LRT) evaluating the effect of season, altitude, crop age, and their interactions on *E. saccharina* infestations in Tanzania.

Source of variation	Chi-square ($x^{2})$	P-value
Season	56.44	<0.001
Altitude	341.42	<0.001
Crop age	43.91	<0.001
Season*altitude	0.39	0.82
Season*crop age	23.57	<0.001
Altitude*crop age	12.81	0.0017
Season*altitude*crop age	9.58	0.008

The post-hoc analyses (Table 3) indicated that the significant season, altitude, and crop age interaction was primarily driven by a strong positive effect of crop age on *E. saccharina* infestation at high altitude during both dry and the rainy season (P < 0.002 and P < 0.001, respectively). Additionally, during the dry season, sugarcane crops aged 3–9 months after planting were significantly (P < 0.001) infested whereas the older crops aging 12–18 months after planting were significantly infested during the rainy season, whereas crop-age effects were non-significant at low and medium altitudes and during the dry season.

**Table 3:** Post-hoc pairwise seasonal contrasts of predicted *E. saccharina* infestation across altitudes and crop ages. Positive Z-ratios indicate greater model predicted infestation during the dry season, whereas negative Z-ratio values represent greater predicted infestation during the rainy season. Significance differences were assessed at P ≤ 0.05.

Altitude	Crop age	Dry/Rain ratio	Z-ratio	P – Value
Low altitude	3	0.85	−0.19	0.85
Low altitude	6	1.48	0.70	0.48
Low altitude	9	2.57	1.70	0.09
Low altitude	12	4.48	1.79	0.07
Low altitude	15	7.79	1.68	0.09
Low altitude	18	13.54	1.60	0.11
Medium altitude	3	10.81	1.09	0.28
Medium altitude	6	7.20	1.23	0.22
Medium altitude	9	4.80	1.32	0.19
Medium altitude	12	3.19	1.04	0.29
Medium altitude	15	2.13	0.52	0.60
Medium altitude	18	1.42	0.18	0.86
High altitude	3	9.83	6.71	< 0.001
High altitude	6	5.15	6.92	< 0.001
High altitude	9	2.70	6.43	< 0.001
High altitude	12	1.42	2.43	0.01
High altitude	15	0.74	−1.44	0.15
High altitude	18	0.39	−3.08	0.002

**Fig 3:**
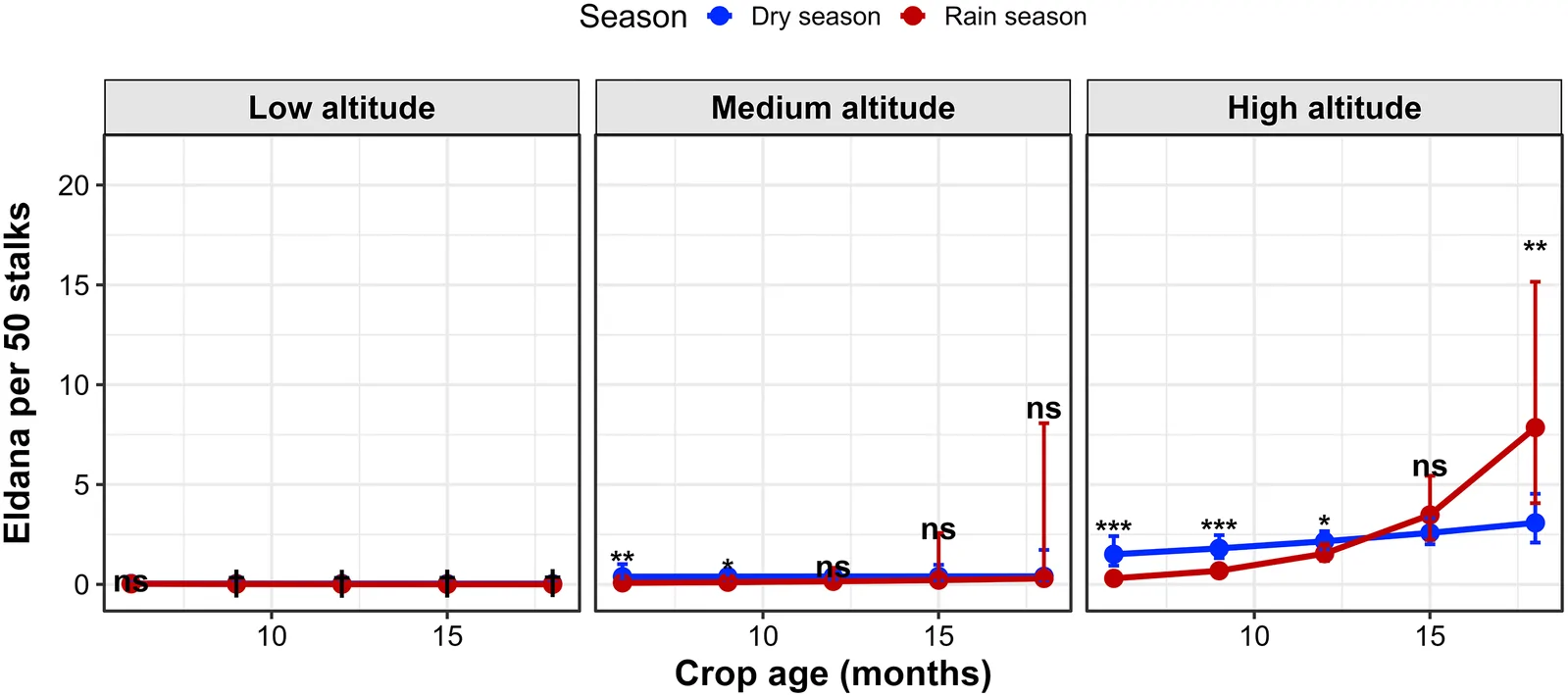
Seasonal effects on *E. saccharina* infestation across altitudes and crop ages; * P<0.05, ** P<0.01, *** P<0.001, ns = not significant.

Model-predicted infestation generally increased with crop age, but this increase was statistically evident (P < 0.01) at high altitude, particularly in older crops (>12 months) during the rainy season (Fig 3). At low altitude, infestation levels remained close to zero across all crop ages in both seasons. At medium altitude, infestation remained relatively low, although significant seasonal differences were detected only at early crop ages approximately 6 months (Fig 3), while no significant seasonal differences were observed at later crop ages, indicating weak seasonal effect.

The same altitudinal and seasonal pattern was reflected in the percentage of stalks bored (Table 4). Seasonal contrasts averaged over crop age showed no significant dry-rainy season differences in percentage stalks bored at the low altitude (estimate 0.02, P = 0.34). In contrast, the dry-rainy season contrast was negative and significant at medium altitude (estimate = −0.10, P = 0.013) and high altitude (estimate = −0.05, P = 0.004), indicating higher predicted stalk-boring incidence during the rainy season than during the dry season in these altitude classes. However, because the season X alitude X crop age interaction was significant, these averaged contrasts should be interpreted together with the age-specific predicted responses.

**Table 4:** Model estimated seasonal differences in the percentage sugarcane stalks bored within each altitude. Estimates represent the contrast between the dry and rain seasons averaged over crop age; negative estimates indicate higher predicted boring during the rainy season.

Altitude	Estimate	SE	Z-ratio	P – value
Low altitude	0.02	0.02	0.95	0.34
Medium altitude	−0.10	0.04	−2.48	0.013
High altitude	−0.05	0.02	−2.83	0.004

**Fig 4:**
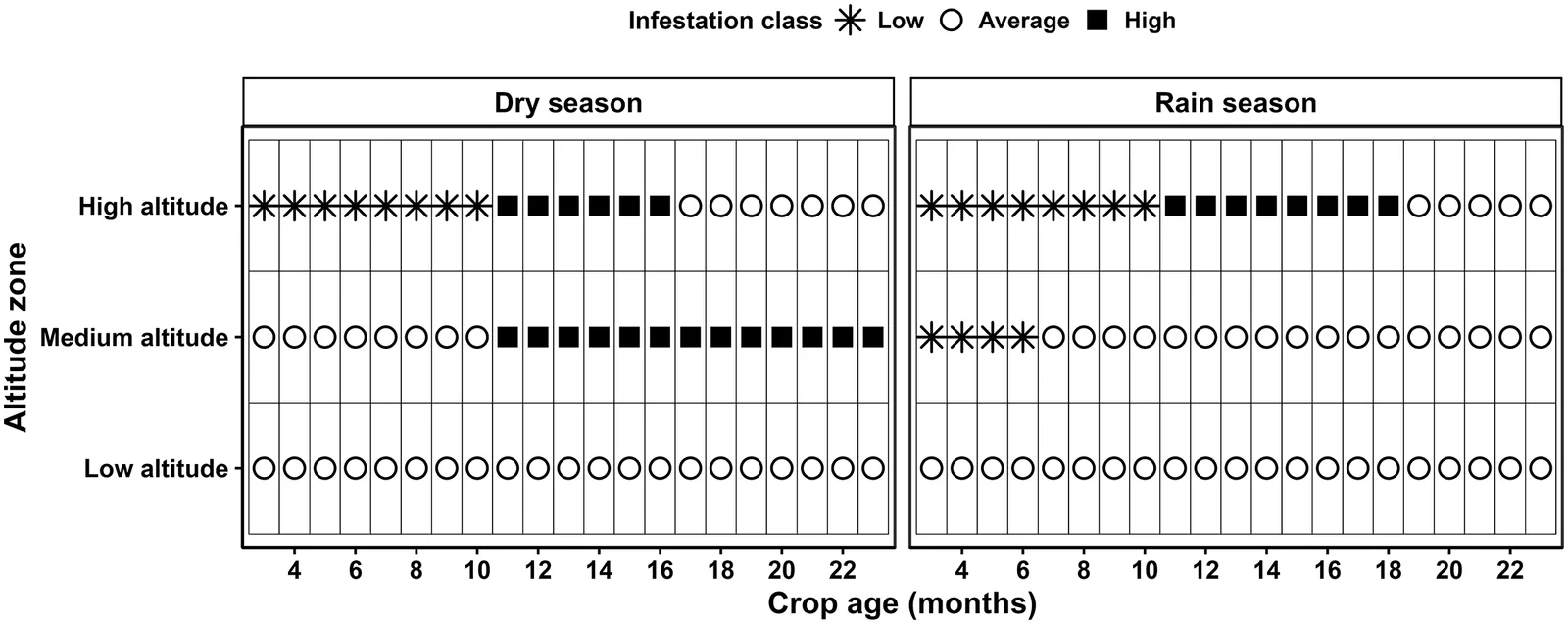
Influence of crop age on the percentage sugarcane stalk bored caused by *E. saccharina* across the seasons and altitudes.

The boring percentage was further observed to be high in both high and medium altitudes, specifically to crop ages with approximately 12 months and above (Fig 4). On the other hand, low to average boring percentages were noted in low altitude.

### 3.4. Extent of internal red stalk tissue damage caused by *E. saccharina* infestation

The likelihood ratio test (Table 5) showed that damage intensity expressed as the percentage stalk length red was highly significantly influenced by season, altitude and crop age rather than being driven by any single factor (all 𝐏<0.001).

**Table 5:** Likelihood ratio test results for effects of season, altitude, and crop age, and their interactions on percentage stalk length red caused by *E. saccharina.*

Source of variation	Chi-square ($x^{2})$	P – value
Season	480.1	< 0.001
Altitude	218.1	< 0.001
Crop age	109.1	< 0.001
Season*altitude	89.71	< 0.001
Season*crop age	14.92	< 0.002
Altitude*crop age	34.77	< 0.001
Season*altitude*crop age	11.52	<0.003

The post-hoc analyses (Table 6) showed that seasonal effects on internal tissue damage varied across altitudes. At low altitude, internal stalk damage was significantly higher during the dry season (𝐏<0.0072). In contrast, at high altitude, the negative seasonal estimate indicated significantly higher internal tissue damage during the rainy season (𝐏<0.04). At medium altitude, seasonal differences in the internal stalk tissue damage were not statistically significant.

**Table 6:** Seasonal effect on damages of internal sugarcane stalk tissues caused by *E. saccharina*. Estimates represent the contrast between the dry and rain seasons averaged over crop age; negative estimates indicate higher predicted boring during the rainy season.

Altitude	Estimate	SE	Z-ratio	P – value
Low altitude	0.07	0.03	2.69	0.0072
Medium altitude	−0.15	0.21	−0.71	0.48
High altitude	−0.06	0.03	−1.97	0.04

**Fig 5:**
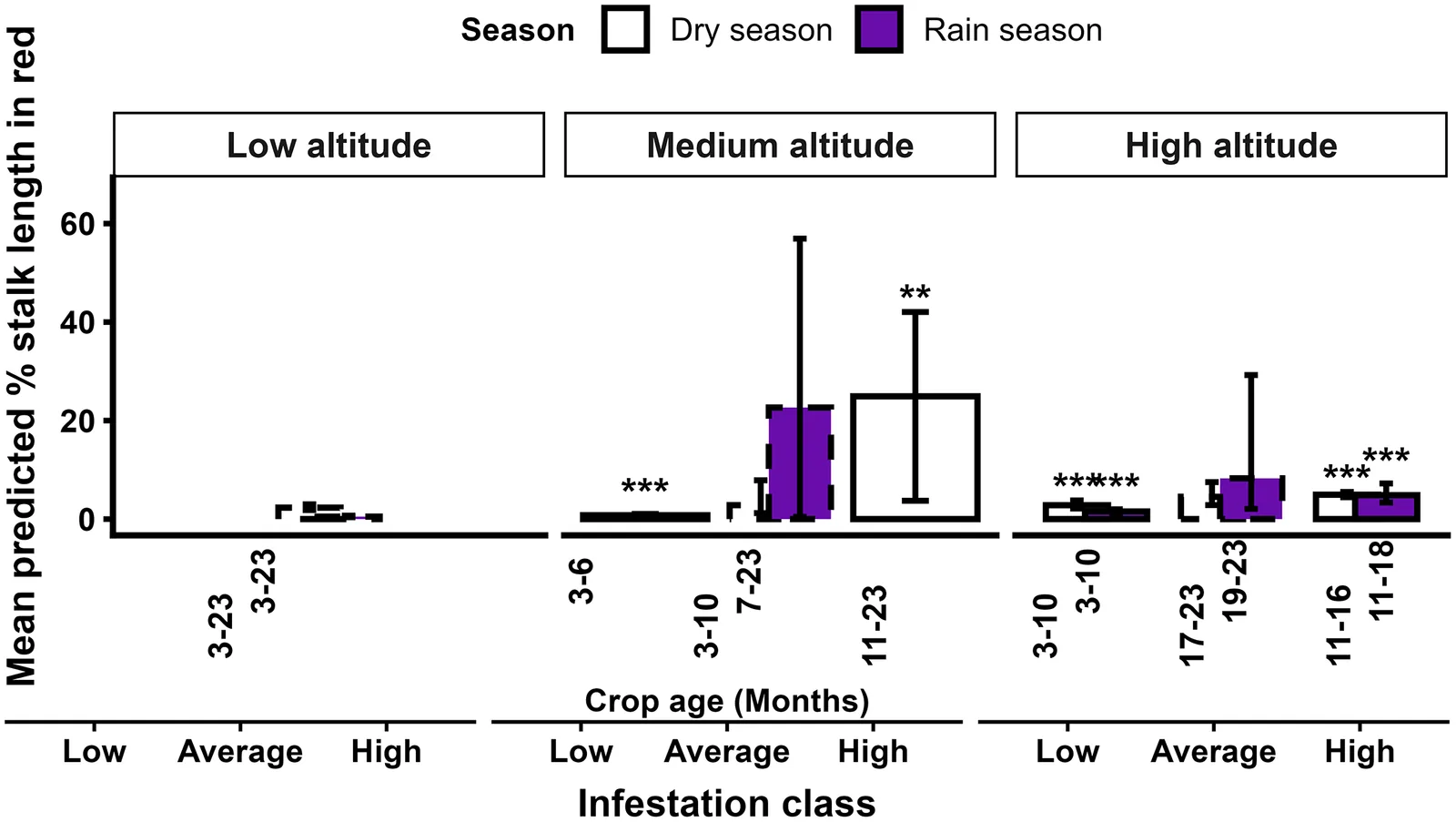
Damages of internal sugarcane stalk tissues caused by *E. saccharina* across crop ages in each season and altitude.

With respect to crop age, post-hoc analyses further indicated that the percentage of stalk length red increased with increasing crop age (Fig 5). At low altitude, internal stalk tissue damage remained predominantly within the average class across crop ages. At high altitude, the damage increased markedly with crop age, reaching significantly high levels from approximately 12 months onward in both dry and rainy seasons. In contrast, internal stalk damage tended to increase with crop age at medium altitude and significantly high infestation (P < 0.01) was observed during the dry season (Fig 5).

### 3.5. Determinants of sugarcane stalk part boring by *E. saccharina*

**Fig 6:**
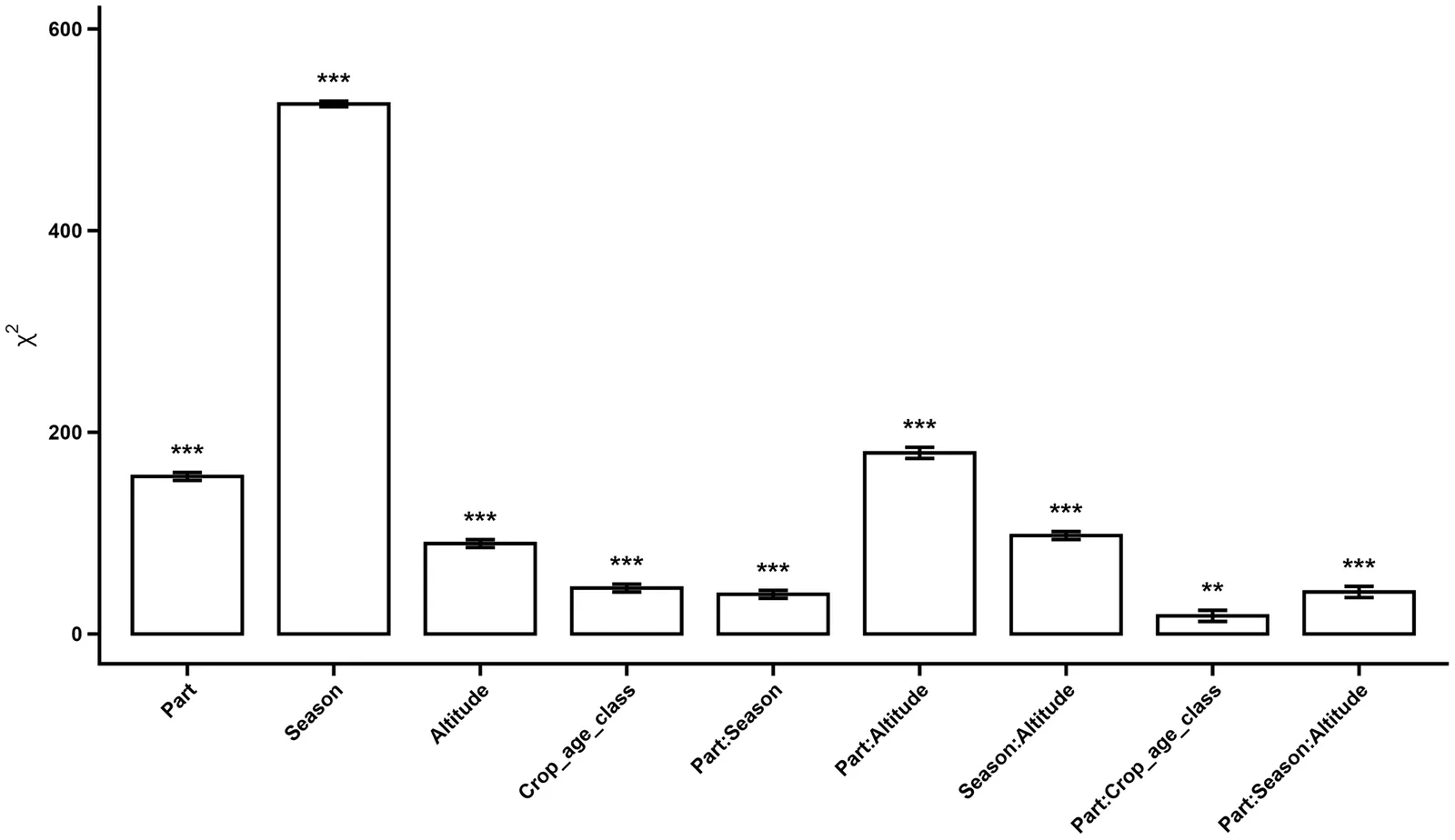
Effects of season, altitude, crop age and their interaction in different bored parts of sugarcane stalks due to *E. saccharina* infestation.

Type III Wald chi-square test revealed that the probability of *E. saccharina* boring a sugarcane stalk was strongly influenced by stalk part, season, altitude, and crop age, as indicated by highly significant main effects and the interactions (Fig 6). This confirms that infestation is not uniform within the plant across seasons and altitudes.

**Fig 7:**
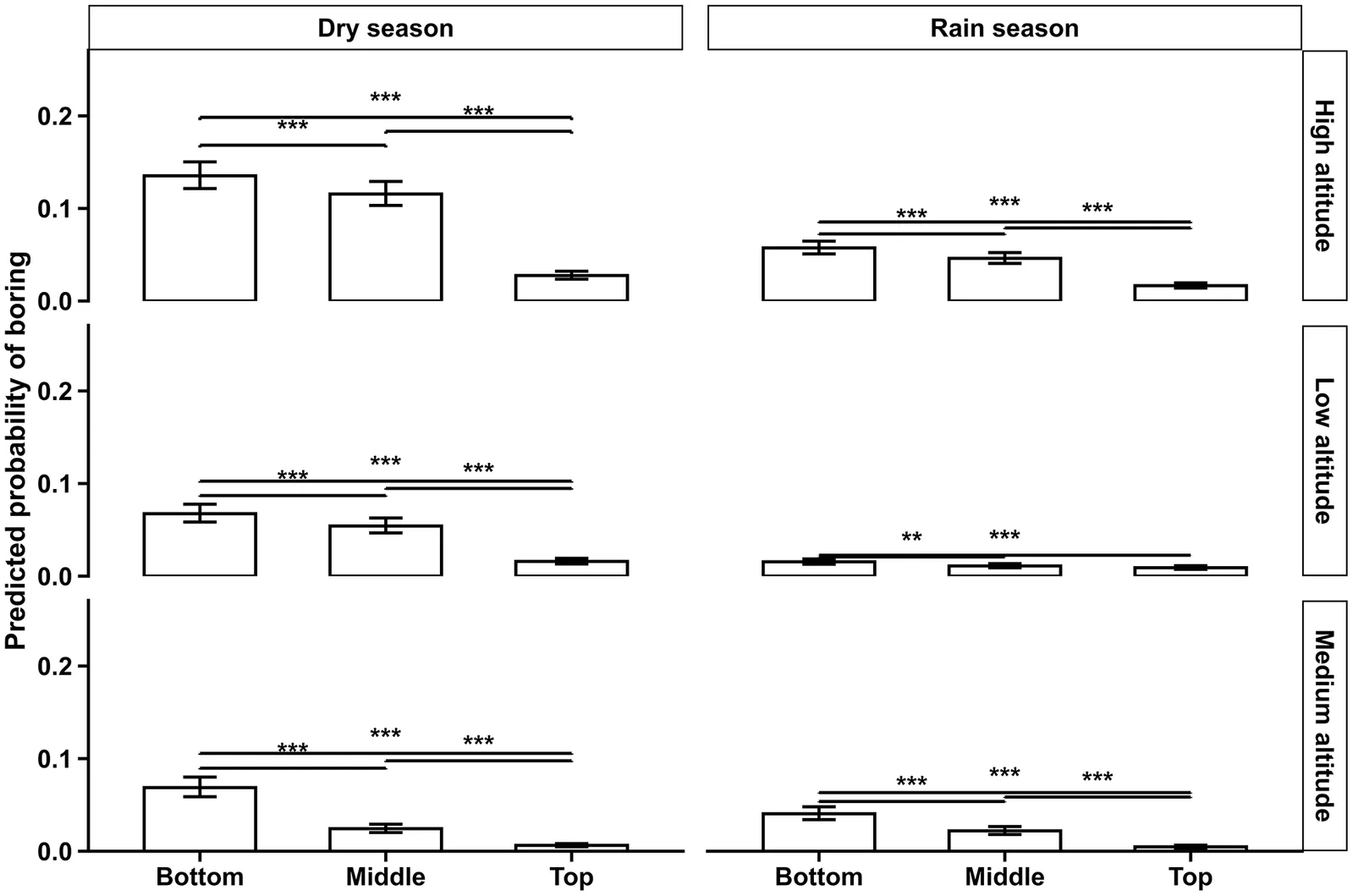
Model-predicted probabilities of *E. saccharina* boring among sugarcane stalk parts across seasons and altitudes, with bars showing estimated means ± 95% confidence intervals and brackets with asterisks indicating significant pairwise differences within each season and altitude combination.

Predicted boring probabilities of *E. saccharina* differed among sugarcane stalk parts depending on season and altitude (Fig 7). In all season and altitude combinations, the bottom stalk part exhibited the highest predicted probability of boredom, whereas the top part consistently showed the lowest values, with the middle part intermediate. Post-hoc analysis revealed significant differences among stalk parts at medium and high altitudes in both dry and rainy seasons. At low altitude, part-specific differences were less pronounced during the rainy season, where the middle and top stalk parts did not differ significantly, although both remained less bored than the bottom part. Generally, infestation followed a clear vertical gradient along the stalk, modulated by the environmental conditions (Fig 7).

### 3.6. Internal validation of sugarcane stalk-damage indicators

Internal validation of the damage assessment showed that *Eldana saccharina* larval counts per 50 stalks were positively associated with most measured sugarcane stalk-damage indicators such as percentage stalk length red, total stalk length red, percentage stalks bored, and total number of internodes bored. The strongest associations were observed between larval counts and percentage stalk length red (Spearman’s rank correlation coefficient (ρs) = 0.61, P < 0.001) and total red stalk length ((Spearman’s rank correlation coefficient = 0.60, P < 0.001). Positive correlations were also observed between larval counts and percentage stalks bored ((Spearman’s rank correlation coefficient = 0.56, P < 0.001) and total number of internodes bored (Spearman’s rank correlation coefficient = 0.56, P < 0.001). These results indicate that higher *E. saccharina* larval counts were generally associated with greater internal stalk-tissue damage.

### 3.7. Sugarcane varietal influence on *E. saccharina* infestation within altitudes

**Fig 8:**
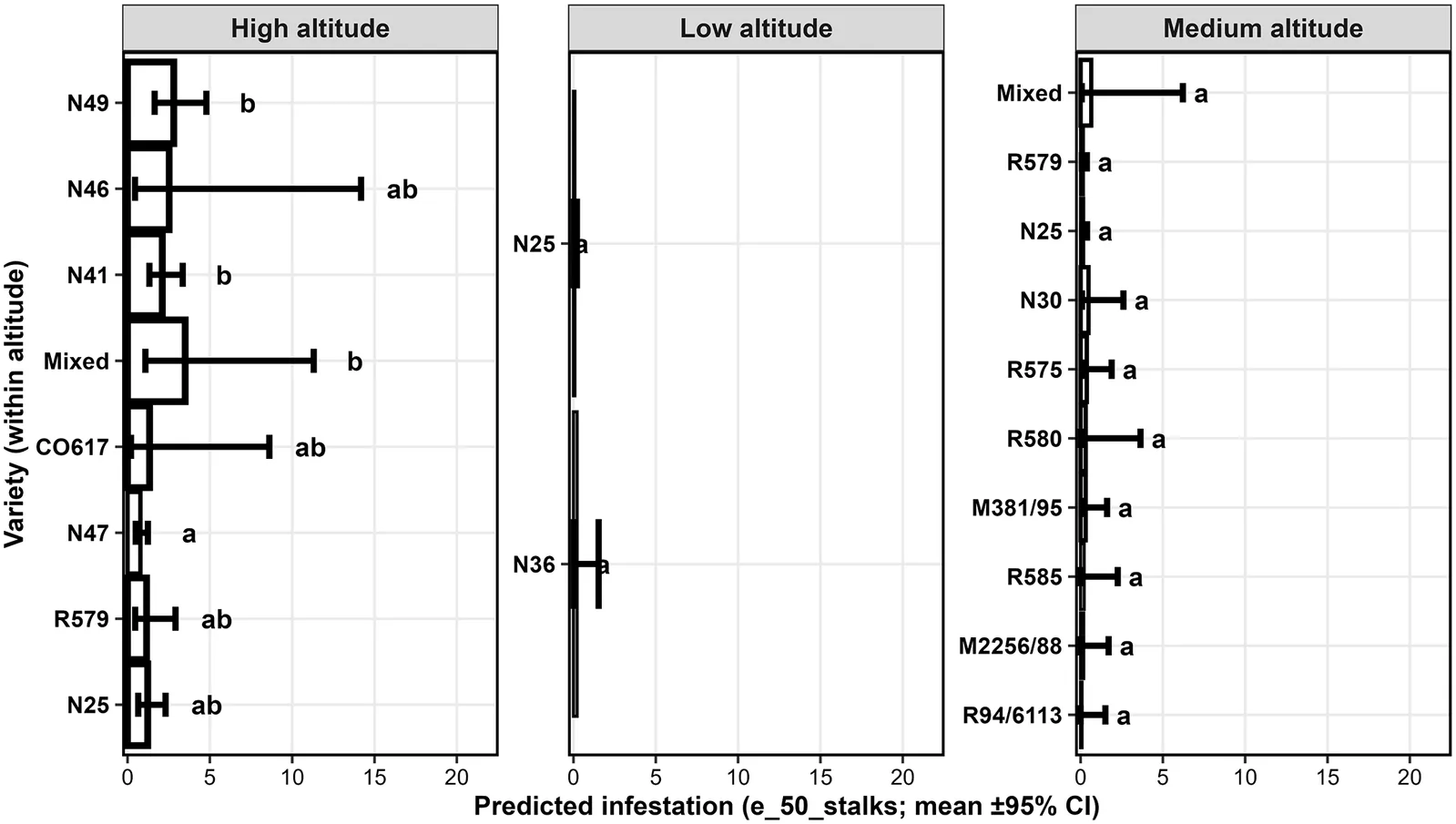
*E. saccharina* infestation intensity across altitudes and sugarcane varieties. Bars sharing the same letter suggest a statistical insignificance in infestation intensity at *P* ≤ 0.05.

In the high altitude, infestation intensity was markedly greater than in the low altitude (Fig.8). Within the high-altitude, sugarcane fields with mixed varieties, N41 and N49 exhibited the highest predicted infestation levels, although several varieties did not differ statistically from each other. In contrast, infestation at low altitude remained uniformly low, with no significant varietal differences, despite slightly higher mean counts in N36 and N25 (Fig 8). Similarly, varietal differences at medium altitude were not statistically significant, although mixed varieties and N30 showed comparatively higher numerical infestation levels.

## 4. Discussion

The presented findings suggest that *E. saccharina* infestation and resultant damages were influenced by significant interactions among season, altitude, and crop age. Infestation increased with crop age at high altitude during both rainy and dry seasons across crop ages. In this study, terms such as “greater” and “lower” infestation are used to describe relative differences among altitudes, seasons, crop ages (months after planting) and varieties, rather than locally validated economic injury thresholds. More counts of *E. saccharina* were observed in older crops (>12 months), especially during the rainy season. Despite the generally low infestations at low altitude, the recorded internal stalk tissue damage was relatively higher particularly during the dry season. At high altitude the internal damage was greater in both dry and rainy seasons, especially in older crops. Medium-altitude sites, however, was manifested with significantly greater infestation during the dry season, especially in crops older than 11 months. Greater stalk-boring intensity was recorded at medium and high altitudes with the bottom parts of the stalk being mostly affected. Fields with mixed varieties (particularly N41 and N49) exhibited greater infestation with *E. saccharina* at medium and high altitudes, while N36 and N25 sugarcane varieties had greater mean counts of the borer at low altitude.

Similar observations have been reported in northern Ivory Coast [22, 45] that *E. saccharina* infestation increased during the rainy season in sugarcane fields. Whereas their study was primarily structured around establishing the effect of cropping seasons, the present study further incorporates altitudinal gradients and crop age to increase clarity on the interacting environmental drivers of infestation intensity. Similarly, Mulcahy et al. (2023), in evaluating push-pull technology for *E. saccharina* management in South Africa, documented higher incidences of the pest in highland areas compared to mid-altitude and coastal regions, underscoring the role of altitudinal variations in shaping infestation risks. The elevated infestations observed at high altitude during the rainy season in this study may be attributed to prolonged crop growth particularly in fields exceeding 12 months, which led to thicker stalks and greater biomass favoring suitable environment for the pest survival. Such structural characteristics increase oviposition opportunities and provide favorable larval refugia, thereby enhancing establishment and survival of the pest [24]. Because some surveyed fields remained in the fields for more than 12 months before harvest, older crops could have a longer exposure period to *E. saccharina* oviposition and larval establishment. This prolonged exposure, together with more developed stalk tissues, may partly explain the greater infestation and internal stalk tissue damage recorded in older crops [24,25]. The observed increase in infestation in older crops is also biologically plausible because *E. saccharina* larvae bore into sugarcane stalk tissues, where they remain protected and continue feeding internally [28, 46]. Previous studies on the biology of *E. saccharina* have shown that females oviposit mainly in concealed sites such as leaf sheaths to which eggs hatch within a few days, and larvae subsequently enter and tunnel within stalk tissues [47, 48]. Other studies have also documented high fecundity, repeated mating capacity, and temperature-sensitive physiological responses, all of which support the potential for population build-up under favorable field conditions [47–49]. Climate-based modelling studies further indicated that temperature and seasonal conditions can influence generation turnover and phenological variations in *E. saccharina* [22, 30]. Therefore, the seasonal and altitudinal differences observed in the present study are consistent with the known biology of the pest, although direct measurements of the life-cycle parameters were not conducted. The findings should therefore be interpreted as field-level infestation patterns shaped by crop age, season, altitude-related environmental conditions, and host-plant characteristics

Studies elsewhere suggested that water stress increases susceptibility of sugarcane to *E. saccharina*, resulting in greater larva penetration and elevated boring intensity that increase the internal stalk tissue damage [25,50]. A comparable pattern was observed in the present study, whereby significantly greater internal tissue damage occurred at low altitude during the dry season, when moisture limitation was most pronounced. Under such conditions, physiological stress likely reduces stalk resistance and facilitates larvae infestation and establishment [22]. Consistent with stem borer ecology, neonates rapidly penetrate plant tissue, where the internal environment provides a buffered microclimate that enhances survival relative to exposed conditions [15,51]. However, the greater boring intensity and internal tissue damage recorded at high and medium altitudes during the rainy season likely reflect a different pattern and mechanisms that triggers boring of canes. In these environments, increased moisture and favorable thermal conditions could have enhanced *E. saccharina* survival, development, and population growth [23,30], thereby increasing infestation pressure. In summary, at high-altitude, the stronger rainy-season infestation in older crops may reflect favorable moisture conditions, suitable temperature and relative humidity, and longer crop exposure [15,47,52], which together may support oviposition, larval establishment, and population build-up. By contrast, at low altitude, dry-season conditions may increase plant stress, reduce stalk vigor, and predispose can tissues to greater visible internal damage even when live infestation counts remain low. Therefore, the two patterns should not be interpreted as contradictory, rather they suggest that *E. saccharina* infestation intensity and stalk damage severity may be driven by different combinations of pest population pressure, crop age, environmental suitability and host-plant stress.

The positive correlations between *E. saccharina* larval counts and measured damage indicators support the reliability of the field damage assessment of this study. In particular, the association between larval counts and percentage stalk length red, total red stalk length, percentage stalks bored, and bored internodes indicates that the recorded damage variables were biologically consistent with infestation intensity [46,53]. However, the correlations were moderate rather than perfect, which is expected because larval counts represent cumulative feeding damage that may persist after larvae have moved, pupate, or died [39,40].

Adult *E. saccharina* preferentially oviposit beneath older leaves of sugarcane which are predominantly located at the bottom part of the stalk [25,30]. Because eggs deposition occurs near these bottom nodes, newly hatched larvae typically penetrate the nearest accessible internodes, resulting in a greater concentration of boring in the bottom part compared to middle and top parts [54]. The vertical distribution of leaf sheaths therefore directly influences the spatial pattern of larval establishment [16]. In the present study, sugarcane crops older than 12 months exhibited significantly higher boring intensity in the bottom third part of the stalk, consistently matching the reported pattern that *E. saccharina* is a pest of mature sugarcane [53]. Available reports further suggest that females *E. saccharina* prefer to oviposit on dead leaves which concentrate around the lower stalk parts [9,53]. Thus, the significant damage to the bottom part of the stalk may reflect on the greater suitability of older cane for *E. saccharina* infestation.

Altitude may influence *Eldana saccharina* infestation through its association with temperature, relative humidity, rainfall patterns, and crop phenology [37,55]. Although life-cycle parameters were not directly measured in the present study, previous work has shown that *E. saccharina* development, survival, reproduction performance, and adult physiological traits are temperature-sensitive [49,56]. For instance, the laboratory and rearing studies commonly maintain *E saccharina* under warm and moderately humid conditions, approximately 25–33 °C and 70–75% Relative Humidity (RH), which appear favorable for survival, development, and colony maintenance [47,49]. Additionally, successful larval rearing has been reported at 26 ± 2 °Cand 72 ± 5% RH, while other experimental work has used 25 ± 1 °C and 70 ± 10% RH [47,49]. Moreover, developmental temperature also affects adult water-balance traits, with lower developmental temperatures increasing water-loss rate and reducing adult survival time, indicating that temperature can influence pest performance beyond larval development alone [48]. Therefore, differences in temperatures and moisture conditions across altitudes may influence larval development, adult emergence, reproduction, desiccation tolerance, and seasonal population build-up [19]. In the present study, the stronger infestation response observed in older crops at high altitude during the rainy season may reflect the combine effects of crop age, seasonal moisture, and altitude-related environmental suitability. However, because direct life-cycle measurements were not conducted, this interpretation should be considered biological plausible but inferential. Future studies should quantify egg development, larval duration, pupation, adult emergence, fecundity, and generation time across altitude zones to clarify how altitude gradient modifies *E. saccharina* population dynamics under Tanzania sugarcane-growing conditions.

Varietal composition may have contributed to the observed variation in *E. saccharina* infestation across altitudes [25]. In the present study, greater infestation was associated with varieties N41 and N49, particularly in high-altitude fields. These cultivars have previously been classified as susceptible under South African production conditions [25], suggesting that varietal characteristics could partly play the predisposition factor to the elevated infestation as observed in fields at high altitude. However, the specific plant traits responsible for susceptibility were not directly measured in the present study, varietal susceptibility to stalk borers may be associated with morphological and biochemical characteristics such as stalk hardness, rind thickness, fibre content, internode structure, leaf-sheath adherence, stalk moisture, and defensible compounds, which can influence oviposition preference, larval penetration, establishment, and internal feeding success [25,57,58]. For N49, previous South African variety information identifies it as susceptible to *E. saccharina* and recommends that it should not be carried over in irrigated areas [25]. The variety is also characterized by very high sucrose content, moderate fiber content, and a tendency to lodge under very high tonnages, characteristics favoring *E. saccharina* infestation preferences [57,59]. On the other hand, the response of N41 sugarcane variety to *E. saccharina* infestation may depend strongly on growing environment, crop stress, and crop age [25]. Therefore, the greater infestation recorded in fields containing N41 and N49 should be interpreted as field-level association between varietal composition and infestation intensity, rather than direct proof of the specific traits responsible for susceptibility under Tanzania sugarcane-production conditions. However, because varieties were not uniformly distributed across all altitudes and reflected estate-specific planting practices, varietal effects should be interpreted cautiously and in relation to altitude, season, and crop age.

The occurrence of susceptible varieties within production areas planted with mixed varietal stands may also influence local infestation patterns. Previous work has shown that greater pest pressure can occur where susceptible varieties are grown together with resistant or tolerant varieties [22]. Whenever highly susceptible varieties are incorporated in resistant ones, the former tends to act as focal points for *E. saccharina* oviposition with subsequent larval build-up, thereby increasing overall infestation intensity and associated internal tissue damage. This may be particularly important in older crops, where longer exposure time allows infestation to accumulate [25]. Therefore, while the results suggest that varietal susceptibility may have contributed to the higher infestation recorded in some high-altitude fields, the observed pattern likely reflects the combine influence of varietal composition, altitude-related environmental conditions, seasons, and crop age.

The obtained results have great implications for sugarcane-producers as the crop age has been reported to have a significant influence on increasing *E. saccharina* infestations across the seasons and altitude. Thus, timely harvest scheduling, especially avoiding prolonged carry-over beyond approximately 11–12 months in medium and high-altitude fields should be considered when developing an Integrated Pest Management (IPM) package against the pest. As the method has been previously observed to reduce the yield losses associated with *E. saccharina* infestation [45,60]. Furthermore, the observed variation in *E. saccharina* infestation across altitudes, seasons, and crop ages has direct implications for pest surveillance and field-level management. Infestation was consistently low in low-altitude fields, whereas medium and high altitude fields showed greater stalk-boring incidence, particularly in older crops. This suggests that monitoring programmes particularly in the medium and high altitudes should prioritize older sugarcane fields, especially those aged 12 months and above. The stronger infestation response during the rainy season at high altitude further indicates that seasonal scouting should be intensified during periods when crop age and environmental conditions coincide to increase infestation risk.

Although the present study provides important insights into the infestation patterns of *E. saccharina* in Tanzania, its scope was limited to sugarcane-producing companies located across the selected altitude gradient translated to different altitude. These companies typically grow improved varieties reported to possess intermediate to substantial resistance to *E. saccharina* [25,60], and the farming is characterized by year-round irrigation service which supplements the cane abilities to resist the pest infestation due to improved crop vigor and limited water stress which buffers the pest damage [61]. Thus, a different scenario is expected in smallholder production systems where access to irrigation and fertilization are probably limiting. Because such provisions are not commonly practiced, findings from the current study should be interpreted with caution when considering the management options for *E. saccharina* under small holder farming systems. When extrapolating these results to the broader national context, such dynamics should be taken on-board to avoid misguiding the sugarcane growers. Based on the limitations of the current study that the pest infestation trend and dynamics under small holding farming systems was not covered, we recommend that future studies should endeavor to include farmers’ fields to enable a more representative and reliable conclusion on the infestation status of *E. saccharina* in Tanzania. Moreover, the current study quantified infestation status and stalk-boring incidence, it did not estimate yield losses associated with *E. saccharina* damage such as sucrose reduction, or monetary losses, thus, the absence of direct economic loss estimates is also a limitation of this study. Future work should combine infestation assessments with yield, cane-quality, and sucrose measurements to quantify economic injury levels under Tanzanian production conditions. Such studies would allow infestation thresholds to be linked to economic losses and would support more precise decision-making for *E. saccharina* management. Furthermore, although the results show clearly variation in *E. saccharina* infestation with altitude, season, crop age, and varietal composition, the study did not directly determine whether these patterns represent long-term typical population behavior in each landscape. This is because infestation may vary among years depending on rainfall distribution, temperature, relative humidity, crop management, harvest scheduling, variety development and pest carry-over from surrounding fields. Therefore, confirming the typicality and persistence of these patterns will require multi-year landscape-level monitoring that integrates pest abundance, crop phenology, weather conditions, varietal distribution, and management practices across repeated production cycles.

A further limitation of the study was the restriction imposed by sugarcane-producing companies on destructive stalk sampling. Although South Africa Sugarcane Research Institute (SASRI) protocol recommends sampling 100 stalks per 5 ha, only 50 stalks per block were permitted in the present study because of concerns about crop damage in commercial fields. This reduced sampling intensity may have lowered the probability of detecting very low, patchy, or highly localized *E. saccharina* infestations, and may therefore have contributed to underestimation of infestation in some blocks. Nevertheless, the same sampling procedure was applied consistently across altitudes, seasons, and crop age, allowing standardized comparison of infestation patterns across the surveyed estates. Future studies should also consider larger destructive samples, repeated within-season sampling methods to improve detection of spatially patchy infestations under commercial production conditions.

## 5. Conclusion

*Eldana saccharina* infestation has been demonstrated to be strongly structured by season, altitude, and crop age. The high-altitude agroecological zone consistently recorded the highest *E. saccharina* infestation in both seasons. Elevated infestation levels were observed during the dry season across younger than or approximately equal to 9 months old stalk, whereas in the rainy season infestation was predominantly concentrated in older crops (>12 months), showing elevated boring intensity and internal stalk tissue damage. Although infestation levels were lower at the low-altitude, internal tissue damage was comparatively high, indicating that visible pest density does not always reflect physiological injury. Damage was concentrated in the bottom stalk part, while the top sections were least affected. Mixed-variety planting, especially involving N41 and N49, increased susceptibility at high altitude. These findings suggest that management practices against *E. saccharina* should be tailored to specific agroecological conditions and crop growth stages rather than applying uniform interventions across all production areas.

## Supporting information

S1 Table Raw data used for the analysis process. (PDF)

S2 Table Distribution of block-season observations and stalks assessed across crop age categories, altitudes and seasons. (PDF)

S3 Table Distribution of sugarcane varieties across sampling sites. (PDF)
