# Supplementary material for: Factors affecting the infestation status of African sugarcane stalk borer (Eldana saccharina Walker) (Lepidoptera: Pyralidae) in Tanzania
Source: PLoS One. 2026 Aug 31;21(8):e0355844. doi: 10.1371/journal.pone.0355844 (PMC13528964; doi:10.1371/journal.pone.0355844)
Supplement: Supplementary file 1 [file pone.0355844.s001.pdf]

S/N	Season	Block_Name	Months	Variety	Crop_age	Latitude	Longitude	Altitude	No_larva
1	Rain season	AP12A	2	R579	11.7	-1.213074097	31.26078461	High altitude	0
1	Dry season	AP12A	5	R579	14.9	-1.213074097	31.26078461	High altitude	2
2	Rain season	AS11A	2	N49	5.8	-1.205046597	31.26029322	High altitude	1
2	Dry season	AS11A	5	N49	9	-1.205046597	31.26029322	High altitude	3
3	Rain season	AP9D	2	N47	12.2	-1.216974653	31.25642294	High altitude	0
3	Dry season	AP9D	5	N47	15.4	-1.216974653	31.25642294	High altitude	0
4	Rain season	AP9A	2	N49	9.6	-1.217357986	31.25259572	High altitude	0
4	Dry season	AP9A	5	N49	12.8	-1.217357986	31.25259572	High altitude	1
5	Rain season	AS6D	2	N49	6.1	-1.218620486	31.24895461	High altitude	0
5	Dry season	AS6D	5	N49	9.4	-1.218620486	31.24895461	High altitude	4
6	Rain season	AP2D	2	R579	6.4	-1.224123264	31.24134017	High altitude	0
6	Dry season	AP2D	5	R579	9.6	-1.224123264	31.24134017	High altitude	0
7	Rain season	AP1A	2	R579	5.1	-1.218298819	31.23470461	High altitude	0
7	Dry season	AP1A	5	R579	8.3	-1.218298819	31.23470461	High altitude	4
8	Rain season	AP7D	2	N47	11.5	-1.230450486	31.25248822	High altitude	0
8	Dry season	AP7D	5	N47	14.7	-1.230450486	31.25248822	High altitude	2
9	Rain season	AP7C	2	R579	11.1	-1.234679653	31.25196017	High altitude	0
9	Dry season	AP7C	5	R579	14.3	-1.234679653	31.25196017	High altitude	0
10	Rain season	AP7B	2	N47	9.9	-1.233768264	31.247071	High altitude	0
10	Dry season	AP7B	5	N47	13.1	-1.233768264	31.247071	High altitude	1
11	Rain season	M5B	2	N47	11.3	-1.216203819	31.29231544	High altitude	1
11	Dry season	M5B	5	N47	14.5	-1.216203819	31.29231544	High altitude	1
12	Rain season	MS7D	2	N25	6.3	-1.213511597	31.287401	High altitude	0
12	Dry season	MS7D	5	N25	9.5	-1.213511597	31.287401	High altitude	2
13	Rain season	MS2A	2	N41	5.5	-1.219292431	31.28364878	High altitude	0
13	Dry season	MS2A	5	N41	8.7	-1.219292431	31.28364878	High altitude	2
14	Rain season	MS3B	2	R570	10.4	-1.219563542	31.28809267	High altitude	1
14	Dry season	MS3B	5	R570	13.6	-1.219563542	31.28809267	High altitude	2
15	Rain season	M1D	2	N47	12.2	-1.221876597	31.31336989	High altitude	0
15	Dry season	M1D	5	N47	15.4	-1.221876597	31.31336989	High altitude	1
16	Rain season	M1B	2	N47	9.8	-1.224794097	31.30489989	High altitude	1
16	Dry season	M1B	5	N47	13	-1.224794097	31.30489989	High altitude	1

17	Rain season	AP10C	2	N47	8.8	-1.230464653	31.26129211	High altitude	0
17	Dry season	AP10C	5	N47	12	-1.230464653	31.26129211	High altitude	0
18	Rain season	AS10F	2	R579	8.8	-1.236126319	31.26247878	High altitude	0
18	Dry season	AS10F	5	R579	12	-1.236126319	31.26247878	High altitude	0
19	Rain season	IP5B	2	N47	11.4	-1.222081938	31.17348805	High altitude	2
19	Dry season	IP5B	5	N47	14.6	-1.222081938	31.17348805	High altitude	3
20	Rain season	IP2C	2	N41	9.7	-1.22590127	31.17648822	High altitude	0
20	Dry season	IP2C	5	N41	12.9	-1.22590127	31.17648822	High altitude	1
21	Rain season	IP9B	2	N47	10.2	-1.23026288	31.31842157	High altitude	1
21	Dry season	IP9B	5	N47	13.4	-1.23026288	31.31842157	High altitude	2
22	Rain season	IS2C	2	N41	9.7	-1.226834325	31.17879267	High altitude	0
22	Dry season	IS2C	5	N41	12.9	-1.226834325	31.17879267	High altitude	1
23	Rain season	IS5B	2	N47	11.4	-1.233150936	31.28168852	High altitude	0
23	Dry season	IS5B	5	N47	14.6	-1.233150936	31.28168852	High altitude	1
24	Rain season	IP2D	2	N25	11	-1.243052986	31.1632185	High altitude	0
24	Dry season	IP2D	5	N25	14.2	-1.243052986	31.1632185	High altitude	0
25	Rain season	IP2B	2	N47	10.6	-1.243052986	31.1632185	High altitude	0
25	Dry season	IP2B	5	N47	13.8	-1.243052986	31.1632185	High altitude	0
26	Rain season	IS6A	2	N49	9.3	-1.236033158	31.32653768	High altitude	0
26	Dry season	IS6A	5	N49	12.5	-1.236033158	31.32653768	High altitude	0
27	Rain season	IS3E	2	N41	9.7	-1.215017493	31.1737436	High altitude	0
27	Dry season	IS3E	5	N41	12.9	-1.215017493	31.1737436	High altitude	0
28	Rain season	IR1G	2	N47	11.9	-1.233309317	31.36265656	High altitude	1
28	Dry season	IR1G	5	N47	15.1	-1.233309317	31.36265656	High altitude	3
29	Rain season	IS6D	2	CO617	11.9	-1.217466938	31.16002166	High altitude	1
29	Dry season	IS6D	5	CO617	15.1	-1.217466938	31.16002166	High altitude	1
30	Rain season	TP13C	2	R579	10.2	-1.216544375	31.37237378	High altitude	0
30	Dry season	TP13C	5	R579	13.4	-1.216544375	31.37237378	High altitude	0
31	Rain season	TP4A	2	R579	9.5	-1.222291042	31.28966072	High altitude	0
31	Dry season	TP4A	5	R579	12.7	-1.222291042	31.28966072	High altitude	0
32	Rain season	TP9B	2	N25	6.8	-1.231780208	31.36319072	High altitude	0
32	Dry season	TP9B	5	N25	10	-1.231780208	31.36319072	High altitude	1
33	Rain season	TP5B	2	N25	11.9	-1.215864931	31.372311	High altitude	0

33	Dry season	TP5B	5	N25	15.1	-1.215864931	31.372311	High altitude	1
34	Rain season	TR5B	2	N25	6.2	-1.227743542	31.33980406	High altitude	0
34	Dry season	TR5B	5	N25	9.4	-1.227743542	31.33980406	High altitude	5
35	Rain season	TR5C	2	N25	12.1	-1.216934097	31.35994906	High altitude	0
35	Dry season	TR5C	5	N25	15.3	-1.216934097	31.35994906	High altitude	0
36	Rain season	TP6D	2	N49	9.5	-1.226603819	31.34240656	High altitude	2
36	Dry season	TP6D	5	N49	12.7	-1.226603819	31.34240656	High altitude	2
37	Rain season	TP6B	2	N49	12.4	-1.223828264	31.33626489	High altitude	0
37	Dry season	TP6B	5	N49	15.6	-1.223828264	31.33626489	High altitude	0
38	Rain season	TS13D	2	R579	8.1	-1.225006769	31.2877599	High altitude	0
38	Dry season	TS13D	5	R579	11.3	-1.225006769	31.2877599	High altitude	6
39	Rain season	TS12D	2	N49	6.3	-1.229159097	31.34659406	High altitude	0
39	Dry season	TS12D	5	N49	15.7	-1.229159097	31.34659406	High altitude	0
40	Rain season	JP4C	2	N47	10	-1.25657604	31.24701546	High altitude	0
40	Dry season	JP4C	5	N47	13.2	-1.25657604	31.24701546	High altitude	3
41	Rain season	KS1F	2	N41	10.6	-1.219732606	31.35798623	High altitude	0
41	Dry season	KS1F	5	N41	13.9	-1.219732606	31.35798623	High altitude	6
42	Rain season	KP3D	2	N25	10.2	-1.238409547	31.31818907	High altitude	2
42	Dry season	KP3D	5	N25	13.5	-1.238409547	31.31818907	High altitude	0
43	Rain season	KS6A	2	N41	10.4	-1.186984037	31.25815117	High altitude	0
43	Dry season	KS6A	5	N41	13.7	-1.186984037	31.25815117	High altitude	0
44	Rain season	KS6C	2	Mixed	10.6	-1.227144375	31.35084461	High altitude	1
44	Dry season	KS6C	5	Mixed	13.9	-1.227144375	31.35084461	High altitude	1
45	Rain season	KS4B	2	N46	6.2	-1.241347051	31.35478262	High altitude	0
45	Dry season	KS4B	5	N46	9.5	-1.241347051	31.35478262	High altitude	4
46	Rain season	FP4D	2	R579	10	-1.232432198	31.35157606	High altitude	0
46	Dry season	FP4D	5	R579	13.3	-1.232432198	31.35157606	High altitude	1
47	Rain season	FS25A	2	N41	10.9	-1.217566921	31.36876856	High altitude	0
47	Dry season	FS25A	5	N41	14.2	-1.217566921	31.36876856	High altitude	6
48	Rain season	FR12B	2	N47	7.7	-1.228854421	31.370458	High altitude	0
48	Dry season	FR12B	5	N47	11	-1.228854421	31.370458	High altitude	3
49	Rain season	FP10B	2	N41	12.7	-1.226435532	31.33685439	High altitude	0
49	Dry season	FP10B	5	N41	15.9	-1.226435532	31.33685439	High altitude	0

50	Rain season	FP5B	2	N25	9.6	-1.232520254	31.35497661	High altitude	0
50	Dry season	FP5B	5	N25	12.9	-1.232520254	31.35497661	High altitude	5
51	Rain season	FP4B	2	N41	10.5	-1.222618032	31.37484911	High altitude	0
51	Dry season	FP4B	5	N41	13.8	-1.222618032	31.37484911	High altitude	4
52	Rain season	FP5C	2	N25	7.4	-1.228055809	31.35618522	High altitude	0
52	Dry season	FP5C	5	N25	10.7	-1.228055809	31.35618522	High altitude	4
53	Rain season	FS3C	2	N49	5.8	-1.220353587	31.37305661	High altitude	0
53	Dry season	FS3C	5	N49	9.1	-1.220353587	31.37305661	High altitude	11
54	Rain season	FR1B	2	N49	6.5	-1.206969698	31.35534606	High altitude	0
54	Dry season	FR1B	5	N49	9.8	-1.206969698	31.35534606	High altitude	8
55	Rain season	FP11D	2	N49	11.4	-1.200384653	31.34603544	High altitude	1
55	Dry season	FP11D	5	N49	14.6	-1.200384653	31.34603544	High altitude	1
56	Rain season	FR3D	2	N25	6.8	-1.207238865	31.35949133	High altitude	0
56	Dry season	FR3D	5	N25	10.1	-1.207238865	31.35949133	High altitude	4
57	Rain season	LP5A	2	CO617	11.3	-1.224720102	31.27569268	High altitude	0
57	Dry season	LP5A	5	CO617	14.5	-1.224720102	31.27569268	High altitude	0
58	Rain season	JP6C	2	N41	10.2	-1.251108262	31.26805852	High altitude	0
58	Dry season	JP6C	5	N41	13.5	-1.251108262	31.26805852	High altitude	1
59	Rain season	JP5B	2	N41	11.4	-1.219268717	31.14788082	High altitude	0
59	Dry season	JP5B	5	N41	14.6	-1.254557706	31.23806379	High altitude	0
60	Rain season	JP7C	2	N41	18	-1.23337538	31.33108685	High altitude	0
60	Dry season	JP7C	5	N41	3.2	-1.23337538	31.33108685	High altitude	0
61	Rain season	JP7C	2	MN1	11	-1.231108713	31.31005324	High altitude	0
61	Dry season	JP7C	5	MN1	14.2	-1.231108713	31.31005324	High altitude	0
62	Rain season	M12B	2	N41	11.5	-1.227479653	31.31068433	High altitude	6
62	Dry season	M12B	5	N41	14.7	-1.227479653	31.31068433	High altitude	6
63	Rain season	EP10B	2	N46	11.1	-1.224723032	31.32703356	High altitude	2
63	Dry season	EP10B	5	N46	14.3	-1.224723032	31.32703356	High altitude	2
64	Rain season	M12E	2	N41	11.8	-1.224723032	31.32703356	High altitude	1
64	Dry season	M12E	5	N41	15	-1.224723032	31.32703356	High altitude	0
65	Rain season	M12C	2	N41	11.7	-1.224723032	31.32703356	High altitude	0
65	Dry season	M12C	5	N41	14.9	-1.224723032	31.32703356	High altitude	0
66	Rain season	EP10A	2	N46	10	-1.201810486	31.32529044	High altitude	1

66	Dry season	EP10A	5	N46	10	-1.201810486	31.32529044	High altitude	2
67	Rain season	EP5C	2	N49	9.2	-1.214707153	31.32516156	High altitude	1
67	Dry season	EP5C	5	N49	12.5	-1.214707153	31.32516156	High altitude	3
68	Rain season	M12A	2	CO617	14.2	-1.227711319	31.326236	High altitude	6
68	Dry season	M12A	5	CO617	17.4	-1.227711319	31.326236	High altitude	6
69	Rain season	ES5B	2	N25	9.7	-1.219113542	31.31049017	High altitude	0
69	Dry season	ES5B	5	N25	13	-1.219113542	31.31049017	High altitude	0
70	Rain season	EP9A	2	R579	10.1	-1.229241643	31.34100494	High altitude	2
70	Dry season	EP9A	5	R579	13.4	-1.229241643	31.34100494	High altitude	3
71	Rain season	IP7A	2	N47	11.1	-1.22355916	31.15515166	High altitude	1
71	Dry season	IP7A	5	N47	14.3	-1.22355916	31.15515166	High altitude	0
72	Rain season	IP7D	2	N47	11.2	-1.231108713	31.31005324	High altitude	1
72	Dry season	IP7D	5	N47	14.4	-1.231108713	31.31005324	High altitude	3
73	Rain season	LP1B	2	N49	9.8	-1.330498973	31.24430235	High altitude	1
73	Dry season	LP1B	5	N49	13.3	-1.330498973	31.24430235	High altitude	10
74	Rain season	LP2D	2	N47	10.1	-1.268997324	31.3406999	High altitude	1
74	Dry season	LP2D	5	N47	13.3	-1.268997324	31.3406999	High altitude	3
75	Rain season	LP2C	2	N41	10	-1.246230936	31.35400713	High altitude	1
75	Dry season	LP2C	5	N41	13.3	-1.246230936	31.35400713	High altitude	9
76	Rain season	LP7C	2	N41	11.7	-1.249756491	31.39298907	High altitude	0
76	Dry season	LP7C	5	N41	14.9	-1.249756491	31.39298907	High altitude	0
77	Rain season	LP7D	2	N41	11.6	-1.259159097	31.35659406	High altitude	0
77	Dry season	LP7D	5	N41	14.8	-1.259159097	31.35659406	High altitude	0
78	Rain season	LP1A	2	N49	10.2	-1.22716094	31.36281707	High altitude	0
78	Dry season	LP1A	5	N49	13.4	-1.22716094	31.36281707	High altitude	2
79	Rain season	DP5C	2	N41	14.3	-1.210097986	31.3059485	High altitude	4
79	Dry season	DP5C	5	N41	17.5	-1.210097986	31.3059485	High altitude	6
80	Rain season	AP5A	2	N47	9.1	-1.212500486	31.24423739	High altitude	1
80	Dry season	AP5A	5	N47	12.4	-1.212500486	31.24423739	High altitude	2
81	Rain season	DR3D	2	N41	10.3	-1.203174931	31.29435239	High altitude	0
81	Dry season	DR3D	5	N41	13.5	-1.203174931	31.29435239	High altitude	0
82	Rain season	BP5D	2	N25	12.2	-1.200779375	31.28588072	High altitude	1
82	Dry season	BP5D	5	N25	15.4	-1.200779375	31.28588072	High altitude	3

83	Rain season	CP3A	2	Mixed	6.4	-1.229537754	31.29290994	High altitude		0
83	Dry season	CP3A	5	Mixed	9.7	-1.229537754	31.29290994	High altitude		2
84	Rain season	CS2B	2	N41	6.4	-1.226622198	31.29279106	High altitude		0
84	Dry season	CS2B	5	N41	9.7	-1.226622198	31.29279106	High altitude		2
85	Rain season	BP6C	2	N25	10.2	-1.188024097	31.27836906	High altitude		3
85	Dry season	BP6C	5	N25	13.4	-1.188024097	31.27836906	High altitude		4
86	Rain season	BS6B	2	N41	11.6	-1.193072986	31.27304267	High altitude		0
86	Dry season	BS6B	5	N41	14.8	-1.193072986	31.27304267	High altitude		0
87	Rain season	BR3B	2	N47	5.5	-1.202973264	31.27269711	High altitude		0
87	Dry season	BR3B	5	N47	8.8	-1.202973264	31.27269711	High altitude		2
88	Rain season	BP2D	2	N49	10.7	-1.200167986	31.26959322	High altitude		7
88	Dry season	BP2D	5	N49	13.9	-1.200167986	31.26959322	High altitude		9
89	Rain season	LP11C	2	N47	11.6	-1.237225384	31.36021707	High altitude		0
89	Dry season	LP11C	5	N47	14.8	-1.237225384	31.36021707	High altitude		0
90	Rain season	LP9A	2	N41	9.2	-1.29324854	31.29742685	High altitude		0
90	Dry season	LP9A	5	N41	12.5	-1.29324854	31.29742685	High altitude		13
91	Rain season	LP8D	2	N41	9.7	-1.28324854	31.24742685	High altitude		0
91	Dry season	LP8D	5	N41	13	-1.28324854	31.24742685	High altitude		0
92	Rain season	LP7A	2	N41	9.3	-1.267122476	31.10468194	High altitude		0
92	Dry season	LP7A	6	N41	12.6	-1.267122476	31.10468194	High altitude		1
93	Rain season	LP6A	2	N41	11.3	-1.247787431	31.11475667	High altitude		1
93	Dry season	LP6A	6	N41	14.6	-1.247787431	31.11475667	High altitude		2
94	Rain season	LP6C	2	N41	11.6	-1.174034002	31.065857	High altitude		0
94	Dry season	LP6C	6	N41	14.9	-1.174034002	31.065857	High altitude		0
95	Rain season	NP4A	2	N49	10.6	-1.184034002	31.075857	High altitude		0
95	Dry season	NP4A	6	N49	13.9	-1.184034002	31.075857	High altitude		0
96	Rain season	NP1B	2	N49	10	-1.175146063	31.033706	High altitude		0
96	Dry season	NP1B	6	N49	13.5	-1.175146063	31.033706	High altitude		9
97	Rain season	NP4C	2	N49	10.5	-1.190343447	31.07513339	High altitude		0
97	Dry season	NP4C	6	N49	13.8	-1.190343447	31.07513339	High altitude		0
98	Rain season	JP8A	2	N47	6.6	-1.171398724	31.0895445	High altitude		1
98	Dry season	JP8A	6	N47	10.1	-1.171398724	31.0895445	High altitude		2
99	Rain season	NP6D	2	N25	12.4	-1.181398724	31.0695445	High altitude		0

99	Dry season	NP6D	6	N25	15.7	-1.181398724	31.0695445	High altitude	2
100	Rain season	NP5B	2	N49	10.5	-1.174860113	31.06827283	High altitude	1
100	Dry season	NP5B	6	N49	13.8	-1.174860113	31.06827283	High altitude	3
101	Rain season	NP6B	2	N47	6.8	-1.188747277	31.07839677	High altitude	0
101	Dry season	NP6B	6	N47	10.3	-1.188747277	31.07839677	High altitude	5
102	Rain season	NP13D	2	N49	12.2	-1.186182335	31.04812089	High altitude	0
102	Dry season	NP13D	6	N49	15.5	-1.186182335	31.04812089	High altitude	1
103	Rain season	NP13C	2	N49	12.2	-1.221429931	31.28265794	High altitude	0
103	Dry season	NP13C	6	N49	15.5	-1.221429931	31.28265794	High altitude	0
104	Rain season	NP15C	2	N41	7.3	-1.184305507	31.03884906	High altitude	1
104	Dry season	NP15C	6	N41	10.8	-1.184305507	31.03884906	High altitude	6
105	Rain season	NP9B	2	N25	9.5	-1.193517335	31.06928839	High altitude	0
105	Dry season	NP9B	6	N25	13	-1.193517335	31.06928839	High altitude	3
106	Rain season	EP11D	2	N49	9.3	-1.223713587	31.33853994	High altitude	0
106	Dry season	EP11D	6	N49	12.8	-1.223713587	31.33853994	High altitude	13
107	Rain season	EP11A	2	N25	6.3	-1.222198309	31.32630106	High altitude	0
107	Dry season	EP11A	6	N25	9.8	-1.222198309	31.32630106	High altitude	0
108	Rain season	NS3E	2	N49	9.8	-1.181599558	31.06031283	High altitude	0
108	Dry season	NS3E	6	N49	13.3	-1.181599558	31.06031283	High altitude	5
109	Rain season	DR8C	2	N41	10.3	-1.226573865	31.31742689	High altitude	0
109	Dry season	DR8C	6	N41	10.3	-1.226573865	31.31742689	High altitude	8
110	Rain season	DR7A	2	N41	9.6	-1.213260254	31.31191494	High altitude	1
110	Dry season	DR7A	6	N41	16.4	-1.213260254	31.31191494	High altitude	2
111	Rain season	EP11C	2	N25	9.3	-1.221643865	31.33822772	High altitude	2
111	Dry season	EP11C	6	N25	12.8	-1.221643865	31.33822772	High altitude	2
112	Rain season	EP11B	2	N25	5.1	-1.224151643	31.32529217	High altitude	1
112	Dry season	EP11B	6	N25	8.6	-1.224151643	31.32529217	High altitude	4
113	Rain season	DP8A	2	N25	10.2	-1.220173309	31.31039994	High altitude	2
113	Dry season	DP8A	6	N25	16.8	-1.220173309	31.31039994	High altitude	4
114	Rain season	DP9AB	2	N47	9.6	-1.214371921	31.31862856	High altitude	0
114	Dry season	DP9AB	6	N47	13.1	-1.214371921	31.31862856	High altitude	4
115	Rain season	DR6A	2	N41	5.6	-1.204352431	31.31012961	High altitude	2
115	Dry season	DR6A	6	N41	9.1	-1.204352431	31.31012961	High altitude	8

116	Rain season	DS10C	2	N41	5.8	-1.227122476	31.30826272	High altitude	0
116	Dry season	DS10C	6	N41	9.3	-1.227122476	31.30826272	High altitude	2
117	Rain season	GP9A	2	N49	10.7	-1.237787431	31.23877767	High altitude	0
117	Dry season	GP9A	6	N49	14.2	-1.237787431	31.23877767	High altitude	12
118	Rain season	GP9B	2	N49	12.2	-1.235441875	31.24299544	High altitude	0
118	Dry season	GP9B	6	N49	15.5	-1.235441875	31.24299544	High altitude	2
119	Rain season	GP6C	2	N49	10.9	-1.237437986	31.22165239	High altitude	2
119	Dry season	GP6C	6	N49	14.2	-1.237437986	31.22165239	High altitude	4
120	Rain season	GS3D	2	R570	10.1	-1.230687153	31.24329878	High altitude	1
120	Dry season	GS3D	6	R570	13.6	-1.230687153	31.24329878	High altitude	3
121	Rain season	GP8D	2	R579	9.5	-1.229045764	31.23483961	High altitude	1
121	Dry season	GP8D	6	R579	13	-1.229045764	31.23483961	High altitude	2
122	Rain season	GP8B	2	R579	9.1	-1.228793819	31.23060628	High altitude	0
122	Dry season	GP8B	6	R579	12.6	-1.228793819	31.23060628	High altitude	2
123	Rain season	GP5A	2	N25	8	-1.230359325	31.24329433	High altitude	1
123	Dry season	GP5A	6	N25	14.3	-1.230359325	31.24329433	High altitude	6
124	Rain season	GS7A	2	N47	8.6	-1.245422431	31.24471517	High altitude	0
124	Dry season	GS7A	6	N47	12.1	-1.245422431	31.24471517	High altitude	2
125	Rain season	GP7A	2	N47	10	-1.238667708	31.22971072	High altitude	0
125	Dry season	GP7A	6	N47	13.5	-1.238667708	31.22971072	High altitude	1
126	Rain season	GP7D	2	N47	9.2	-1.247886597	31.24084517	High altitude	1
126	Dry season	GP7D	6	N47	12.5	-1.247886597	31.24084517	High altitude	6
127	Rain season	GS4B	2	Mixed	11.8	-1.235769375	31.21055906	High altitude	1
127	Dry season	GS4B	6	Mixed	15.1	-1.235769375	31.21055906	High altitude	6
128	Rain season	GP4B	2	R579	12.6	-1.226437103	31.23389739	High altitude	0
128	Dry season	GP4B	6	R579	15.9	-1.226437103	31.23389739	High altitude	4
129	Rain season	GS702	2	N47	11.7	-1.239870764	31.22081239	High altitude	0
129	Dry season	GS702	6	N47	15.2	-1.239870764	31.22081239	High altitude	2
130	Rain season	GS1A	2	N49	8.9	-1.225111319	31.23505517	High altitude	1
130	Dry season	GS1A	6	N49	12.4	-1.225111319	31.23505517	High altitude	9
131	Rain season	HS4E	2	N47	12	-1.240679653	31.18553878	High altitude	0
131	Dry season	HS4E	6	N47	15.5	-1.240679653	31.18553878	High altitude	0
132	Rain season	HP4D	2	N41	8.1	-1.231370486	31.24432017	High altitude	0

132	Dry season	HP4D	6	N41	11.6	-1.231370486	31.24432017	High altitude	0
133	Rain season	HP4C	2	N41	10.7	-1.235854375	31.23502017	High altitude	1
133	Dry season	HP4C	6	N41	14.2	-1.235854375	31.23502017	High altitude	6
134	Rain season	HS5A	2	N47	7.9	-1.225034881	31.19948461	High altitude	0
134	Dry season	HS5A	6	N47	14.9	-1.225034881	31.19948461	High altitude	9
135	Rain season	HR6C	2	N47	16.5	-1.224151825	31.2193685	High altitude	2
135	Dry season	HR6C	6	N47	20	-1.224151825	31.2193685	High altitude	6
136	Rain season	GP2B	2	N47	12.2	-1.208704931	31.37425017	High altitude	0
136	Dry season	GP2B	6	N47	15.7	-1.208704931	31.37425017	High altitude	3
137	Rain season	GS8D	2	N41	14.1	-1.234005486	31.20571017	High altitude	1
137	Dry season	GS8D	6	N41	3.5	-1.234005486	31.20571017	High altitude	1
138	Rain season	OP1C	2	N41	8.3	-1.290885484	31.27715046	High altitude	0
138	Dry season	OP1C	6	N41	11.8	-1.290885484	31.27715046	High altitude	2
139	Rain season	OS2G	2	N25	12.1	-1.280885484	31.26715046	High altitude	0
139	Dry season	OS2G	6	N25	15.6	-1.280885484	31.26715046	High altitude	4
140	Rain season	OS5D	2	N25	10.8	-1.271704373	31.2813449	High altitude	8
140	Dry season	OS5D	6	N25	14.3	-1.271704373	31.2813449	High altitude	8
141	Rain season	OS5C	2	N25	9.1	-1.277747151	31.28685157	High altitude	2
141	Dry season	OS5C	6	N25	12.6	-1.277747151	31.28685157	High altitude	4
142	Rain season	OP5D	2	N25	12.1	-1.333167584	31.26586763	High altitude	0
142	Dry season	OP5D	6	N25	15.6	-1.333167584	31.26586763	High altitude	3
143	Rain season	1P7A	2	R579	6.9	-1.196816938	31.1592811	High altitude	5
143	Dry season	1P7A	6	R579	13.9	-1.196816938	31.1592811	High altitude	9
144	Rain season	ER2A	2	N49	13.6	-1.232293032	31.34626606	High altitude	1
144	Dry season	ER2A	6	N49	17.1	-1.232293032	31.34626606	High altitude	4
145	Rain season	ES2B	2	N25	13.6	-1.203740764	31.32481711	High altitude	1
145	Dry season	ES2B	6	N25	3.5	-1.203740764	31.32481711	High altitude	1
146	Rain season	ER3D	2	N46	10	-1.214019653	31.31745822	High altitude	2
146	Dry season	ER3D	6	N46	13.5	-1.214019653	31.31745822	High altitude	2
147	Rain season	7P7B	2	N46	8.6	-1.259522509	31.25033612	High altitude	1
147	Dry season	7P7B	6	N46	12.1	-1.259522509	31.25033612	High altitude	3
148	Rain season	7P8C	2	N47	13.6	-1.257753342	31.25517445	High altitude	3
148	Dry season	7P8C	6	N47	3.5	-1.257753342	31.25517445	High altitude	0

149	Rain season	7P9A	2	N49	11.9	-1.264088898	31.26557473	High altitude	8
149	Dry season	7P9A	6	N49	15.4	-1.264088898	31.26557473	High altitude	8
150	Rain season	7P8D	2	N47	13.3	-1.259915286	31.25578529	High altitude	2
150	Dry season	7P8D	6	N47	16.8	-1.259915286	31.25578529	High altitude	4
151	Rain season	7P19A	2	N41	13.6	-1.24048112	31.26672834	High altitude	1
151	Dry season	7P19A	6	N41	17.1	-1.24048112	31.26672834	High altitude	3
152	Rain season	7P19D	2	N41	9.4	-1.240355842	31.26499862	High altitude	8
152	Dry season	7P19D	6	N41	12.9	-1.240355842	31.26499862	High altitude	8
153	Rain season	10R5D	2	N41	8.8	-1.218551398	31.16006029	High altitude	9
153	Dry season	10R5D	6	N41	12.3	-1.218551398	31.16006029	High altitude	13
154	Rain season	11P19A	2	N49	9.5	-1.205049898	31.2920229	High altitude	23
154	Dry season	11P19A	6	N49	13	-1.205049898	31.2920229	High altitude	3
155	Rain season	10R5A	2	N49	9.2	-1.216215564	31.16624279	High altitude	10
155	Dry season	10R5A	6	N49	12.7	-1.216215564	31.16624279	High altitude	5
156	Rain season	11R13B	2	N49	16.4	-1.196872786	31.22101168	High altitude	9
156	Dry season	11R13B	6	N49	16.4	-1.196872786	31.22101168	High altitude	9
157	Rain season	3P4A	2	N41	13	-1.216150764	31.19272572	High altitude	8
157	Dry season	3P4A	6	N41	16.5	-1.216150764	31.19272572	High altitude	10
158	Rain season	3P4C	2	N41	10.9	-1.215834653	31.18881711	High altitude	9
158	Dry season	3P4C	6	N41	3.5	-1.215834653	31.18881711	High altitude	2
159	Rain season	7R2A	2	N41	11.1	-1.181636871	31.20716556	High altitude	4
159	Dry season	7R2A	6	N41	14.6	-1.181636871	31.20716556	High altitude	6
160	Rain season	6P7D	2	N41	11.3	-1.19352737	31.24315033	High altitude	0
160	Dry season	6P7D	6	N41	14.8	-1.19352737	31.24315033	High altitude	4
161	Rain season	5P5C	2	N41	11.6	-1.203769931	31.21562433	High altitude	1
161	Dry season	5P5C	6	N41	15.1	-1.203769931	31.21562433	High altitude	2
162	Rain season	3R14A	2	N47	17.7	-1.197514375	31.21290156	High altitude	1
162	Dry season	3R14A	6	N47	3.6	-1.197514375	31.21290156	High altitude	1
163	Rain season	5P5B	2	N41	12.4	-1.204582986	31.21872767	High altitude	1
163	Dry season	5P5B	6	N41	15.9	-1.204582986	31.21872767	High altitude	2
164	Rain season	5R10B	2	N41	13.3	-1.189627153	31.22997739	High altitude	1
164	Dry season	5R10B	6	N41	16.8	-1.189627153	31.22997739	High altitude	1
165	Rain season	7R3A	2	N49	13.9	-1.278516953	31.25729751	High altitude	12

165	Dry season	7R3A	6	N49	17.4	-1.278516953	31.25729751	High altitude	14
166	Rain season	6R11B	2	N49	13.3	-1.170573335	31.39402455	High altitude	5
166	Dry season	6R11B	6	N49	16.8	-1.170573335	31.39402455	High altitude	7
167	Rain season	1P8C	2	N41	8.9	-1.192765826	31.16400527	High altitude	5
167	Dry season	1P8C	6	N41	12.4	-1.192765826	31.16400527	High altitude	7
168	Rain season	1P6C	2	N41	12.2	-1.20064666	31.1621336	High altitude	9
168	Dry season	1P6C	6	N41	15.7	-1.20064666	31.1621336	High altitude	12
169	Rain season	IR4A	2	N47	12.5	-1.203472493	31.15146221	High altitude	4
169	Dry season	IR4A	6	N47	16	-1.203472493	31.15146221	High altitude	8
170	Rain season	1P6D	2	N41	12.6	-1.203700549	31.16225888	High altitude	7
170	Dry season	1P6D	6	N41	16.1	-1.203700549	31.16225888	High altitude	10
171	Rain season	2P12A	2	N47	13.5	-1.204814375	31.15579378	High altitude	4
171	Dry season	2P12A	6	N47	17	-1.204814375	31.15579378	High altitude	4
172	Rain season	2R11D	2	N49	6.1	-1.219960953	31.37280709	High altitude	4
172	Dry season	2R11D	6	N49	9.6	-1.219960953	31.37280709	High altitude	9
173	Rain season	1P7D	2	R579	7.6	-1.19864916	31.1566211	High altitude	4
173	Dry season	1P7D	6	R579	11.1	-1.19864916	31.1566211	High altitude	8
174	Rain season	2P17D	2	N41	12.4	-1.195870764	31.17715822	High altitude	13
174	Dry season	2P17D	6	N41	15.9	-1.195870764	31.17715822	High altitude	16
175	Rain season	2P17A	2	N41	12.3	-1.194425486	31.18027378	High altitude	8
175	Dry season	2P17A	6	N41	15.8	-1.194425486	31.18027378	High altitude	10
176	Rain season	2P13D	2	N41	9.3	-1.202717431	31.16101267	High altitude	12
176	Dry season	2P13D	6	N41	12.8	-1.202717431	31.16101267	High altitude	12
177	Rain season	2P18A	2	Mixed	10.7	-1.207153542	31.1740135	High altitude	11
177	Dry season	2P18A	6	Mixed	14.2	-1.207153542	31.1740135	High altitude	13
178	Rain season	2P11D	2	N47	13.1	-1.206032986	31.17550711	High altitude	9
178	Dry season	2P11D	6	N47	18.6	-1.206032986	31.17550711	High altitude	11
179	Rain season	2P11A	2	N47	10.8	-1.205643542	31.17848406	High altitude	5
179	Dry season	2P11A	6	N47	14.3	-1.205643542	31.17848406	High altitude	7
180	Rain season	2R8A	2	R579	10.8	-1.196704438	31.16858055	High altitude	0
180	Dry season	2R8A	6	R579	3.5	-1.196704438	31.16858055	High altitude	0
181	Rain season	2R5B	2	R579	10.8	-1.216697986	31.16218378	High altitude	1
181	Dry season	2R5B	6	R579	14.3	-1.216697986	31.16218378	High altitude	6

182	Rain season	2R11A	2	N49	10.7	-1.207149375	31.18020961	High altitude	0
182	Dry season	2R11A	6	N49	14.2	-1.207149375	31.18020961	High altitude	2
183	Rain season	3P15R	2	N41	9.3	-1.192243542	31.20384211	High altitude	5
183	Dry season	3P15R	6	N41	12.8	-1.192243542	31.20384211	High altitude	7
184	Rain season	1P5B	2	N41	8.2	-1.215831319	31.12052656	High altitude	0
184	Dry season	1P5B	6	N41	11.7	-1.215831319	31.12052656	High altitude	3
185	Rain season	5P7D	2	N41	7.9	-1.205957431	31.23075156	High altitude	4
185	Dry season	5P7D	6	N41	11.4	-1.205957431	31.23075156	High altitude	5
186	Rain season	3R1C	2	N41	10.8	-1.224490764	31.17862239	High altitude	10
186	Dry season	3R1C	6	N41	14.5	-1.224490764	31.17862239	High altitude	7
187	Rain season	5P7C	2	N49	7.7	-1.203866319	31.23064433	High altitude	1
187	Dry season	5P7C	6	N49	11.2	-1.203866319	31.23064433	High altitude	2
188	Rain season	3P1D	2	N41	13.4	-1.229380486	31.18200211	High altitude	12
188	Dry season	3P1D	6	N41	13.4	-1.229380486	31.18200211	High altitude	14
189	Rain season	1P16D	2	N47	9	-1.204764375	31.13739572	High altitude	6
189	Dry season	1P16D	6	N47	4	-1.204764375	31.13739572	High altitude	0
190	Rain season	1P16B	2	N47	9.1	-1.200726597	31.13930406	High altitude	0
190	Dry season	1P16B	6	N47	12.6	-1.200726597	31.13930406	High altitude	3
191	Rain season	3R3C	2	N41	10.8	-1.216360486	31.17986433	High altitude	6
191	Dry season	3R3C	6	N41	14.1	-1.216360486	31.17986433	High altitude	10
192	Rain season	1P17A	2	Mixed	9.2	-1.200376875	31.14808572	High altitude	2
192	Dry season	1P17A	6	Mixed	12.7	-1.200376875	31.14808572	High altitude	8
193	Rain season	1P17B	2	N47	9.2	-1.197412986	31.14672072	High altitude	0
193	Dry season	1P17B	6	N47	12.7	-1.197412986	31.14672072	High altitude	3
194	Rain season	3P3D	2	N41	11.1	-1.221042153	31.18223656	High altitude	7
194	Dry season	3P3D	6	N41	14.6	-1.221042153	31.18223656	High altitude	16
195	Rain season	1P3B	3	N41	7.7	-1.204935271	31.14695138	High altitude	5
195	Dry season	1P3B	6	N41	11.2	-1.204935271	31.14695138	High altitude	9
196	Rain season	GP3B	3	N41	9.2	-1.241246319	31.21283794	High altitude	1
196	Dry season	GP3B	6	N41	12.7	-1.241246319	31.21283794	High altitude	2
197	Rain season	7R10B	3	N49	7	-1.196123898	31.26743084	High altitude	4
197	Dry season	7R10B	6	N49	10.5	-1.196123898	31.26743084	High altitude	9
198	Rain season	11P19B	3	N49	9.4	-1.202927953	31.29110068	High altitude	12

198	Dry season	11P19B	6	N49	12.9	-1.202927953	31.29110068	High altitude	16
199	Rain season	11P18A	3	N49	11.2	-1.203714453	31.2788664	High altitude	5
199	Dry season	11P18A	6	N49	14.6	-1.203714453	31.2788664	High altitude	9
200	Rain season	10P1D	3	Mixed	7.5	-1.224205564	31.1607789	High altitude	2
200	Dry season	10P1D	6	Mixed	11.2	-1.224205564	31.1607789	High altitude	7
201	Rain season	HP7B	3	N47	11.4	-1.226562103	31.22000933	High altitude	0
201	Dry season	HP7B	6	N47	14.7	-1.226562103	31.22000933	High altitude	2
202	Rain season	HS7C	3	R579	16	-1.227931548	31.22515461	High altitude	0
202	Dry season	HS7C	6	R579	19	-1.227931548	31.22515461	High altitude	0
203	Rain season	OP6B	3	N49	6.3	-1.193137648	31.13970278	High altitude	1
203	Dry season	OP6B	6	N49	9.3	-1.225660714	31.13970278	High altitude	2
204	Rain season	HS2B	3	N25	8.8	-1.230805159	31.20760072	High altitude	0
204	Dry season	HS2B	6	N25	11.8	-1.230805159	31.20760072	High altitude	0
205	Rain season	7P5C	3	R579	8.8	-1.269052786	31.24550112	High altitude	0
205	Dry season	7P5C	6	R579	11.8	-1.269052786	31.24550112	High altitude	3
206	Rain season	7R8D	3	N41	9.8	-1.262043898	31.25439584	High altitude	1
206	Dry season	7R8D	6	N41	12.8	-1.262043898	31.25439584	High altitude	3
207	Rain season	SR18D	3	N49	10.8	-1.192481597	31.16899072	High altitude	9
207	Dry season	SR18D	6	N49	13.8	-1.192481597	31.16899072	High altitude	13
208	Rain season	1P3B	3	N41	7.7	-1.204935271	31.14695138	High altitude	2
208	Dry season	1P3B	6	N41	10.7	-1.204935271	31.14695138	High altitude	4
209	Rain season	DR5D	3	N41	7.6	-1.204927708	31.30871989	High altitude	0
209	Dry season	DR5D	6	N41	10.6	-1.204927708	31.30871989	High altitude	1
210	Rain season	DS10D	3	N47	12.6	-1.226573865	31.31742689	High altitude	0
210	Dry season	DS10D	6	N47	12.6	-1.226573865	31.31742689	High altitude	2
211	Rain season	BS7D	3	N25	7	-1.188140208	31.28777711	High altitude	0
211	Dry season	BS7D	6	N25	10	-1.188140208	31.28777711	High altitude	3
212	Rain season	AS13B	3	N47	12.5	-1.230410764	31.26420239	High altitude	0
212	Dry season	AS13B	6	N47	15.5	-1.230410764	31.26420239	High altitude	3
213	Rain season	2P17B	3	R579	12	-1.192208819	31.17862656	High altitude	11
213	Dry season	2P17B	6	R579	15	-1.192208819	31.17862656	High altitude	12
214	Rain season	3P11A	3	N41	8.9	-1.198336597	31.19610017	High altitude	2
214	Dry season	3P11A	6	N41	11.9	-1.198336597	31.19610017	High altitude	9

215	Rain season	1P3C	3	N41	7.7	-1.206251104	31.14540443	High altitude	0
215	Dry season	1P3C	6	N41	10.7	-1.206251104	31.14540443	High altitude	6
216	Rain season	1P5C	3	N49	8.3	-1.203659715	31.15359916	High altitude	7
216	Dry season	1P5C	6	N49	11.3	-1.203659715	31.15359916	High altitude	12
217	Rain season	6P8B	3	N49	8.1	-1.18630737	31.253427	High altitude	1
217	Dry season	6P8B	6	N49	14.3	-1.18630737	31.253427	High altitude	3
218	Rain season	6P7C	3	Mixed	8.9	-1.190549592	31.24182728	High altitude	1
218	Dry season	6P7C	6	Mixed	11.9	-1.190549592	31.24182728	High altitude	5
219	Rain season	TP9D	3	N25	9	-1.219624653	31.33124628	High altitude	1
219	Dry season	TP9D	6	N25	12	-1.219624653	31.33124628	High altitude	9
220	Rain season	TP9C	3	N25	9.1	-1.215927708	31.3304735	High altitude	0
220	Dry season	TP9C	6	N25	12.1	-1.215927708	31.3304735	High altitude	6
221	Rain season	TP9A	3	N49	6.8	-1.212733819	31.36870517	High altitude	0
221	Dry season	TP9A	6	N49	9.8	-1.212733819	31.36870517	High altitude	2
222	Rain season	TR7A	3	N47	9.3	-1.219128819	31.33558933	High altitude	0
222	Dry season	TR7A	6	N47	12.3	-1.219128819	31.33558933	High altitude	4
223	Rain season	ES1B	3	N41	8.4	-1.213426319	31.32680767	High altitude	0
223	Dry season	ES1B	6	N41	11.4	-1.213426319	31.32680767	High altitude	1
224	Rain season	TP9B	3	Mixed	8.8	-1.231780208	31.36319072	High altitude	0
224	Dry season	TP9B	6	Mixed	11.8	-1.231780208	31.36319072	High altitude	3
225	Rain season	6P11B	3	N49	12.2	-1.217625321	31.36527056	High altitude	10
225	Dry season	6P11B	6	N49	15.2	-1.217625321	31.36527056	High altitude	14
226	Rain season	6P11C	3	N41	7.1	-1.185015426	31.23465506	High altitude	1
226	Dry season	6P11C	6	N41	10.1	-1.185015426	31.23465506	High altitude	4
227	Rain season	TP2A	3	N47	7.6	-1.213622153	31.35895767	High altitude	0
227	Dry season	TP2A	6	N47	10.6	-1.213622153	31.35895767	High altitude	0
228	Rain season	LP5D	3	CO617	13.3	-1.250932101	31.23679685	High altitude	1
228	Dry season	LP5D	6	CO617	16.3	-1.250932101	31.23679685	High altitude	4
229	Rain season	NP18B	3	N47	9.2	-1.188186006	31.05540672	High altitude	0
229	Dry season	NP18B	6	N47	12.2	-1.188186006	31.05540672	High altitude	7
230	Rain season	IS10D	3	N49	8.2	-1.240690764	31.15575294	High altitude	0
230	Dry season	IS10D	6	N49	11.2	-1.240690764	31.15575294	High altitude	6
231	Rain season	JP6C	3	N41	12.2	-1.236987935	31.24437657	High altitude	0

231	Dry season	JP6C	6	N41	15.2	-1.236987935	31.24437657	High altitude	2
232	Rain season	IP10D	3	N49	7.9	-1.233181548	31.19925211	High altitude	0
232	Dry season	IP10D	6	N49	10.9	-1.233181548	31.19925211	High altitude	3
233	Rain season	6P1D	3	N49	6.3	-1.190480208	31.23488794	High altitude	0
233	Dry season	6P1D	6	N49	9.3	-1.190480208	31.23488794	High altitude	2
234	Rain season	6P4B	3	N49	7.5	-1.193137648	31.23586728	High altitude	0
234	Dry season	6P4B	6	N49	10.5	-1.193137648	31.23586728	High altitude	3
235	Rain season	HP5D	2	N47	7	-1.225660714	31.21152433	High altitude	2
235	Dry season	HP5D	6	N47	10	-1.225660714	31.21152433	High altitude	3
236	Rain season	HR5C	2	N47	6.7	-1.228147381	31.21214989	High altitude	1
236	Dry season	HR5C	6	N47	9.7	-1.228147381	31.21214989	High altitude	3
237	Rain season	3P12C	2	N49	7.8	-1.201652431	31.19999711	High altitude	0
237	Dry season	3P12C	6	N49	10.8	-1.201652431	31.19999711	High altitude	1
238	Rain season	2R11B	2	N41	7.1	-1.201448819	31.18001156	High altitude	0
238	Dry season	2R11B	6	N41	10.1	-1.201448819	31.18001156	High altitude	0
239	Rain season	2R4A	2	N41	6.9	-1.223656597	31.15499656	High altitude	0
239	Dry season	2R4A	6	N41	9.9	-1.223656597	31.15499656	High altitude	0
240	Rain season	3P5B	2	N25	5.5	-1.204582986	31.21872767	High altitude	0
240	Dry season	3P5B	6	N25	8.5	-1.204582986	31.21872767	High altitude	0
241	Rain season	FR4B	2	N25	3.9	-1.200250486	31.34821878	High altitude	1
241	Dry season	FR4B	6	N25	6.9	-1.200250486	31.34821878	High altitude	5
242	Rain season	FR4A	2	N41	5.3	-1.225639698	31.3490305	High altitude	0
242	Dry season	FR4A	6	N41	8.3	-1.225639698	31.3490305	High altitude	2
243	Rain season	5P7B	2	N41	7.6	-1.204664097	31.23315183	High altitude	0
243	Dry season	5P7B	6	N41	10.6	-1.204664097	31.23315183	High altitude	3
244	Rain season	5P7D	2	N41	6.1	-1.205957431	31.23075156	High altitude	0
244	Dry season	5P7D	6	N41	6.1	-1.205957431	31.23075156	High altitude	2
245	Rain season	7P3D	2	N47	5.2	-1.276647509	31.25167362	High altitude	0
245	Dry season	7P3D	6	N47	8.3	-1.276647509	31.25167362	High altitude	2
246	Rain season	7P10A	2	N41	6.2	-1.254639453	31.26764307	High altitude	1
246	Dry season	7P10A	6	N41	9.2	-1.254639453	31.26764307	High altitude	3
247	Rain season	AP1C	2	Mixed	6.9	-1.206195486	31.26127406	High altitude	3
247	Dry season	AP1C	6	Mixed	9.9	-1.206195486	31.26127406	High altitude	2

248	Rain season	AP1D	2	N47	7.6	-1.216277431	31.23852822	High altitude	0
248	Dry season	AP1D	6	N47	10.6	-1.216277431	31.23852822	High altitude	0
249	Rain season	BS2C	2	N47	6.7	-1.203856319	31.27092628	High altitude	0
249	Dry season	BS2C	6	N47	9.7	-1.203856319	31.27092628	High altitude	1
250	Rain season	BR2B	2	N47	6.7	-1.204022153	31.268531	High altitude	0
250	Dry season	BR2B	6	N47	9.7	-1.204022153	31.268531	High altitude	0
251	Rain season	BS4B	2	N47	9.1	-1.209395486	31.27812322	High altitude	1
251	Dry season	BS4B	6	N47	12.1	-1.209395486	31.27812322	High altitude	1
252	Rain season	BP4B	2	N25	7.9	-1.207709097	31.27709461	High altitude	0
252	Dry season	BP4B	6	N25	10.9	-1.207709097	31.27709461	High altitude	0
253	Rain season	TP3A	2	R92/6545	8.6	-1.231397708	31.37007517	High altitude	0
253	Dry season	TP3A	6	R92/6545	11.6	-1.231397708	31.37007517	High altitude	2
254	Rain season	KP5A	2	N25	8.3	-1.217714273	31.3679904	High altitude	0
254	Dry season	KP5A	6	N25	11.3	-1.217714273	31.3679904	High altitude	1
255	Rain season	KP4C	2	N25	6.7	-1.226914551	31.36706957	High altitude	0
255	Dry season	KP4C	6	N25	9.7	-1.226914551	31.36706957	High altitude	5
256	Rain season	KP5D	2	N25	8.5	-1.233134375	31.40366556	High altitude	0
256	Dry season	KP5D	6	N25	11.5	-1.244815102	31.40366556	High altitude	0
257	Rain season	KP5B	2	N25	6.3	-1.220343162	31.36717484	High altitude	0
257	Dry season	KP5B	6	N25	9.3	-1.220343162	31.36717484	High altitude	1
258	Rain season	KS4C	2	Mixed	7.9	-1.228502328	31.36777873	High altitude	0
258	Dry season	KS4C	6	Mixed	10.9	-1.228502328	31.36777873	High altitude	1
259	Rain season	KP4A	2	N25	6.2	-1.22401344	31.36499818	High altitude	1
259	Dry season	KP4A	6	N25	9.2	-1.22401344	31.36499818	High altitude	1
260	Rain season	M5D	2	N47	7.2	-1.231959375	31.28089933	High altitude	0
260	Dry season	M5D	6	N47	10.2	-1.231959375	31.28089933	High altitude	0
261	Rain season	M5C	2	N47	5.1	-1.232350208	31.27643239	High altitude	0
261	Dry season	M5C	6	N47	9.1	-1.232350208	31.27643239	High altitude	1
262	Rain season	HR4A	2	N47	4.8	-1.223769325	31.22176294	High altitude	0
262	Dry season	HR4A	6	N47	8.8	-1.223769325	31.22176294	High altitude	1
263	Rain season	HP4A	2	N47	6.6	-1.225052103	31.21638572	High altitude	0
263	Dry season	HP4A	6	N47	6.6	-1.225052103	31.21638572	High altitude	0
264	Rain season	AP1A	2	R579	8.7	-1.218298819	31.23470461	High altitude	0

264	Dry season	AP1A	6	R579	11.7	-1.218298819	31.23470461	High altitude	0
265	Rain season	AP11A	2	N47	9.1	-1.206195486	31.26127406	High altitude	1
265	Dry season	AP11A	6	N47	13.1	-1.206195486	31.26127406	High altitude	1
266	Rain season	3P7AD	2	N49	8.7	-1.208340486	31.20538322	High altitude	0
266	Dry season	3P7AD	6	N49	12.7	-1.208340486	31.20538322	High altitude	1
267	Rain season	3P7BC	2	N49	9.7	-1.207396042	31.20453822	High altitude	0
267	Dry season	3P7BC	6	N49	13.7	-1.207396042	31.20453822	High altitude	1
268	Rain season	6P6B	2	N41	8.5	-1.194146814	31.25106783	High altitude	0
268	Dry season	6P6B	6	N41	12.5	-1.194146814	31.25106783	High altitude	2
269	Rain season	TR8B	2	N47	11	-1.227337986	31.36852628	High altitude	0
269	Dry season	TR8B	6	N47	15	-1.227337986	31.36852628	High altitude	1
270	Rain season	TR5D	2	N49	8.5	-1.221709097	31.34679517	High altitude	0
270	Dry season	TR5D	6	N49	12.5	-1.221709097	31.34679517	High altitude	0
271	Rain season	M11E	2	N49	8.1	-1.223994653	31.32076933	High altitude	0
271	Dry season	M11E	6	N49	12.1	-1.223994653	31.32076933	High altitude	1
272	Rain season	KR4B	2	N25	5.9	-1.227464551	31.36293012	High altitude	0
272	Dry season	KR4B	6	N25	9.9	-1.227464551	31.36293012	High altitude	1
273	Rain season	M6D	2	N47	6.8	-1.231066319	31.29032961	High altitude	0
273	Dry season	M6D	6	N47	10.8	-1.231066319	31.29032961	High altitude	0
274	Rain season	M6C	2	N41	6.5	-1.231900764	31.28597322	High altitude	1
274	Dry season	M6C	6	N41	10.5	-1.231900764	31.28597322	High altitude	0
275	Rain season	IS4B	2	N47	8	-1.232290826	31.16397555	High altitude	0
275	Dry season	IS4B	6	N47	12	-1.232290826	31.16397555	High altitude	1
276	Rain season	IP4B	2	N47	8	-1.222290826	31.16397555	High altitude	0
276	Dry season	IP4B	6	N47	12	-1.222290826	31.16397555	High altitude	0
277	Rain season	IP7C	2	N47	9.1	-1.273181548	31.11992521	High altitude	0
277	Dry season	IP7C	6	N47	13.1	-1.273181548	31.11992521	High altitude	1
278	Rain season	IP10C	2	N47	11.3	-1.233181548	31.19925211	High altitude	0
278	Dry season	IP10C	6	N47	15.3	-1.233181548	31.19925211	High altitude	0
279	Rain season	IP7B	2	N47	11.1	-1.238767602	31.29309129	High altitude	0
279	Dry season	IP7B	6	N47	15.1	-1.238767602	31.29309129	High altitude	0
280	Rain season	IP10C	2	N47	11.3	-1.227142707	31.13867444	High altitude	0
280	Dry season	IP10C	6	N47	15.3	-1.227142707	31.13867444	High altitude	0

281	Rain season	IS10C	2	N47	11.3	-1.226264009	31.14135222	High altitude	0
281	Dry season	IS10C	6	N47	15.3	-1.226264009	31.14135222	High altitude	0
282	Rain season	IS10A	2	N47	10.5	-1.237262707	31.14446389	High altitude	0
282	Dry season	IS10A	6	N47	14.5	-1.237262707	31.14446389	High altitude	1
283	Rain season	IP10A	2	N47	10.6	-1.226570987	31.13663111	High altitude	0
283	Dry season	IP10A	6	N47	14.6	-1.226570987	31.13663111	High altitude	1
284	Rain season	IS1C	2	N47	10.4	-1.226053492	31.18734822	High altitude	0
284	Dry season	IS1C	6	N47	14.4	-1.226053492	31.18734822	High altitude	1
285	Rain season	IP3B	2	N47	10	-1.226102429	31.14016444	High altitude	0
285	Dry season	IP3B	6	N47	14	-1.226102429	31.14016444	High altitude	0
286	Rain season	IS4F	2	N47	11.4	-1.215571104	31.16590471	High altitude	0
286	Dry season	IS4F	6	N47	15.4	-1.215571104	31.16590471	High altitude	0
287	Rain season	IP2A	2	N47	9.3	-1.243191597	31.15952239	High altitude	0
287	Dry season	IP2A	6	N47	13.3	-1.243191597	31.15952239	High altitude	1
288	Rain season	IS3B	2	N47	10	-1.258036595	31.2705624	High altitude	0
288	Dry season	IS3B	6	N47	14	-1.258036595	31.2705624	High altitude	1
289	Rain season	IP5D	2	N47	9.5	-1.219740271	31.15827055	High altitude	0
289	Dry season	IP5D	6	N47	13.5	-1.219740271	31.15827055	High altitude	1
290	Rain season	HR5C	2	N47	8.5	-1.229636548	31.21445156	High altitude	0
290	Dry season	HR5C	6	N47	12.5	-1.229636548	31.21445156	High altitude	1
291	Rain season	HP5C	2	N47	8.5	-1.228147381	31.21214989	High altitude	0
291	Dry season	HP5C	6	N47	12.5	-1.228147381	31.21214989	High altitude	0
292	Rain season	HR3A	2	N47	7	-1.241128264	31.17846322	High altitude	0
292	Dry season	HR3A	6	N47	11	-1.241128264	31.17846322	High altitude	0
293	Rain season	HS3B	2	N47	6.8	-1.225880714	31.19111628	High altitude	0
293	Dry season	HS3B	6	N47	10.8	-1.225880714	31.19111628	High altitude	1
294	Rain season	IS1F	2	N47	10.8	-1.239221042	31.17536267	High altitude	0
294	Dry season	IS1F	6	N47	14.8	-1.239221042	31.17536267	High altitude	2
295	Rain season	No name	2	N47	7.9	-1.233134375	31.21781461	High altitude	0
295	Dry season	GS4A	6	N47	11.9	-1.233134375	31.21781461	High altitude	1
296	Rain season	OP3A	2	N49	4.2	-1.243134375	31.22781461	High altitude	0
296	Dry season	OP3A	6	N49	12.2	-1.243134375	31.22781461	High altitude	0
297	Rain season	OP3C	2	N47	7.7	-1.259221042	31.18536267	High altitude	0

297	Dry season	OP3C	6	N47	11.7	-1.259221042	31.18536267	High altitude	0
298	Rain season	OP3D	2	N47	7.7	-1.219636548	31.23445156	High altitude	0
298	Dry season	OP3D	6	N47	11.7	-1.219636548	31.23445156	High altitude	0
299	Rain season	OP3B	2	N47	7.6	-1.244815102	31.30212435	High altitude	0
299	Dry season	OP3B	6	N47	11.6	-1.244815102	31.30212435	High altitude	0
300	Rain season	GS4A	2	N47	7.2	-1.233134375	31.21781461	High altitude	0
300	Dry season	GS4A	6	N47	11.2	-1.233134375	31.21781461	High altitude	0
301	Rain season	HP4A	2	N47	8.4	-1.223769325	31.22176294	High altitude	0
301	Dry season	HP4A	6	N47	12.4	-1.223769325	31.22176294	High altitude	1
302	Rain season	GP5D	2	N47	7.9	-1.239112431	31.23383294	High altitude	0
302	Dry season	GP5D	6	N47	11.9	-1.239112431	31.23383294	High altitude	1
303	Rain season	GS2B	2	N47	7	-1.231002937	31.23507017	High altitude	0
303	Dry season	GS2B	6	N47	11	-1.231002937	31.23507017	High altitude	0
304	Rain season	BP7D	2	N47	5.7	-1.190209931	31.28632628	High altitude	0
304	Dry season	BP7D	6	N47	9.7	-1.190209931	31.28632628	High altitude	1
305	Rain season	BS7A	2	N47	4.7	-1.192193264	31.27924878	High altitude	0
305	Dry season	BS7A	6	N47	8.7	-1.192193264	31.27924878	High altitude	0
306	Rain season	BP7C	2	N47	7.1	-1.193766597	31.28763517	High altitude	0
306	Dry season	BP7C	6	N47	11.1	-1.193766597	31.28763517	High altitude	1
307	Rain season	GPSC	2	N47	7.9	-1.241812431	31.2341085	High altitude	0
307	Dry season	GP5C	6	N47	11.9	-1.241812431	31.2341085	High altitude	1
308	Rain season	AP8B	2	N49	7.6	-1.210469931	31.25245683	High altitude	1
308	Dry season	AP8B	6	N49	11.6	-1.210469931	31.25245683	High altitude	0
309	Rain season	AS13D	2	CO617	7.1	-1.221625208	31.26765378	High altitude	0
309	Dry season	AS13D	6	CO617	11.1	-1.221625208	31.26765378	High altitude	0
310	Rain season	JP5D	2	N47	7.3	-1.201625208	31.20765378	High altitude	0
310	Dry season	JP5D	6	N47	11.3	-1.201625208	31.20765378	High altitude	0
311	Rain season	JP1D	2	N47	7.1	-1.251978212	31.24808824	High altitude	1
311	Dry season	JP1D	6	N47	11.1	-1.251978212	31.24808824	High altitude	2
312	Rain season	JP4D	2	N47	7.9	-1.271978212	31.26808824	High altitude	0
312	Dry season	JP4D	6	N47	11.9	-1.271978212	31.26808824	High altitude	0
313	Rain season	JP8C	2	N25	7.5	-1.279389929	31.25575407	High altitude	0
313	Dry season	JP8C	6	N25	11.5	-1.279389929	31.25575407	High altitude	0

314	Rain season	LP2A	2	R579	7.8	-1.259389929	31.25575407	High altitude	0
314	Dry season	LP2A	6	R579	11.8	-1.259389929	31.25575407	High altitude	0
315	Rain season	LS2A	2	N25	7.8	-1.269389929	31.24575407	High altitude	0
315	Dry season	LS2A	6	N25	11.8	-1.269389929	31.24575407	High altitude	2
316	Rain season	LP1C	2	N41	7.7	-1.28465854	31.25142796	High altitude	0
316	Dry season	LP1C	6	N41	11.7	-1.28465854	31.25142796	High altitude	0
317	Rain season	LS1C	2	N41	7.9	-1.26465854	31.23142796	High altitude	1
317	Dry season	LS1C	6	N41	11.9	-1.26465854	31.23142796	High altitude	3
318	Rain season	JP2C	2	N41	9.2	-1.27465854	31.24142796	High altitude	0
318	Dry season	JP2C	6	N41	13.2	-1.27465854	31.24142796	High altitude	0
319	Rain season	JP8B	2	N25	7.5	-1.264342984	31.25672768	High altitude	0
319	Dry season	JP8B	6	N25	11.5	-1.264342984	31.25672768	High altitude	1
320	Rain season	IP1D	2	N41	8.3	-1.242092431	31.17217017	High altitude	0
320	Dry season	IP1D	6	N41	12.3	-1.242092431	31.17217017	High altitude	1
321	Rain season	JS4B	2	N41	7.6	-1.259602431	31.19417156	High altitude	0
321	Dry season	JS4B	6	N41	11.7	-1.259602431	31.19417156	High altitude	0
322	Rain season	IS1D	2	N41	8.3	-1.239602431	31.17417156	High altitude	0
322	Dry season	IS1D	6	N41	12.4	-1.239602431	31.17417156	High altitude	0
323	Rain season	JP4B	2	N25	7.6	-1.274306317	31.25811074	High altitude	0
323	Dry season	JP4B	6	N25	11.7	-1.274306317	31.25811074	High altitude	0
324	Rain season	IP2D	2	N47	8.3	-1.224528492	31.17405211	High altitude	0
324	Dry season	IP2D	6	N47	12.4	-1.224528492	31.17405211	High altitude	0
325	Rain season	JP6B	2	N25	7.7	-1.266735484	31.23890907	High altitude	0
325	Dry season	JP6B	6	N25	11.8	-1.266735484	31.23890907	High altitude	0
1	Rain season	D5	3	R575	8.3	-3.534442207	37.3389079	Medium altitude	1
1	Dry season	D5	7	R575	13.1	-3.534442207	37.3389079	Medium altitude	5
2	Rain season	D3	3	Mixed	4.6	-3.534916318	37.338848	Medium altitude	2
2	Dry season	D3	7	Mixed	9.4	-3.534916318	37.338848	Medium altitude	3
3	Rain season	D2	3	N30	4.8	-3.537965707	37.33825458	Medium altitude	4
3	Dry season	D2	7	N30	9.6	-3.537965707	37.33825458	Medium altitude	4
4	Rain season	D6	3	Mixed	7.8	-3.534441318	37.33863289	Medium altitude	0
4	Dry season	D6	7	Mixed	12.6	-3.534441318	37.33863289	Medium altitude	1
5	Rain season	C3	3	R580	5.5	-3.537267707	37.33810267	Medium altitude	0

5	Dry season	C3	7	R580	10.3	-3.537267707	37.33810267	Medium altitude	2
6	Rain season	C5	3	N30	6.6	-3.535921207	37.3340647	Medium altitude	1
6	Dry season	C5	7	N30	10	-3.535921207	37.3340647	Medium altitude	1
7	Rain season	D9	3	R575	7.7	-3.527982707	37.339803	Medium altitude	1
7	Dry season	D9	7	R575	12.5	-3.527982707	37.339803	Medium altitude	1
8	Rain season	E6	3	R575	7.8	-3.527142707	37.3487613	Medium altitude	0
8	Dry season	E6	7	R575	12.6	-3.527142707	37.3487613	Medium altitude	0
9	Rain season	E3	3	N25	4.9	-3.526264009	37.347792	Medium altitude	0
9	Dry season	E3	7	N25	9.7	-3.526264009	37.347792	Medium altitude	0
10	Rain season	E4	3	N30	4.1	-3.546570987	37.3677353	Medium altitude	0
10	Dry season	E4	7	N30	8.9	-3.546570987	37.3677353	Medium altitude	0
11	Rain season	E1	3	N25	8	-3.526570987	37.3477353	Medium altitude	0
11	Dry season	E1	7	N25	12.8	-3.526570987	37.3477353	Medium altitude	0
12	Rain season	E2	3	N25	4	-3.531720709	37.3431153	Medium altitude	0
12	Dry season	E2	7	N25	8.8	-3.531720709	37.3431153	Medium altitude	1
13	Rain season	E5	3	N25	7.9	-3.526102429	37.3532313	Medium altitude	1
13	Dry season	E5	7	N25	12.7	-3.526102429	37.3532313	Medium altitude	1
14	Rain season	D1	3	M2256/88	3.8	-3.530447409	37.3426687	Medium altitude	0
14	Dry season	D1	7	M2256/88	8.6	-3.530447409	37.3426687	Medium altitude	1
15	Rain season	E7	3	M2256/88	4.9	-3.525919407	37.3532163	Medium altitude	0
15	Dry season	E7	7	M2256/88	9.8	-3.525919407	37.3532163	Medium altitude	0
16	Rain season	C9	3	N25	8.4	-3.514342707	37.3340647	Medium altitude	2
16	Dry season	C9	7	N25	13.2	-3.514342707	37.3340647	Medium altitude	0
17	Rain season	C10	3	N25	8.4	-3.515119207	37.3381163	Medium altitude	0
17	Dry season	C10	7	N25	13.2	-3.515119207	37.3381163	Medium altitude	1
18	Rain season	D15	3	KQ228	6.6	-3.519957707	37.3412797	Medium altitude	0
18	Dry season	D15	7	KQ228	11.4	-3.519957707	37.3412797	Medium altitude	0
19	Rain season	D14	3	Mixed	6.1	-3.503376907	37.3446535	Medium altitude	0
19	Dry season	D14	7	Mixed	10.9	-3.503376907	37.3446535	Medium altitude	1
20	Rain season	D17	3	R580	6.1	-3.520454207	37.3454547	Medium altitude	0
20	Dry season	D17	7	R580	10.5	-3.520454207	37.3454547	Medium altitude	1
21	Rain season	D18	3	M381/95	6	-3.516432707	37.346468	Medium altitude	0
21	Dry season	D18	7	M381/95	10.8	-3.516432707	37.346468	Medium altitude	1

22	Rain season	C12	3	KQ228	4.8	-3.511286107	37.338818	Medium altitude	0
22	Dry season	C12	7	KQ228	9.6	-3.511286107	37.338818	Medium altitude	1
23	Rain season	D31	3	M225/88	3.7	-3.514899002	37.3487683	Medium altitude	0
23	Dry season	D31	7	M225/88	8.5	-3.514899002	37.3487683	Medium altitude	0
24	Rain season	D27	3	N25	6.5	-3.519142091	37.3482583	Medium altitude	0
24	Dry season	D27	7	N25	11.3	-3.519142091	37.3482583	Medium altitude	1
25	Rain season	D30	3	N25	6.2	-3.515009002	37.3490317	Medium altitude	0
25	Dry season	D30	7	N25	11	-3.515009002	37.3490317	Medium altitude	1
26	Rain season	E14	3	N25	6.2	-3.509162207	37.3521413	Medium altitude	0
26	Dry season	E14	7	N25	11	-3.509162207	37.3521413	Medium altitude	1
27	Rain season	D28	3	R580	6	-3.518923702	37.3481083	Medium altitude	0
27	Dry season	D28	7	R580	10.8	-3.518923702	37.3481083	Medium altitude	0
28	Rain season	D20	3	N30	4.1	-3.503390518	37.347233	Medium altitude	0
28	Dry season	D20	7	N30	8.9	-3.503390518	37.347233	Medium altitude	0
29	Rain season	D25	3	M2256/88	6	-3.504659929	37.3523113	Medium altitude	0
29	Dry season	D25	7	M2256/88	10.8	-3.504659929	37.3523113	Medium altitude	0
30	Rain season	D24	3	R585	6.3	-3.505028507	37.3527163	Medium altitude	0
30	Dry season	D24	7	R585	11.1	-3.505028507	37.3527163	Medium altitude	1
31	Rain season	D22	3	R585	3.8	-3.508861007	37.3517763	Medium altitude	0
31	Dry season	D22	7	R585	8.6	-3.508861007	37.3517763	Medium altitude	1
32	Rain season	BO1	3	R579	5.1	-3.510589407	37.3375363	Medium altitude	0
32	Dry season	BO1	7	R579	9.9	-3.510589407	37.3375363	Medium altitude	0
33	Rain season	B2	3	R579	4.2	-3.510552707	37.3374963	Medium altitude	0
33	Dry season	B2	7	R579	9	-3.510552707	37.3374963	Medium altitude	0
34	Rain season	BO5	3	N25	5.6	-3.512716224	37.336425	Medium altitude	0
34	Dry season	BO5	7	N25	10.4	-3.512716224	37.336425	Medium altitude	0
35	Rain season	E18	3	N25	5.1	-3.511519002	37.35413448	Medium altitude	0
35	Dry season	E18	7	N25	9.9	-3.511519002	37.35413448	Medium altitude	0
36	Rain season	E16	3	N25	6	-3.508699591	37.3528233	Medium altitude	0
36	Dry season	E16	7	N25	10.8	-3.508699591	37.3528233	Medium altitude	2
37	Rain season	E15	3	M2256/88	8.8	-3.505794407	37.3570047	Medium altitude	0
37	Dry season	E15	7	M2256/88	13.4	-3.505794407	37.3570047	Medium altitude	1
38	Rain season	E9	3	R94/6113	4.8	-3.521721007	37.3541463	Medium altitude	0

38	Dry season	E9	7	R94/6113	9.6	-3.521721007	37.3541463	Medium altitude	1
39	Rain season	BO3	3	R579	10.6	-3.515030702	37.33483	Medium altitude	0
39	Dry season	BO3	7	R579	4.6	-3.515030702	37.33483	Medium altitude	0
40	Rain season	E10	3	N25	7.6	-3.521736007	37.3537247	Medium altitude	0
40	Dry season	E10	7	N25	12.4	-3.521736007	37.3537247	Medium altitude	0
41	Rain season	KH34	3	M381/95	5.5	-3.498669006	37.41755801	Medium altitude	0
41	Dry season	KH34	7	M381/95	10.3	-3.498669006	37.41755801	Medium altitude	1
42	Rain season	KH39	3	M381/95	5.6	-3.497720706	37.41788131	Medium altitude	0
42	Dry season	KH39	7	M381/95	10.4	-3.497720706	37.41788131	Medium altitude	0
43	Rain season	KH10	3	R579	6.9	-3.508632254	37.42313375	Medium altitude	0
43	Dry season	KH10	7	R579	11.7	-3.508632254	37.42313375	Medium altitude	0
44	Rain season	KH8	3	R580	7.2	-3.504667254	37.42332375	Medium altitude	0
44	Dry season	KH8	7	R580	12	-3.504667254	37.42332375	Medium altitude	1
45	Rain season	KH2	3	KQ228	4.5	-3.511887406	37.41011471	Medium altitude	0
45	Dry season	KH2	7	KQ228	9.3	-3.511887406	37.41011471	Medium altitude	0
46	Rain season	KH31	3	N25	5.6	-3.501079006	37.41667631	Medium altitude	0
46	Dry season	KH31	7	N25	9.4	-3.501079006	37.41667631	Medium altitude	1
47	Rain season	KH1	3	N30	4.5	-3.506432406	37.41409631	Medium altitude	0
47	Dry season	KH1	7	N30	9.3	-3.506432406	37.41409631	Medium altitude	2
48	Rain season	KH3	3	KQ228	4.5	-3.511977406	37.41016131	Medium altitude	0
48	Dry season	KH3	7	KQ228	9.3	-3.511977406	37.41016131	Medium altitude	1
49	Rain season	KH18	3	N25	5.1	-3.503960702	37.413814	Medium altitude	0
49	Dry season	KH18	7	N25	9.9	-3.503960702	37.413814	Medium altitude	1
50	Rain season	KH19	3	R94/6113	5.1	-3.503844002	37.4136357	Medium altitude	0
50	Dry season	KH19	7	R94/6113	9.9	-3.503844002	37.4136357	Medium altitude	1
51	Rain season	KH40	3	M381/95	5.1	-3.500437402	37.413939	Medium altitude	1
51	Dry season	KH40	7	M381/95	9.9	-3.500437402	37.413939	Medium altitude	0
52	Rain season	KH41	3	R94/6113	6.3	-3.498429002	37.4090057	Medium altitude	0
52	Dry season	KH41	7	R94/6113	11.1	-3.498429002	37.4090057	Medium altitude	0
53	Rain season	KH17	3	N25	5.1	-3.507500702	37.413679	Medium altitude	0
53	Dry season	KH17	7	N25	9.9	-3.507500702	37.413679	Medium altitude	2
54	Rain season	KH20	3	R94/6113	3.1	-3.507335702	37.4133957	Medium altitude	0
54	Dry season	KH20	7	R94/6113	7.9	-3.507335702	37.4133957	Medium altitude	0

55	Rain season	KH16	3	N25	5.6	-3.510974002	37.413489	Medium altitude	0
55	Dry season	KH16	7	N25	10.4	-3.510974002	37.413489	Medium altitude	0
56	Rain season	KH21	3	N25	3.6	-3.510820702	37.4131323	Medium altitude	0
56	Dry season	KH21	7	N25	8.4	-3.510820702	37.4131323	Medium altitude	0
57	Rain season	KH44	3	N25	4.2	-3.498379002	37.4088273	Medium altitude	0
57	Dry season	KH44	7	N25	9	-3.498379002	37.4088273	Medium altitude	1
58	Rain season	KH45	3	N25	8.8	-3.499009002	37.403509	Medium altitude	1
58	Dry season	KH45	7	N25	13.6	-3.499009002	37.403509	Medium altitude	2
59	Rain season	KH25	3	R579	8.5	-3.518475329	37.3862627	Medium altitude	0
59	Dry season	KH25	7	R579	13.3	-3.518475329	37.3862627	Medium altitude	0
60	Rain season	KH24	3	R575	5.2	-3.519080702	37.3864717	Medium altitude	0
60	Dry season	KH24	7	R575	10	-3.519080702	37.3864717	Medium altitude	0
61	Rain season	KH13	3	N30	5	-3.503214407	37.402403	Medium altitude	0
61	Dry season	KH13	7	N30	9.9	-3.503214407	37.402403	Medium altitude	0
62	Rain season	KH14	3	N25	6.5	-3.503139818	37.40239303	Medium altitude	0
62	Dry season	KH14	7	N25	11.3	-3.503139818	37.40239303	Medium altitude	0
63	Rain season	11F	3	N25	8.6	-3.506497402	37.34815	Medium altitude	0
63	Dry season	11F	7	N25	13.4	-3.506497402	37.34815	Medium altitude	0
64	Rain season	11F5	3	N25	3.1	-3.502727402	37.3481517	Medium altitude	0
64	Dry season	11F5	7	N25	7.9	-3.502727402	37.3481517	Medium altitude	0
65	Rain season	KH30	3	N25	7.8	-3.5028124	37.4030607	Medium altitude	0
65	Dry season	KH30	7	N25	12.6	-3.5028124	37.4030607	Medium altitude	0
66	Rain season	KH29	3	R579	8.1	-3.506320702	37.4027607	Medium altitude	0
66	Dry season	KH29	7	R579	12.9	-3.506320702	37.4027607	Medium altitude	0
67	Rain season	KH22	3	N25	3.9	-3.499332707	37.402383	Medium altitude	1
67	Dry season	KH22	7	N25	8.7	-3.499332707	37.402383	Medium altitude	3
68	Rain season	KH15	3	N25	6.6	-3.499602707	37.402603	Medium altitude	0
68	Dry season	KH15	7	N25	11.4	-3.499602707	37.402603	Medium altitude	0
69	Rain season	11C2	3	N25	8.6	-3.491587402	37.351545	Medium altitude	0
69	Dry season	11C2	7	N25	13.4	-3.491587402	37.351545	Medium altitude	0
70	Rain season	11C1	3	N25	8.5	-3.500464152	37.3719292	Medium altitude	0
70	Dry season	11C1	7	N25	13.3	-3.500464152	37.3719292	Medium altitude	0
71	Rain season	11B1	3	N25	8.7	-3.490464152	37.3519292	Medium altitude	0

71	Dry season	11B1	7	N25	13.5	-3.490464152	37.3519292	Medium altitude	0
72	Rain season	11A	3	N25	8.7	-3.496710702	37.3526033	Medium altitude	0
72	Dry season	11A	7	N25	13.5	-3.496710702	37.3526033	Medium altitude	0
73	Rain season	11B	3	N25	8.7	-3.494970702	37.35378	Medium altitude	0
73	Dry season	11B	7	N25	13.5	-3.494970702	37.35378	Medium altitude	0
74	Rain season	11	3	R579	8.7	-3.497244002	37.3519383	Medium altitude	0
74	Dry season	11	7	R579	13.5	-3.497244002	37.3519383	Medium altitude	0
75	Rain season	10L	3	N25	7.8	-3.488350702	37.34317	Medium altitude	0
75	Dry season	10L	7	N25	12.6	-3.488350702	37.34317	Medium altitude	0
76	Rain season	10K	3	R579	5.2	-3.523862707	37.3259813	Medium altitude	0
76	Dry season	10K	7	R579	10	-3.523862707	37.3259813	Medium altitude	0
77	Rain season	A1	3	KQ228	3.9	-3.521182707	37.3270913	Medium altitude	0
77	Dry season	A1	7	KQ228	7.7	-3.521182707	37.3270913	Medium altitude	0
78	Rain season	B4	3	N25	6.9	-3.511326007	37.328393	Medium altitude	0
78	Dry season	B4	7	N25	11.7	-3.511326007	37.328393	Medium altitude	0
79	Rain season	A4	3	R575	4.6	-3.511326007	37.328393	Medium altitude	0
79	Dry season	A4	7	R575	9.4	-3.511326007	37.328393	Medium altitude	1
80	Rain season	A5	3	N25	4.6	-3.508327707	37.3289397	Medium altitude	0
80	Dry season	A5	7	N25	9.4	-3.508327707	37.3289397	Medium altitude	0
81	Rain season	A3	3	R579	4.9	-3.513814407	37.327833	Medium altitude	0
81	Dry season	A3	8	R579	9.6	-3.513814407	37.327833	Medium altitude	0
82	Rain season	B5	3	N25	8.6	-3.515531007	37.3281063	Medium altitude	0
82	Dry season	B5	8	N25	11.6	-3.515531007	37.3281063	Medium altitude	1
83	Rain season	A6	3	N25	4.6	-3.520882402	37.324855	Medium altitude	0
83	Dry season	A6	8	N25	9.5	-3.520882402	37.324855	Medium altitude	1
84	Rain season	B10	3	M700/86	4	-3.518089002	37.328805	Medium altitude	0
84	Dry season	B10	8	M700/86	8.6	-3.518089002	37.328805	Medium altitude	2
85	Rain season	O2	3	R579	6.2	-3.513809002	37.3288117	Medium altitude	0
85	Dry season	O2	8	R579	11.1	-3.513809002	37.3288117	Medium altitude	2
86	Rain season	B6	3	N25	8.2	-3.515116007	37.328033	Medium altitude	0
86	Dry season	B6	8	N25	12.4	-3.515116007	37.328033	Medium altitude	1
87	Rain season	O4	3	R579	4.8	-3.505925702	37.339995	Medium altitude	0
87	Dry season	O4	8	R579	9.6	-3.505925702	37.339995	Medium altitude	2

88	Rain season	G2	3	R579	5.1	-3.473332402	37.3345883	Medium altitude	0
88	Dry season	G2	8	R579	9.9	-3.473332402	37.3345883	Medium altitude	1
89	Rain season	G5	3	R579	8.2	-3.468965702	37.33397	Medium altitude	0
89	Dry season	G5	8	R579	11.3	-3.468965702	37.33397	Medium altitude	2
90	Rain season	G6	3	R579	5.6	-3.469004002	37.3337433	Medium altitude	0
90	Dry season	G6	8	R579	11	-3.469004002	37.3337433	Medium altitude	1
91	Rain season	17A	3	M2256/88	4.1	-3.457857402	37.3247583	Medium altitude	0
91	Dry season	17A	8	M2256/88	9.5	-3.457857402	37.3247583	Medium altitude	1
92	Rain season	G1	3	N25	5.3	-3.477507402	37.3354817	Medium altitude	0
92	Dry season	G1	8	N25	11.5	-3.477507402	37.3354817	Medium altitude	1
93	Rain season	G3	3	N25	7.2	-3.477522402	37.335915	Medium altitude	0
93	Dry season	G3	8	N25	11.9	-3.477522402	37.335915	Medium altitude	2
94	Rain season	14	3	N25	8.1	-3.427929407	37.3204747	Medium altitude	0
94	Dry season	14	8	N25	12.1	-3.427929407	37.3204747	Medium altitude	1
95	Rain season	12A	3	N25	6.4	-3.457802402	37.31259	Medium altitude	0
95	Dry season	12A	8	N25	9.8	-3.457802402	37.31259	Medium altitude	1
96	Rain season	12B	3	N25	8.6	-3.457855702	37.3131433	Medium altitude	0
96	Dry season	12B	8	N25	11.9	-3.457855702	37.3131433	Medium altitude	0
97	Rain season	12C	3	M381/95	5.4	-3.450927402	37.3145367	Medium altitude	0
97	Dry season	12C	8	M381/95	11.6	-3.450927402	37.3145367	Medium altitude	0
98	Rain season	12	3	M381/95	5.1	-3.458105702	37.3126233	Medium altitude	0
98	Dry season	12	8	M381/95	10.5	-3.458105702	37.3126233	Medium altitude	1
99	Rain season	2C	3	M381/95	7.3	-3.494575702	37.31232	Medium altitude	0
99	Dry season	2C	8	M381/95	10.8	-3.494575702	37.31232	Medium altitude	1
100	Rain season	73	3	R579	6.7	-3.531816074	37.3148213	Medium altitude	0
100	Dry season	73	8	R579	11	-3.531816074	37.3148213	Medium altitude	0
101	Rain season	4	3	R585	5.7	-3.466129407	37.311668	Medium altitude	0
101	Dry season	4	8	R585	10.6	-3.466129407	37.311668	Medium altitude	0
102	Rain season	3	3	N25	8.7	-3.469961007	37.3106963	Medium altitude	0
102	Dry season	3	8	N25	12.8	-3.469961007	37.3106963	Medium altitude	2
103	Rain season	2	3	R579	7.9	-3.474357707	37.3113013	Medium altitude	0
103	Dry season	2	8	R579	12.7	-3.474357707	37.3113013	Medium altitude	2
104	Rain season	3B	3	R585	7.9	-3.494139002	37.3121417	Medium altitude	0

104	Dry season	3B	8	R585	12.8	-3.494139002	37.3121417	Medium altitude	1
105	Rain season	4D	3	KQ228	7.7	-3.508509002	37.3509367	Medium altitude	0
105	Dry season	4D	8	KQ228	12.6	-3.508509002	37.3509367	Medium altitude	0
106	Rain season	5F	3	R579	5.2	-3.488509002	37.3209367	Medium altitude	0
106	Dry season	5F	8	R579	10.1	-3.488509002	37.3209367	Medium altitude	0
107	Rain season	6C	3	R579	5.6	-3.483460702	37.3182383	Medium altitude	0
107	Dry season	6C	8	R579	11.1	-3.483460702	37.3182383	Medium altitude	0
108	Rain season	2AEYP	3	R579	4.1	-3.477662707	37.310368	Medium altitude	0
108	Dry season	2AEYP	8	R579	9	-3.477662707	37.310368	Medium altitude	0
109	Rain season	N21	3	N25	7.8	-3.550426007	37.3203713	Medium altitude	0
109	Dry season	N21	8	N25	12.7	-3.550426007	37.3203713	Medium altitude	0
110	Rain season	L1N	3	R579	5	-3.549959407	37.319888	Medium altitude	0
110	Dry season	L1N	8	R579	9.9	-3.549959407	37.319888	Medium altitude	2
111	Rain season	L2S	3	R579	6.1	-3.547109407	37.3137647	Medium altitude	0
111	Dry season	L2S	8	R579	10.9	-3.547109407	37.3137647	Medium altitude	0
112	Rain season	L2N	3	R579	4.5	-3.547908009	37.3203057	Medium altitude	0
112	Dry season	L2N	8	R579	9.7	-3.547908009	37.3203057	Medium altitude	0
113	Rain season	K4N	3	Mixed	4.1	-3.541507707	37.318603	Medium altitude	0
113	Dry season	K4N	8	Mixed	10.6	-3.541507707	37.318603	Medium altitude	0
114	Rain season	K3	3	R579	6.7	-3.533511007	37.322643	Medium altitude	0
114	Dry season	K3	8	R579	11.6	-3.533511007	37.322643	Medium altitude	1
115	Rain season	K2	3	R579	5.7	-3.541629407	37.3186497	Medium altitude	0
115	Dry season	K2	8	R579	10.2	-3.541629407	37.3186497	Medium altitude	0
116	Rain season	K6N	3	N25	3.8	-3.528661007	37.3215813	Medium altitude	0
116	Dry season	K6N	8	N25	8.5	-3.528661007	37.3215813	Medium altitude	0
117	Rain season	B2	3	R579	7	-3.527182707	37.3313447	Medium altitude	0
117	Dry season	B2	8	R579	11.9	-3.527182707	37.3313447	Medium altitude	1
118	Rain season	B1	3	N25	3.5	-3.531606007	37.3305697	Medium altitude	0
118	Dry season	B1	8	N25	8.4	-3.531606007	37.3305697	Medium altitude	0
119	Rain season	4F	3	N25	5.1	-3.493475702	37.323775	Medium altitude	0
119	Dry season	4F	8	N25	10	-3.493475702	37.323775	Medium altitude	0
120	Rain season	3F	3	KQ228	8.6	-3.493565702	37.3237483	Medium altitude	0
120	Dry season	3F	8	KQ228	13.3	-3.493565702	37.3237483	Medium altitude	0

121	Rain season	9C	3	R579	8.6	-3.504439002	37.3230017	Medium altitude	0
121	Dry season	9C	8	R579	13.5	-3.504439002	37.3230017	Medium altitude	1
122	Rain season	9B	3	N25	8.6	-3.504154002	37.321065	Medium altitude	0
122	Dry season	9B	8	N25	13.8	-3.504154002	37.321065	Medium altitude	2
123	Rain season	F3	3	N25	4.7	-3.505947402	37.3204267	Medium altitude	0
123	Dry season	F3	8	N25	9.6	-3.505947402	37.3204267	Medium altitude	1
124	Rain season	10D	3	N25	7.8	-3.486089002	37.3398367	Medium altitude	0
124	Dry season	10D	8	N25	12.4	-3.486089002	37.3398367	Medium altitude	1
125	Rain season	9	3	N25	4.8	-3.490762402	37.336965	Medium altitude	0
125	Dry season	9	8	N25	9.7	-3.490762402	37.336965	Medium altitude	1
126	Rain season	10E	3	R579	3.8	-3.490750702	37.3380583	Medium altitude	0
126	Dry season	10E	8	R579	9.1	-3.490750702	37.3380583	Medium altitude	0
127	Rain season	10A	3	N25	4.3	-3.494794002	37.33871	Medium altitude	0
127	Dry season	10A	8	N25	9.2	-3.494794002	37.33871	Medium altitude	0
128	Rain season	10	3	N25	4.3	-3.494915702	37.338705	Medium altitude	0
128	Dry season	10	8	N25	9.2	-3.494915702	37.338705	Medium altitude	2
129	Rain season	Q15	3	M381/95	4.9	-3.538047007	37.3176119	Medium altitude	0
129	Dry season	Q1S	8	M381/95	9.8	-3.538047007	37.3176119	Medium altitude	3
130	Rain season	R6N	3	N25	6.1	-3.503616007	37.3156397	Medium altitude	0
130	Dry season	R6N	8	N25	10.9	-3.503616007	37.3156397	Medium altitude	0
131	Rain season	H4	3	R579	5.3	-3.490152707	37.309898	Medium altitude	0
131	Dry season	H4	8	R579	9.1	-3.490152707	37.309898	Medium altitude	1
132	Rain season	F15	3	N25	3.8	-3.503781007	37.3273347	Medium altitude	0
132	Dry season	F1S	8	N25	9.1	-3.503781007	37.3273347	Medium altitude	1
133	Rain season	Q3S	3	R94/6113	3.6	-3.509434709	37.3125473	Medium altitude	0
133	Dry season	Q3S	8	R94/6113	9	-3.509434709	37.3125473	Medium altitude	0
134	Rain season	H3	3	R579	5.6	-3.490906007	37.314283	Medium altitude	0
134	Dry season	H3	8	R579	10.5	-3.490906007	37.314283	Medium altitude	0
135	Rain season	H1	3	R579	7.4	-3.488484407	37.3128413	Medium altitude	0
135	Dry season	H1	8	R579	12.3	-3.488484407	37.3128413	Medium altitude	0
136	Rain season	R7N	3	N25	7.2	-3.500776409	37.315634	Medium altitude	0
136	Dry season	R7N	8	N25	12.1	-3.500776409	37.315634	Medium altitude	0
137	Rain season	R5N	3	N25	7.4	-3.509211409	37.3122057	Medium altitude	0

137	Dry season	R5N	8	N25	13.3	-3.509211409	37.3122057	Medium altitude	0
138	Rain season	R4N	3	R579	8	-3.508821409	37.3123723	Medium altitude	1
138	Dry season	R4N	8	R579	13.8	-3.508821409	37.3123723	Medium altitude	3
139	Rain season	P3N	3	KQ228	4	-3.527089407	37.3153197	Medium altitude	0
139	Dry season	P3N	8	KQ228	8.9	-3.527089407	37.3153197	Medium altitude	0
140	Rain season	P1S	3	R579	5.6	-3.527199407	37.3153097	Medium altitude	0
140	Dry season	P1S	8	R579	9.5	-3.527199407	37.3153097	Medium altitude	1
141	Rain season	P2S	3	R579	3.8	-3.526524709	37.321114	Medium altitude	0
141	Dry season	P2S	8	R579	8.6	-3.526524709	37.321114	Medium altitude	0
142	Rain season	P2N	3	R579	6	-3.527704709	37.3161073	Medium altitude	0
142	Dry season	P2N	8	R579	11	-3.527704709	37.3161073	Medium altitude	1
143	Rain season	Q3N	3	R579	7.8	-3.515701409	37.318879	Medium altitude	1
143	Dry season	Q3N	8	R579	13.8	-3.515701409	37.318879	Medium altitude	2
144	Rain season	Q2N	3	R575	3.8	-3.515649709	37.319154	Medium altitude	0
144	Dry season	Q2N	8	R575	9.4	-3.515649709	37.319154	Medium altitude	0
145	Rain season	P3S	3	R579	4.6	-3.519104407	37.3173163	Medium altitude	0
145	Dry season	P3S	8	R579	8.6	-3.519104407	37.3173163	Medium altitude	0
146	Rain season	M5	3	KQ228	4.7	-3.517826409	37.3095657	Medium altitude	0
146	Dry season	M5	8	KQ228	9.7	-3.517826409	37.3095657	Medium altitude	0
147	Rain season	M6	3	R579	6.6	-3.517793009	37.3092307	Medium altitude	0
147	Dry season	M6	8	R579	10.1	-3.517793009	37.3092307	Medium altitude	0
148	Rain season	M4	3	R579	5.6	-3.532271409	37.3120173	Medium altitude	0
148	Dry season	M4	8	R579	10.3	-3.532271409	37.3120173	Medium altitude	0
149	Rain season	M3	3	R579	5.6	-3.531258009	37.3165423	Medium altitude	0
149	Dry season	M3	8	R579	11.9	-3.531258009	37.3165423	Medium altitude	0
150	Rain season	Q1N	3	N25	3.8	-3.519002707	37.317348	Medium altitude	0
150	Dry season	Q1N	8	N25	9.5	-3.519002707	37.317348	Medium altitude	0
151	Rain season	N34S	3	R579	4.2	-3.489410729	37.3057303	Medium altitude	1
151	Dry season	N34S	8	R579	8.6	-3.489410729	37.3057303	Medium altitude	1
152	Rain season	N60	3	M2256/88	4.2	-3.511660729	37.314622	Medium altitude	0
152	Dry season	N60	8	M2256/88	9.4	-3.511660729	37.314622	Medium altitude	0
153	Rain season	N74	3	R579	4.5	-3.515987429	37.31756869	Medium altitude	0
153	Dry season	N74	8	R579	9.5	-3.515987429	37.31756869	Medium altitude	0

154	Rain season	N100KIBO	3	R575	4.6	-3.544165729	37.321857	Medium altitude	0
154	Dry season	N100KIBO	8	R575	8.7	-3.544165729	37.321857	Medium altitude	1
155	Rain season	N80	3	N25	7.6	-3.521382429	37.3197787	Medium altitude	0
155	Dry season	N80	8	N25	12.5	-3.521382429	37.3197787	Medium altitude	0
156	Rain season	N81	3	R575	3.8	-3.532684029	37.3255753	Medium altitude	0
156	Dry season	N81	8	R575	7.6	-3.532684029	37.3255753	Medium altitude	2
157	Rain season	N82	3	R575	5.3	-3.533970729	37.3349903	Medium altitude	0
157	Dry season	N82	8	R575	10.7	-3.533970729	37.3349903	Medium altitude	1
158	Rain season	N92	3	R579	6.2	-3.521255729	37.3366837	Medium altitude	0
158	Dry season	N92	8	R579	11.1	-3.521255729	37.3366837	Medium altitude	1
159	Rain season	N91	3	R579	6.4	-3.534377429	37.3391003	Medium altitude	0
159	Dry season	N91	8	R579	11.8	-3.534377429	37.3391003	Medium altitude	0
160	Rain season	N63	3	N30	6.5	-3.510957429	37.3285853	Medium altitude	0
160	Dry season	N63	8	N30	11.5	-3.510957429	37.3285853	Medium altitude	1
161	Rain season	N51	3	R579	4.1	-3.519132023	37.3150223	Medium altitude	0
161	Dry season	N51	8	R579	9.9	-3.519132023	37.3150223	Medium altitude	0
162	Rain season	N42	3	R579	5.1	-3.518747023	37.3155757	Medium altitude	0
162	Dry season	N42	8	R579	11.1	-3.518747023	37.3155757	Medium altitude	0
163	Rain season	N44	3	Mixed	7	-3.510008723	37.3129973	Medium altitude	0
163	Dry season	N44	8	Mixed	11.9	-3.510008723	37.3129973	Medium altitude	0
164	Rain season	N73	3	R579	7.3	-3.521340729	37.330597	Medium altitude	0
164	Dry season	N73	8	R579	12.3	-3.521340729	37.330597	Medium altitude	1
165	Rain season	N41	3	R579	5.3	-3.514130423	37.3140073	Medium altitude	0
165	Dry season	N41	8	R579	9.8	-3.514130423	37.3140073	Medium altitude	0
166	Rain season	N52	3	R585	5.1	-3.519035423	37.3153657	Medium altitude	0
166	Dry season	N52	8	R585	10.3	-3.519035423	37.3153657	Medium altitude	1
167	Rain season	N65	3	R579	6.5	-3.506485729	37.321797	Medium altitude	0
167	Dry season	N65	8	R579	11.4	-3.506485729	37.321797	Medium altitude	0
168	Rain season	N72	3	R579	7.3	-3.521314029	37.324717	Medium altitude	0
168	Dry season	N72	8	R579	12.5	-3.521314029	37.324717	Medium altitude	0
169	Rain season	N100MN	3	KQ228	4.8	-3.510214618	37.3343953	Medium altitude	0
169	Dry season	N100MN	8	KQ228	9.7	-3.510214618	37.3343953	Medium altitude	0
170	Rain season	N50	3	R579	7.2	-3.502415729	37.3097403	Medium altitude	0

170	Dry season	N50	8	R579	12.1	-3.502415729	37.3097403	Medium altitude	1
171	Rain season	B02	3	R579	5.3	-3.547423707	37.334347	Medium altitude	1
171	Dry season	B02	8	R579	10.1	-3.547423707	37.334347	Medium altitude	1
172	Rain season	D19	3	M381/95	4.1	-3.524788907	37.3373539	Medium altitude	0
172	Dry season	D19	8	M381/95	8.5	-3.524788907	37.3373539	Medium altitude	0
173	Rain season	C4	3	N30	4	-3.483097502	37.32686	Medium altitude	0
173	Dry season	C4	8	N30	8.8	-3.483097502	37.32686	Medium altitude	1
174	Rain season	N84	3	N25	4.9	-3.483097502	37.32686	Medium altitude	0
174	Dry season	N84	8	N25	9.7	-3.483097502	37.32686	Medium altitude	0
175	Rain season	11Fs	3	R579	4	-3.503097502	37.34686	Medium altitude	1
175	Dry season	11Fs	8	R579	8.8	-3.503097502	37.34686	Medium altitude	1
1	Rain season	339	3	R579	6.6	-7.658385597	36.9866648	Low altitude	0
1	Dry season	339	9	R579	11.8	-7.658385597	36.9866648	Low altitude	0
2	Rain season	338	3	MN1	6.4	-7.587094095	37.0070119	Low altitude	1
2	Dry season	338	9	MN1	11.6	-7.587094095	37.0070119	Low altitude	1
3	Rain season	356	3	N36	9.1	-7.647722064	36.9928406	Low altitude	1
3	Dry season	356	9	N36	14.3	-7.647722064	36.9928406	Low altitude	1
4	Rain season	803	3	R570	4.8	-7.614185089	37.00004608	Low altitude	0
4	Dry season	803	9	R570	10	-7.614185089	37.00004608	Low altitude	0
5	Rain season	801	3	R570	5.3	-7.617732539	37.00004608	Low altitude	0
5	Dry season	801	9	R570	10.5	-7.617732539	37.00004608	Low altitude	0
6	Rain season	649	3	R570	5.1	-7.600778382	37.00775022	Low altitude	0
6	Dry season	649	9	R570	10.3	-7.600778382	37.00775022	Low altitude	0
7	Rain season	664	3	R570	4.8	-7.598244054	37.016695	Low altitude	0
7	Dry season	664	9	R570	10	-7.598244054	37.016695	Low altitude	0
8	Rain season	667	3	R570	5.7	-7.585262835	37.00778221	Low altitude	0
8	Dry season	667	9	R570	10.9	-7.585262835	37.00778221	Low altitude	0
9	Rain season	670	3	N41	5.7	-7.574398697	37.0122497	Low altitude	0
9	Dry season	670	9	N41	10.9	-7.574398697	37.0122497	Low altitude	0
10	Rain season	277	3	R570	4.6	-7.586519693	37.00765869	Low altitude	1
10	Dry season	277	9	R570	9.8	-7.586519693	37.00765869	Low altitude	1
11	Rain season	680	3	R570	5.9	-7.575628097	37.0115748	Low altitude	0
11	Dry season	680	9	R570	11.1	-7.575628097	37.0115748	Low altitude	0

12	Rain season	679	3	R570	6	-7.575705697	37.0115962	Low altitude	0
12	Dry season	679	9	R570	11.2	-7.575705697	37.0115962	Low altitude	0
13	Rain season	674	3	R570	6.3	-7.639992797	36.9903728	Low altitude	0
13	Dry season	674	9	R570	11.5	-7.639992797	36.9903728	Low altitude	0
14	Rain season	687	3	N19	5.9	-7.584480096	37.0115864	Low altitude	0
14	Dry season	687	9	N19	11.1	-7.584480096	37.0115864	Low altitude	0
15	Rain season	408	3	R579	6.1	-7.586519693	37.00765869	Low altitude	0
15	Dry season	408	9	R579	11.3	-7.586519693	37.00765869	Low altitude	0
16	Rain season	407	3	R579	8.5	-7.575628097	37.0115748	Low altitude	0
16	Dry season	407	9	R579	13.7	-7.575628097	37.0115748	Low altitude	1
17	Rain season	411	3	R579	9	-7.577012696	37.0135567	Low altitude	1
17	Dry season	411	9	R579	14.3	-7.577012696	37.0135567	Low altitude	3
18	Rain season	406	3	N25	4.1	-7.639995097	36.9903491	Low altitude	1
18	Dry season	406	9	N25	9.3	-7.639995097	36.9903491	Low altitude	3
19	Rain season	403	3	R579	6.1	-7.594941393	37.01082069	Low altitude	0
19	Dry season	403	9	R579	11.3	-7.594941393	37.01082069	Low altitude	1
20	Rain season	432	3	R579	8.9	-7.586519693	37.00765869	Low altitude	0
20	Dry season	432	9	R579	14.1	-7.586519693	37.00765869	Low altitude	0
21	Rain season	433	3	R570	8.3	-7.594941393	37.01082069	Low altitude	0
21	Dry season	433	9	R570	13.5	-7.594941393	37.01082069	Low altitude	0
22	Rain season	527	3	R579	7.6	-7.669795882	37.03151759	Low altitude	0
22	Dry season	527	9	R579	12.8	-7.669795882	37.03151759	Low altitude	0
23	Rain season	554	3	R579	8.6	-7.678387882	37.03448549	Low altitude	0
23	Dry season	554	9	R579	13.8	-7.678387882	37.03448549	Low altitude	1
24	Rain season	622-B	3	R570	4.9	-7.653037082	37.00844879	Low altitude	0
24	Dry season	622-B	9	R570	10.1	-7.653037082	37.00844879	Low altitude	0
25	Rain season	601	3	R570	8.4	-7.661067582	37.01010279	Low altitude	0
25	Dry season	601	9	R570	13.6	-7.661067582	37.01010279	Low altitude	0
26	Rain season	510	3	R579	9.1	-7.645039397	37.00016069	Low altitude	0
26	Dry season	510	9	R579	3.2	-7.645039397	37.00016069	Low altitude	0
27	Rain season	507	3	R579	7.7	-7.645128997	37.00019429	Low altitude	0
27	Dry season	507	9	R579	12.9	-7.645128997	37.00019429	Low altitude	0
28	Rain season	501	3	R579	8.6	-7.653968197	37.00216719	Low altitude	0

28	Dry season	501	9	R579	13.8	-7.653968197	37.00216719	Low altitude	0
29	Rain season	542	3	R570	8.8	-7.680921697	36.99772129	Low altitude	0
29	Dry season	542	9	R570	14	-7.680921697	36.99772129	Low altitude	0
30	Rain season	475	3	R570	4.2	-7.591007597	37.0032351	Low altitude	0
30	Dry season	475	9	R570	9.4	-7.591007597	37.0032351	Low altitude	0
31	Rain season	481	3	N41	4	-7.588808497	37.0092597	Low altitude	0
31	Dry season	481	9	N41	9.9	-7.588808497	37.0092597	Low altitude	0
32	Rain season	485	3	R570	4.2	-7.584436597	37.01136569	Low altitude	0
32	Dry season	485	9	R570	9.8	-7.584436597	37.01136569	Low altitude	0
33	Rain season	470	3	NCO376	4.6	-7.598869897	37.0095806	Low altitude	0
33	Dry season	470	9	NCO376	10	-7.598869897	37.0095806	Low altitude	0
34	Rain season	471	3	R570	5.8	-7.600777197	37.0136843	Low altitude	0
34	Dry season	471	9	R570	10.2	-7.600777197	37.0136843	Low altitude	1
35	Rain season	446	3	R579	8.4	-7.609356397	36.9997092	Low altitude	0
35	Dry season	446	9	R579	13.8	-7.609356397	36.9997092	Low altitude	0
36	Rain season	456	3	R579	7.6	-7.609100197	37.0099535	Low altitude	0
36	Dry season	456	9	R579	13	-7.609100197	37.0099535	Low altitude	0
37	Rain season	469	3	R570	5.1	-7.597036897	37.0054419	Low altitude	0
37	Dry season	469	9	R570	9.5	-7.597036897	37.0054419	Low altitude	0
38	Rain season	468	3	R570	6	-7.593238597	37.0038528	Low altitude	0
38	Dry season	468	9	R570	11.4	-7.593238597	37.0038528	Low altitude	0
39	Rain season	336	3	N36	9.3	-7.678014197	37.007917	Low altitude	0
39	Dry season	336	9	N36	14.7	-7.678014197	37.007917	Low altitude	0
40	Rain season	376	3	N36	9	-7.659906597	37.0228296	Low altitude	0
40	Dry season	376	9	N36	14.4	-7.659906597	37.0228296	Low altitude	0
41	Rain season	349	3	R579	3.6	-7.667041697	37.00432699	Low altitude	0
41	Dry season	349	9	R579	9.1	-7.667041697	37.00432699	Low altitude	0
42	Rain season	319	3	R579	4.1	-7.680605898	36.9882012	Low altitude	0
42	Dry season	319	9	R579	9.5	-7.680605898	36.9882012	Low altitude	0
43	Rain season	324	3	R579	4.5	-7.682252097	36.9897687	Low altitude	0
43	Dry season	324	9	R579	9.9	-7.682252097	36.9897687	Low altitude	1
44	Rain season	366	3	R579	4.9	-7.727963797	37.0138177	Low altitude	0
44	Dry season	366	9	R579	10.3	-7.727963797	37.0138177	Low altitude	0

45	Rain season	309	3	R579	7	-7.733701097	36.1063112	Low altitude	0
45	Dry season	309	9	R579	12.4	-7.733701097	36.1063112	Low altitude	0
46	Rain season	232	3	R579	8.3	-7.717963797	37.0038177	Low altitude	0
46	Dry season	232	9	R579	13.7	-7.717963797	37.0038177	Low altitude	0
47	Rain season	242	3	N36	9.2	-7.712648797	37.0116106	Low altitude	0
47	Dry season	242	9	N36	4	-7.712648797	37.0116106	Low altitude	0
48	Rain season	243	3	N25	4.4	-7.712788297	37.0116868	Low altitude	0
48	Dry season	243	9	N25	9.8	-7.712788297	37.0116868	Low altitude	0
49	Rain season	221	3	R579	4.7	-7.723701097	36.9963112	Low altitude	0
49	Dry season	221	9	R579	10.1	-7.723701097	36.9963112	Low altitude	0
50	Rain season	216	3	R579	7.3	-7.730496797	36.9956213	Low altitude	0
50	Dry season	216	9	R579	12.7	-7.730496797	36.9956213	Low altitude	0
51	Rain season	290	3	R570	6.5	-7.730563597	36.9955927	Low altitude	0
51	Dry season	290	9	R570	11.9	-7.730563597	36.9955927	Low altitude	0
52	Rain season	206	3	R579	7.4	-7.732178997	36.9850353	Low altitude	0
52	Dry season	206	9	R579	12.8	-7.732178997	36.9850353	Low altitude	0
53	Rain season	259	3	Mixed	4.1	-7.700142096	37.0234859	Low altitude	0
53	Dry season	259	9	Mixed	9.5	-7.700142096	37.0234859	Low altitude	0
54	Rain season	254	3	R579	4.1	-7.703385797	37.0219018	Low altitude	0
54	Dry season	254	9	R579	9.5	-7.703385797	37.0219018	Low altitude	0
55	Rain season	251	3	R579	7	-7.706552297	37.0184564	Low altitude	0
55	Dry season	251	9	R579	12.2	-7.706552297	37.0184564	Low altitude	0
56	Rain season	247	3	N25	5.1	-7.709697797	37.0149138	Low altitude	1
56	Dry season	247	9	N25	10.5	-7.709697797	37.0149138	Low altitude	1
57	Rain season	236	3	N36	9.4	-7.710097897	37.0037062	Low altitude	0
57	Dry season	236	9	N36	14.6	-7.710097897	37.0037062	Low altitude	1
58	Rain season	249	3	R579	8.6	-7.697599497	37.011896	Low altitude	0
58	Dry season	249	9	R579	14	-7.697599497	37.011896	Low altitude	0
59	Rain season	245	3	R579	4.2	-7.700475697	37.0081614	Low altitude	0
59	Dry season	245	9	R579	9.6	-7.700475697	37.0081614	Low altitude	1
60	Rain season	217	3	R579	7.2	-7.712062097	36.9849078	Low altitude	0
60	Dry season	217	9	R579	12.6	-7.712062097	36.9849078	Low altitude	0
61	Rain season	252	3	R579	7	-7.694426597	37.0144305	Low altitude	0

61	Dry season	252	9	R579	12.2	-7.694426597	37.0144305	Low altitude	0
62	Rain season	104	3	R570	5	-7.749589397	36.9858464	Low altitude	0
62	Dry season	104	9	R570	10.2	-7.749589397	36.9858464	Low altitude	0
63	Rain season	102	3	R570	3.7	-7.744757397	36.985115	Low altitude	0
63	Dry season	102	9	R570	8.9	-7.744757397	36.985115	Low altitude	0
64	Rain season	109	3	R579	3.7	-7.755892597	36.9983545	Low altitude	0
64	Dry season	109	9	R579	8.9	-7.755892597	36.9983545	Low altitude	0
65	Rain season	119	4	Mixed	8.4	-7.767551297	36.9913458	Low altitude	0
65	Dry season	119	9	Mixed	13.6	-7.767551297	36.9913458	Low altitude	0
66	Rain season	135	4	R570	7.1	-7.784433697	37.0009714	Low altitude	0
66	Dry season	135	9	R570	12.3	-7.784433697	37.0009714	Low altitude	0
67	Rain season	160	4	R570	4.3	-7.7952272	37.0166344	Low altitude	0
67	Dry season	160	9	R570	9.5	-7.7952272	37.0166344	Low altitude	0
68	Rain season	161	4	R570	4.3	-7.7951568	37.0167396	Low altitude	0
68	Dry season	161	9	R570	9.5	-7.7951568	37.0167396	Low altitude	0
69	Rain season	138	4	R570	6.5	-7.785917497	37.0130381	Low altitude	0
69	Dry season	138	9	R570	11.7	-7.785917497	37.0130381	Low altitude	0
70	Rain season	126	4	R570	6.4	-7.776832097	36.9932303	Low altitude	0
70	Dry season	126	9	R570	11.6	-7.776832097	36.9932303	Low altitude	0
1	Rain season	C9BA1	4	N25	11	-6.193721788	37.6463916	Low altitude	0
1	Dry season	C9BA1	8	N25	14.4	-6.193721788	37.6463916	Low altitude	0
2	Rain season	C3EA1	3	R579	4	-6.179437277	37.62252605	Low altitude	0
2	Dry season	C3EA1	8	R579	7.4	-6.179437277	37.62252605	Low altitude	0
3	Rain season	C2EA	3	R579	19	-6.179684527	37.61988115	Low altitude	0
3	Dry season	C2EA	8	R579	3.4	-6.179684527	37.61988115	Low altitude	0
4	Rain season	C3EB1	3	N25	12	-6.174128388	37.62474411	Low altitude	0
4	Dry season	C3EB1	8	N25	15.4	-6.174128388	37.62474411	Low altitude	0
5	Rain season	C4FA	3	R570	17.8	-6.172132177	37.62468796	Low altitude	0
5	Dry season	C4FA	8	R570	21.2	-6.172132177	37.62468796	Low altitude	0
6	Rain season	NM2A2	3	R579	6.1	-6.195848224	37.62081133	Low altitude	0
6	Dry season	NM2A2	8	R579	9.5	-6.195848224	37.62081133	Low altitude	0
7	Rain season	C2EB1	3	R570	19	-6.176705438	37.61964615	Low altitude	0
7	Dry season	C2EB1	8	R570	22.4	-6.176705438	37.61964615	Low altitude	0

8	Rain season	C2CB1	3	N25	12	-6.190789221	37.621137	Low altitude	0
8	Dry season	C2CB1	8	N25	15.4	-6.190789221	37.621137	Low altitude	0
9	Rain season	C2CA1	3	Mixed	8	-6.190570694	37.61586509	Low altitude	0
9	Dry season	C2CA1	8	Mixed	11.4	-6.190570694	37.61586509	Low altitude	0
10	Rain season	C1CB2	3	Mixed	6	-6.185813977	37.61529269	Low altitude	0
10	Dry season	C1CB2	8	Mixed	9.4	-6.185813977	37.61529269	Low altitude	0
11	Rain season	C1AA1	3	R570	6.4	-6.196246157	37.61204301	Low altitude	0
11	Dry season	C1AA1	8	R570	9.7	-6.196246157	37.61204301	Low altitude	0
12	Rain season	C4AA1	3	R570	14	-6.200107799	37.62700775	Low altitude	0
12	Dry season	C4AA1	8	R570	17.3	-6.200107799	37.62700775	Low altitude	0
13	Rain season	NM2A1	3	R579	6.1	-6.200929191	37.619312	Low altitude	0
13	Dry season	NM2A1	8	R579	9.4	-6.200929191	37.619312	Low altitude	0
14	Rain season	NM5A1	3	R579	8	-6.199886088	37.6320454	Low altitude	0
14	Dry season	NM5A1	8	R579	11.3	-6.199886088	37.6320454	Low altitude	0
15	Rain season	C3DA1	3	R579	17	-6.184980488	37.6204273	Low altitude	0
15	Dry season	C3DA1	8	R579	3.3	-6.184980488	37.6204273	Low altitude	0
16	Rain season	C4BA1	3	N25	7	-6.18961311	37.6262399	Low altitude	0
16	Dry season	C4BA1	8	N25	10.3	-6.18961311	37.6262399	Low altitude	0
17	Rain season	C5AB1	3	N41	4	-6.199675388	37.6358928	Low altitude	0
17	Dry season	C5AB1	8	N41	7.4	-6.199675388	37.6358928	Low altitude	0
18	Rain season	NK4A1	3	R579	8	-6.203498891	37.631868	Low altitude	0
18	Dry season	NK4A1	8	R579	11.4	-6.203498891	37.631868	Low altitude	0
19	Rain season	NK3A1	3	N25	4	-6.206099891	37.6269648	Low altitude	0
19	Dry season	NK3A1	8	N25	7.3	-6.206099891	37.6269648	Low altitude	0
20	Rain season	NK4B1	3	R570	8	-6.198800691	37.6359438	Low altitude	0
20	Dry season	NK4B1	8	R570	11.4	-6.198800691	37.6359438	Low altitude	0
21	Rain season	NL3A1	3	N25	18	-6.200724891	37.628923	Low altitude	0
21	Dry season	NL3A1	8	N25	4	-6.200724891	37.628923	Low altitude	0
22	Rain season	NL4B1	3	R579	18	-6.198786491	37.6340698	Low altitude	0
22	Dry season	NL4B1	8	R579	21.3	-6.198786491	37.6340698	Low altitude	0
23	Rain season	NL5B1	3	R579	5.2	-6.198869891	37.6406613	Low altitude	0
23	Dry season	NL5B1	8	R579	8.5	-6.198869891	37.6406613	Low altitude	0
24	Rain season	NI4B1	3	R579	7	-6.208369791	37.6392124	Low altitude	0

24	Dry season	NI4B1	8	R579	11	-6.208369791	37.6392124	Low altitude	0
25	Rain season	NI5B	3	R579	7.1	-6.238254791	37.67156837	Low altitude	0
25	Dry season	NI5B	8	R579	11.1	-6.238254791	37.67156837	Low altitude	0
26	Rain season	NJ4B1	3	N25	7	-6.208254791	37.65156837	Low altitude	0
26	Dry season	NJ4B1	8	N25	11	-6.208254791	37.65156837	Low altitude	0
27	Rain season	NJ3B1	3	R579	17	-6.205901491	37.6317975	Low altitude	0
27	Dry season	NJ3B1	8	R579	4	-6.205901491	37.6317975	Low altitude	0
28	Rain season	C3BA1	3	R579	5	-6.190059388	37.6233503	Low altitude	0
28	Dry season	C3BA1	8	R579	9	-6.190059388	37.6233503	Low altitude	0
29	Rain season	S16BB	4	N25	7	-6.216038936	37.68299	Low altitude	0
29	Dry season	S16BB	8	N25	11	-6.216038936	37.68299	Low altitude	0
30	Rain season	S15DB	4	R579	5	-6.206037213	37.6794968	Low altitude	0
30	Dry season	S15DB	8	R579	9	-6.206037213	37.6794968	Low altitude	0
31	Rain season	S16EA	4	R579	13	-6.20190048	37.6791662	Low altitude	0
31	Dry season	S16EA	8	R579	17	-6.20190048	37.6791662	Low altitude	0
32	Rain season	S16EB	4	N41	15.1	-6.20175098	37.6817633	Low altitude	0
32	Dry season	S16EB	8	N41	19.1	-6.20175098	37.6817633	Low altitude	0
33	Rain season	S15EB	4	R579	7.8	-6.20207198	37.6765564	Low altitude	0
33	Dry season	S15EB	8	R579	11.8	-6.20207198	37.6765564	Low altitude	0
34	Rain season	S15FA	4	N41	4.2	-6.19270388	37.6732337	Low altitude	0
34	Dry season	S15FA	8	N41	8.2	-6.19270388	37.6732337	Low altitude	0
35	Rain season	S14AB	4	N41	4.3	-6.196149288	37.6807056	Low altitude	0
35	Dry season	S14AB	8	N41	8.3	-6.196149288	37.6807056	Low altitude	0
36	Rain season	S15CB	4	N41	17.4	-6.21160588	37.6798467	Low altitude	0
36	Dry season	S15CB	8	N41	4	-6.21160588	37.6798467	Low altitude	0
37	Rain season	S14HB	4	N41	9	-6.160543187	37.6638733	Low altitude	0
37	Dry season	S14HB	8	N41	13	-6.160543187	37.6638733	Low altitude	0
38	Rain season	S14GA	4	R579	6.1	-6.160726187	37.662675	Low altitude	0
38	Dry season	S14GA	8	R579	10.1	-6.160726187	37.662675	Low altitude	0
39	Rain season	S14HA	4	N25	7.6	-6.160663287	37.6609536	Low altitude	0
39	Dry season	S14HA	8	N25	11.6	-6.160663287	37.6609536	Low altitude	0
40	Rain season	S13GB	4	N41	16.4	-6.160754587	37.6608489	Low altitude	0
40	Dry season	S13GB	8	N41	20.4	-6.160754587	37.6608489	Low altitude	0

41	Rain season	S13GA	4	R579	8	-6.165944587	37.656105	Low altitude	0
41	Dry season	S13GA	8	R579	12	-6.165944587	37.656105	Low altitude	0
42	Rain season	S13HA	4	N41	4	-6.161074587	37.6558035	Low altitude	0
42	Dry season	S13HA	8	N41	8	-6.161074587	37.6558035	Low altitude	0
43	Rain season	S13HB	4	Mixed	12.1	-6.160780887	37.6583291	Low altitude	0
43	Dry season	S13HB	8	Mixed	16.1	-6.160780887	37.6583291	Low altitude	0
44	Rain season	S14DA	4	Mixed	15	-6.202363102	37.6738567	Low altitude	0
44	Dry season	S14DA	8	Mixed	19	-6.202363102	37.6738567	Low altitude	0
45	Rain season	S14EB	4	R579	15.5	-6.20249038	37.6713488	Low altitude	0
45	Dry season	S14EB	8	R579	19.5	-6.20249038	37.6713488	Low altitude	0
46	Rain season	S14CB	4	R570	17.8	-6.21142938	37.6736814	Low altitude	0
46	Dry season	S14CB	8	R570	21.8	-6.21142938	37.6736814	Low altitude	0
47	Rain season	S13AA	4	N12	9	-6.196573488	37.6727303	Low altitude	0
47	Dry season	S13AA	8	N12	13	-6.196573488	37.6727303	Low altitude	0
48	Rain season	S14CA	4	R579	15.2	-6.21142938	37.6736814	Low altitude	0
48	Dry season	S14CA	8	R579	19.2	-6.21142938	37.6736814	Low altitude	0
49	Rain season	S13CB	4	N25	12.3	-6.21190158	37.6694332	Low altitude	0
49	Dry season	S13CB	8	N25	16.3	-6.21190158	37.6694332	Low altitude	0
50	Rain season	S13CA	4	R579	15.4	-6.204227902	37.6664544	Low altitude	0
50	Dry season	S13CA	8	R579	19.4	-6.204227902	37.6664544	Low altitude	0
51	Rain season	S12CA	4	N41	8.6	-6.182862087	37.6575863	Low altitude	0
51	Dry season	S12CA	8	N41	12	-6.182862087	37.6575863	Low altitude	0
52	Rain season	S12DA	4	N25	14.7	-6.177720587	37.6571555	Low altitude	0
52	Dry season	S12DA	8	N25	18.7	-6.177720587	37.6571555	Low altitude	0
53	Rain season	S12AB	4	R579	4.9	-6.21801868	37.6647288	Low altitude	0
53	Dry season	S12AB	8	R579	8.9	-6.21801868	37.6647288	Low altitude	0
54	Rain season	S11HA	4	N25	5.3	-6.155241588	37.6607499	Low altitude	0
54	Dry season	S11HA	8	N25	9.3	-6.155241588	37.6607499	Low altitude	0
55	Rain season	S11AA	4	N41	12.1	-6.197771721	37.6631637	Low altitude	0
55	Dry season	S11AA	8	N41	16.1	-6.197771721	37.6631637	Low altitude	0
56	Rain season	S11AB	4	R579	5	-6.21810068	37.659632	Low altitude	0
56	Dry season	S11AB	8	R579	9	-6.21810068	37.659632	Low altitude	0
57	Rain season	S11HB	4	R570	6.3	-6.155196188	37.64542643	Low altitude	1

57	Dry season	S11HB	8	R570	10.3	-6.155196188	37.64542643	Low altitude	0
58	Rain season	S12GA	4	Mixed	18.3	-6.161232287	37.655707	Low altitude	0
58	Dry season	S12GA	8	Mixed	22.3	-6.161232287	37.655707	Low altitude	0
59	Rain season	S11FA	4	R579	14.9	-6.172160087	37.6540659	Low altitude	0
59	Dry season	S11FA	8	R579	18.9	-6.172160087	37.6540659	Low altitude	0
60	Rain season	S11EB	4	R570	15	-6.169917432	37.67914	Low altitude	0
60	Dry season	S11EB	8	R570	19	-6.169917432	37.67914	Low altitude	0
61	Rain season	S11KA	4	R579	9.3	-6.149917432	37.65914	Low altitude	0
61	Dry season	S11KA	8	R579	13.3	-6.149917432	37.65914	Low altitude	0
62	Rain season	S12EA	4	N12	18	-6.172136187	37.6568697	Low altitude	0
62	Dry season	S12EA	8	N12	4	-6.172136187	37.6568697	Low altitude	0
63	Rain season	S12FB	4	N25	8.4	-6.166559587	37.65612122	Low altitude	0
63	Dry season	S12FB	8	N25	16.4	-6.166559587	37.65612122	Low altitude	0
64	Rain season	S12EB	4	Mixed	15.2	-6.20281728	37.6688026	Low altitude	0
64	Dry season	S12EB	8	Mixed	19.2	-6.20281728	37.6688026	Low altitude	0
65	Rain season	S12IB	4	R579	14.5	-6.19656768	37.66300828	Low altitude	0
65	Dry season	S12IB	8	R579	18.5	-6.19656768	37.66300828	Low altitude	0
66	Rain season	S13DA	4	N25	12.3	-6.20376388	37.6636925	Low altitude	0
66	Dry season	S13DA	8	N25	16.3	-6.20376388	37.6636925	Low altitude	0
67	Rain season	C9BA	4	R579	8	-6.166351088	37.6564009	Low altitude	0
67	Dry season	C9BA	8	R579	12	-6.166351088	37.6564009	Low altitude	0
68	Rain season	C9BB	4	N25	10.9	-6.192811444	37.6571387	Low altitude	0
68	Dry season	C9BB	8	N25	14.9	-6.192811444	37.6571387	Low altitude	0
69	Rain season	C9FB1	4	N25	6.7	-6.166386385	37.6550478	Low altitude	0
69	Dry season	C9FB1	8	N25	10.7	-6.166386385	37.6550478	Low altitude	0
70	Rain season	C10EA1	4	N25	7	-6.172605688	37.6556051	Low altitude	0
70	Dry season	C10EA1	8	N25	11	-6.172605688	37.6556051	Low altitude	0
71	Rain season	C9AA1	4	N41	4.5	-6.198499588	37.6525817	Low altitude	0
71	Dry season	C9AA1	8	N41	8.5	-6.198499588	37.6525817	Low altitude	0
72	Rain season	C9CA1	4	N25	4.8	-6.182402588	37.6537953	Low altitude	0
72	Dry season	C9CA1	8	N25	8.8	-6.182402588	37.6537953	Low altitude	0
73	Rain season	C9CB	4	N41	5	-6.182292877	37.6563521	Low altitude	0
73	Dry season	C9CB	8	N41	9	-6.182292877	37.6563521	Low altitude	0

74	Rain season	C9AB	4	Mixed	5.1	-6.192811444	37.6571387	Low altitude	0
74	Dry season	C9AB	8	Mixed	9.1	-6.192811444	37.6571387	Low altitude	0
75	Rain season	C10HB1	4	N12	16.3	-6.155664388	37.6569632	Low altitude	0
75	Dry season	C10HB1	8	N12	4	-6.155664388	37.6569632	Low altitude	0
76	Rain season	C9DB1	4	N12	14	-6.182579588	37.6514461	Low altitude	0
76	Dry season	C9DB1	8	N12	18	-6.182579588	37.6514461	Low altitude	0
77	Rain season	C9EB1	4	R579	8.8	-6.171669888	37.6554384	Low altitude	0
77	Dry season	C9EB1	8	R579	12.8	-6.171669888	37.6554384	Low altitude	0
78	Rain season	S16CB	4	N41	17.3	-6.211311686	37.6824601	Low altitude	0
78	Dry season	S16CB	8	N41	4	-6.211311686	37.6824601	Low altitude	0
79	Rain season	S13JB	4	Mixed	15	-6.149908088	37.6616104	Low altitude	0
79	Dry season	S13JB	8	Mixed	19	-6.149908088	37.6616104	Low altitude	0
80	Rain season	S16CA	4	N41	17.9	-6.151021021	37.6564356	Low altitude	0
80	Dry season	S16CA	8	N41	4	-6.151021021	37.6564356	Low altitude	0
81	Rain season	C10IA1	4	N41	5.8	-6.154857088	37.6568758	Low altitude	0
81	Dry season	C10IA1	8	N41	9.8	-6.154857088	37.6568758	Low altitude	0
82	Rain season	C10EB1	4	N25	13.8	-6.171447288	37.6582773	Low altitude	0
82	Dry season	C10EB1	8	N25	17.8	-6.171447288	37.6582773	Low altitude	0
83	Rain season	C7FA1	4	N12	10.4	-6.170802788	37.6400799	Low altitude	0
83	Dry season	C7FA1	8	N12	14.4	-6.170802788	37.6400799	Low altitude	0
84	Rain season	C6FB	4	N41	7.2	-6.166062088	37.6395817	Low altitude	0
84	Dry season	C6FB	8	N41	11.2	-6.166062088	37.6395817	Low altitude	0
85	Rain season	C8JA1	4	N25	6.2	-6.148114488	37.6435105	Low altitude	0
85	Dry season	C8JA1	8	N25	10.2	-6.148114488	37.6435105	Low altitude	0
86	Rain season	C7JB1	4	N41	8.2	-6.151159421	37.6438512	Low altitude	0
86	Dry season	C7JB1	8	N41	12.2	-6.151159421	37.6438512	Low altitude	0
87	Rain season	C9IA1	4	N12	7	-6.155982588	37.65399092	Low altitude	0
87	Dry season	C9IA1	8	N12	11	-6.155982588	37.65399092	Low altitude	0
88	Rain season	C91HB1	4	N25	16	-6.156111588	37.6518765	Low altitude	0
88	Dry season	C91HB1	8	N25	4	-6.156111588	37.6518765	Low altitude	0
89	Rain season	C8HB1	4	N41	8	-6.156463301	37.64725766	Low altitude	0
89	Dry season	C8HB1	8	N41	12	-6.156463301	37.64725766	Low altitude	0
90	Rain season	C9HA1	4	N25	8	-6.156265888	37.6493337	Low altitude	1

90	Dry season	C9HA1	8	N25	12	-6.156265888	37.6493337	Low altitude	1
91	Rain season	C6AB1	4	N25	6.8	-6.199469488	37.6422006	Low altitude	0
91	Dry season	C6AB1	8	N25	10.8	-6.199469488	37.6422006	Low altitude	0
92	Rain season	C6BB1	4	N25	7	-6.193905788	37.6417669	Low altitude	1
92	Dry season	C6BB1	8	N25	11	-6.193905788	37.6417669	Low altitude	1
93	Rain season	C7AA1	4	R579	11	-6.193962588	37.6420537	Low altitude	0
93	Dry season	C7AA1	8	R579	14	-6.193962588	37.6420537	Low altitude	0
94	Rain season	C7BB1	4	N12	11.3	-6.193614188	37.6444337	Low altitude	0
94	Dry season	C7BB1	8	N12	15.3	-6.193614188	37.6444337	Low altitude	0
95	Rain season	NM10A1	4	R579	7.6	-6.197537991	37.6572907	Low altitude	0
95	Dry season	NM10A1	8	R579	11.6	-6.197537991	37.6572907	Low altitude	0
96	Rain season	S14KA	4	N25	7.8	-6.146172387	37.6598849	Low altitude	0
96	Dry season	S14KA	8	N25	11.8	-6.146172387	37.6598849	Low altitude	0
97	Rain season	S14JA	4	R579	17.3	-6.151010587	37.6600719	Low altitude	0
97	Dry season	S14JA	8	R579	4	-6.151010587	37.6600719	Low altitude	0
98	Rain season	NM7A1	4	N25	12.1	-6.193563191	37.657003	Low altitude	0
98	Dry season	NM7A1	8	N25	16.1	-6.193563191	37.657003	Low altitude	0
99	Rain season	NJ7A1	4	R579	12	-6.198219591	37.6494469	Low altitude	0
99	Dry season	NJ7A1	8	R579	16	-6.198219591	37.6494469	Low altitude	0
100	Rain season	NL7A1	4	R570	15.3	-6.205785091	37.6475849	Low altitude	0
100	Dry season	NL7A1	8	R570	19.3	-6.205785091	37.6475849	Low altitude	0
101	Rain season	NL9A1	4	N41	6.5	-6.198218491	37.649462	Low altitude	0
101	Dry season	NL9A1	8	N41	10.5	-6.198218491	37.649462	Low altitude	0
102	Rain season	NM8B1	4	R579	8.6	-6.198866388	37.6525954	Low altitude	0
102	Dry season	NM8B1	8	R579	12.6	-6.198866388	37.6525954	Low altitude	0
103	Rain season	NL8B1	4	N12	6.7	-6.203500888	37.6580403	Low altitude	0
103	Dry season	NL8B1	8	N12	10.7	-6.203500888	37.6580403	Low altitude	0
104	Rain season	NM8B1	4	R579	8.6	-6.193701191	37.6464192	Low altitude	0
104	Dry season	NM8B1	8	R579	12.6	-6.193701191	37.6464192	Low altitude	0
105	Rain season	S14IB	4	R579	7.2	-6.155593143	37.6657863	Low altitude	0
105	Dry season	S14IB	8	R579	11.2	-6.155593143	37.6657863	Low altitude	0
106	Rain season	S14DB	4	R579	17.4	-6.20245838	37.67392907	Low altitude	0
106	Dry season	S14DB	8	R579	4	-6.20245838	37.67392907	Low altitude	0

107	Rain season	C10JA	4	N25	16.7	-6.203750888	37.6555291	Low altitude	0
107	Dry season	C10JA	8	N25	4	-6.203750888	37.6555291	Low altitude	0
108	Rain season	NB5A1	4	R579	8.2	-6.236686288	37.660726	Low altitude	0
108	Dry season	NB5A1	8	R579	12.2	-6.236686288	37.660726	Low altitude	0
109	Rain season	NM9A1	4	N12	4	-6.20206328	37.6765633	Low altitude	0
109	Dry season	NM9A1	8	N12	8	-6.20206328	37.6765633	Low altitude	0
110	Rain season	NJ6A1	4	N41	5.2	-6.206149591	37.6443778	Low altitude	0
110	Dry season	NJ6A1	8	N41	9.2	-6.206149591	37.6443778	Low altitude	0
111	Rain season	NA3A	4	R579	6.9	-6.231801588	37.6601731	Low altitude	0
111	Dry season	NA3A	8	R579	10.9	-6.231801588	37.6601731	Low altitude	0
112	Rain season	NA4B1	4	N25	6.8	-6.231739588	37.6678103	Low altitude	0
112	Dry season	NA4B1	8	N25	10.8	-6.231739588	37.6678103	Low altitude	0
113	Rain season	NF1A1	4	R579	4.8	-6.211683532	37.6596822	Low altitude	0
113	Dry season	NF1A1	8	R579	8.8	-6.211683532	37.6596822	Low altitude	0
114	Rain season	NE1A	4	N25	7.4	-6.209471288	37.6564587	Low altitude	0
114	Dry season	NE1A	8	N25	11.4	-6.209471288	37.6564587	Low altitude	0
115	Rain season	NA1A1	4	R579	5.5	-6.222994332	37.6760301	Low altitude	0
115	Dry season	NA1A1	8	R579	9.5	-6.222994332	37.6760301	Low altitude	0
116	Rain season	NA4A1	4	N25	6.4	-6.2423239	37.6730097	Low altitude	0
116	Dry season	NA4A1	8	N25	10.4	-6.2423239	37.6743167	Low altitude	0
117	Rain season	NA2A1	4	R579	5.3	-6.226489488	37.6735889	Low altitude	0
117	Dry season	NA2A1	8	R579	9.3	-6.226489488	37.6735889	Low altitude	0
118	Rain season	NB3B1	4	R579	6.8	-6.229106588	37.6635409	Low altitude	0
118	Dry season	NB3B1	8	R579	10.8	-6.229106588	37.6635409	Low altitude	0
119	Rain season	NA1B1	4	R579	5.5	-6.223285588	37.6731568	Low altitude	0
119	Dry season	NA1B1	8	R579	9.5	-6.223285588	37.6731568	Low altitude	0
120	Rain season	NB6A1	4	N25	6.4	-6.242450888	37.6629989	Low altitude	0
120	Dry season	NB6A1	8	N25	10.4	-6.242450888	37.6629989	Low altitude	0
121	Rain season	NA6B1	4	R579	6.6	-6.238352888	37.6633796	Low altitude	0
121	Dry season	NA6B1	8	R579	10.6	-6.238352888	37.6633796	Low altitude	0
122	Rain season	NB5A1	4	N12	8.2	-6.236686288	37.660726	Low altitude	0
122	Dry season	NB5A1	8	N12	12.2	-6.236686288	37.660726	Low altitude	0
123	Rain season	NB6B1	4	N12	12.8	-6.236674966	37.66025758	Low altitude	0

123	Dry season	NB6B1	8	N12	16.8	-6.236674966	37.66025758	Low altitude	0
124	Rain season	NC2A1	4	N25	6.5	-6.224552488	37.6635903	Low altitude	0
124	Dry season	NC2A1	8	N25	10.5	-6.224552488	37.6635903	Low altitude	0
125	Rain season	ND4A1	4	R570	13.6	-6.225389305	37.6630552	Low altitude	0
125	Dry season	ND4A1	8	R570	17.6	-6.225389305	37.6630552	Low altitude	0
126	Rain season	NC3A1	4	R579	6.8	-6.227752788	37.6614693	Low altitude	0
126	Dry season	NC3A1	8	R579	10.8	-6.227752788	37.6614693	Low altitude	0
127	Rain season	NC4A1	4	N41	13.7	-6.232466788	37.65833029	Low altitude	0
127	Dry season	NC4A1	8	N41	17.7	-6.232466788	37.65833029	Low altitude	0
128	Rain season	NB2B1	4	R579	5.3	-6.221467388	37.6655969	Low altitude	0
128	Dry season	NB2B1	8	R579	9.3	-6.221467388	37.6655969	Low altitude	0
129	Rain season	NF4A1	4	R570	14.3	-6.228115588	37.6505447	Low altitude	0
129	Dry season	NF4A1	8	R570	18.3	-6.228115588	37.6505447	Low altitude	0
130	Rain season	ND4B1	4	N12	9.6	-6.229898988	37.6545181	Low altitude	0
130	Dry season	ND4B1	8	N12	13.6	-6.229898988	37.6545181	Low altitude	0
131	Rain season	NB2B1	4	R579	5.3	-6.226655788	37.6650694	Low altitude	0
131	Dry season	NB2B1	8	R579	9.3	-6.226655788	37.6650694	Low altitude	0
132	Rain season	NB1B1	4	R579	5.6	-6.226648488	37.6650654	Low altitude	0
132	Dry season	NB1B1	8	R579	9.6	-6.226648488	37.6650654	Low altitude	0
133	Rain season	NA2B1	4	R579	5.4	-6.218097288	37.6673224	Low altitude	0
133	Dry season	NA2B1	8	R579	9.4	-6.218097288	37.6673224	Low altitude	0
134	Rain season	NB1B1	4	R579	5.6	-6.224016388	37.6696484	Low altitude	0
134	Dry season	NB1B1	8	R579	9.6	-6.224016388	37.6696484	Low altitude	0
135	Rain season	NB1A1	4	R579	5.6	-6.222685288	37.6676374	Low altitude	0
135	Dry season	NB1A1	8	R579	9.6	-6.222685288	37.6676374	Low altitude	0
136	Rain season	NB3B1	4	R579	6.8	-6.229106594	37.6635409	Low altitude	0
136	Dry season	NB3B1	8	R579	10.8	-6.229106594	37.6635409	Low altitude	0
137	Rain season	NI7A1	4	R579	7.6	-6.212164891	37.648122	Low altitude	0
137	Dry season	NI7A1	8	R579	11.6	-6.212164891	37.648122	Low altitude	0
138	Rain season	N16A1	4	R570	15.3	-6.207828191	37.645923	Low altitude	0
138	Dry season	N16A1	8	R570	19.3	-6.207828191	37.645923	Low altitude	0
139	Rain season	NJ5A1	4	R579	19.2	-6.207995291	37.6414008	Low altitude	0
139	Dry season	NJ5A1	8	R579	4	-6.207995291	37.6414008	Low altitude	0

140	Rain season	NF8A1	4	R570	15.7	-6.241536088	37.6399673	Low altitude	0
140	Dry season	NF8A1	8	R570	19.7	-6.241536088	37.6399673	Low altitude	0
141	Rain season	NE6A1	4	N25	8.6	-6.233401594	37.6452517	Low altitude	0
141	Dry season	NE6A1	8	N25	12.6	-6.233401594	37.6452517	Low altitude	0
