# Supplementary material for: Factors affecting the infestation status of African sugarcane stalk borer (Eldana saccharina Walker) (Lepidoptera: Pyralidae) in Tanzania
Source: PLoS One. 2026 Aug 31;21(8):e0355844. doi: 10.1371/journal.pone.0355844 (PMC13528964; doi:10.1371/journal.pone.0355844)
Supplement: Supplementary file 2 [file pone.0355844.s002.pdf]

Sampling site	Variety	Number of block-season observations	Number of unique blocks	Stalks assessed	Total site determinations	Percent of site determinations
Low altitude	R579	164	78	8200	422	38.86
Low altitude	R570	78	39	3900	422	18.48
Low altitude	N25	72	36	3600	422	17.06
Low altitude	N41	46	23	2300	422	10.9
Low altitude	N12	24	12	1200	422	5.69
Low altitude	Mixed	20	10	1000	422	4.74
Low altitude	N36	10	5	500	422	2.37
Low altitude	MN1	2	1	100	422	0.47
Low altitude	N19	2	1	100	422	0.47
Low altitude	NCO376	2	1	100	422	0.47
Low altitude	R759	2	1	100	422	0.47
Medium altitude	N25	120	61	6000	350	34.29
Medium altitude	R579	104	51	5200	350	29.71
Medium altitude	KQ228	20	10	1000	350	5.71
Medium altitude	M381/95	18	10	900	350	5.14
Medium altitude	R575	18	9	900	350	5.14
Medium altitude	N30	16	8	800	350	4.57
Medium altitude	M2256/88	12	6	600	350	3.43
Medium altitude	Mixed	10	5	500	350	2.86
Medium altitude	R585	10	5	500	350	2.86
Medium altitude	R94/6113	10	5	500	350	2.86
Medium altitude	R580	8	4	400	350	2.29
Medium altitude	M225/88	2	1	100	350	0.57
Medium altitude	M700/86	2	1	100	350	0.57
High altitude	N47	194	95	9700	650	29.85

High altitude	N41	166	80	8300	650	25.54
High altitude	N49	112	56	5600	650	17.23
High altitude	N25	86	43	4300	650	13.23
High altitude	R579	44	21	2200	650	6.77
High altitude	Mixed	19	10	950	650	2.92
High altitude	CO617	10	5	500	650	1.54
High altitude	N46	10	5	500	650	1.54
High altitude	R570	4	2	200	650	0.62
High altitude	MN1	2	1	100	650	0.31
High altitude	R92/6545	2	1	100	650	0.31
High altitude	KS6C	1	1	50	650	0.15
