# Supplementary material for: Factors affecting the infestation status of African sugarcane stalk borer (Eldana saccharina Walker) (Lepidoptera: Pyralidae) in Tanzania
Source: PLoS One. 2026 Aug 31;21(8):e0355844. doi: 10.1371/journal.pone.0355844 (PMC13528964; doi:10.1371/journal.pone.0355844)
Supplement: Supplementary file 3 [file pone.0355844.s003.pdf]

PLOS One

Factors affecting the infestation status of African sugarcane stalk borer (Eldana saccharina Walker) (Lepidoptera: Pyralidae) in Tanzania

--Manuscript Draft--

**Manuscript Number:**	PONE-D-26-23049
**Article Type:**	Research Article
**Full Title:**	Factors affecting the infestation status of African sugarcane stalk borer (Eldana saccharina Walker) (Lepidoptera: Pyralidae) in Tanzania
**Short Title:**	Infestation status of African sugarcane stalk borer (Eldana saccharina Walker) in Tanzania
**Corresponding Author:**	Hamis Daniel Wambura, M. Sc<br>Mwalimu Nyerere University of Agriculture and Technology<br>Mara, Musoma TANZANIA, UNITED REPUBLIC OF
**Keywords:**	Altitudes, crop age, Eldana saccharina, incidence, seasons, sugarcane varieties, agroecological zones
**Abstract:**	Sugarcane is a commercially important crop that contributes substantially to sugar production globally and across Sub-Saharan Africa. However, its productivity is constrained by stem borer infestations, particularly Eldana saccharina. The pest reduces sugarcane yield by up to 30% and about 18% of sucrose content is compromised. Nonetheless, the influence of ecological parameter on its pest status in Tanzania was poorly understood. This study, therefore, evaluated the influence of season, altitude, crop age, and sugarcane variety on E. saccharina infestation across different altitudes. A stratified multistage cross-sectional survey was conducted and samples were collected from randomly selected stalks. E. saccharina infestation was assessed using larvae per 50 stalks alongside other damage parameters. Data were analyzed using generalized linear mixed models in R, with likelihood ratio tests and tests. E. saccharina infestation was highest at high altitude in rainy and dry seasons and the infestation levels were significantly) influenced by the interaction of season, altitude, and crop ages. Internal stalk tissue damage was significantly greater at high altitude during the rainy season Boring intensity was also significantly higher in medium and high altitudes during the rainy season (respectively), with the bottom part of the stem being most affected Blocks planted with mixed varieties and cultivars N41 and N49 showed greater susceptibility to infestation. Therefore, ecological parameters exert influence on E. saccharina infestation in Tanzania hence should be considered in strategic management of the pest.
**Order of Authors:**	Hamis Daniel Wambura, M. Sc
	Gration Mutashoberwa Rwegasira, Professor
	Martin John Martin, Ph. D
**Additional Information:**	
**Question**	**Response**
**Financial Disclosure**<br><br>Enter a financial disclosure statement that describes the sources of funding for the work included in this submission. Review the submission guidelines for detailed requirements. View published research articles from *PLOS ONE* for specific examples.	The author(s) received no specific funding for this work.

Powered by Editorial Manager® and ProduXion Manager® from Aries Systems Corporation

This statement is required for submission and **will appear in the published article** if the submission is accepted. Please make sure it is accurate.<br><br>**Funded studies**<br>Enter a statement with the following details:<br>• Initials of the authors who received each award<br>• Grant numbers awarded to each author<br>• The full name of each funder<br>• URL of each funder website<br>• Did the sponsors or funders play any role in the study design, data collection and analysis, decision to publish, or preparation of the manuscript?<br><br>Did you receive funding for this work?	
**Competing Interests**<br><br>Use the instructions below to enter a competing interest statement for this submission. On behalf of all authors, disclose any competing interests that could be perceived to bias this work—acknowledging all financial support and any other relevant financial or non-financial competing interests.<br><br>This statement is required for submission and **will appear in the published article** if the submission is accepted. Please make sure it is accurate and that any funding sources listed in your Funding Information later in the submission form are also declared in your Financial Disclosure statement.<br><br>View published research articles from *PLOS ONE* for specific examples.	The authors have declared that no competing interests exist.

Powered by Editorial Manager® and ProduXion Manager® from Aries Systems Corporation

**NO authors have competing interests**<br><br>Enter: *The authors have declared that no competing interests exist.*<br><br>**Authors with competing interests**<br><br>Enter competing interest details beginning with this statement:<br><br>*I have read the journal's policy and the authors of this manuscript have the following competing interests: [insert competing interests here]*<br><br>* typeset	
**Ethics Statement**<br><br>Enter an ethics statement for this submission. This statement is required if the study involved:<br><br>• Human participants<br>• Human specimens or tissue<br>• Vertebrate animals or cephalopods<br>• Vertebrate embryos or tissues<br>• Field research<br><br>Write "N/A" if the submission does not require an ethics statement.<br><br>General guidance is provided below. Consult the submission guidelines for detailed instructions. **Make sure that all information entered here is included in the Methods section of the manuscript.**	This study methodology was approved by the Ethics Review Board of the College of Agriculture, Sokoine University of Agriculture, Tanzania (Approval Number: SUA/DPRTC/PCS/D/2022/0003/04). Additionally, Sugarcane-producing companies including Kagera Sugar Company Limited, Tanganyika Planting Company Limited (TPC), Kilombero Sugar Company Limited, and Mtibwa Sugar Estates granted a permision to use their estates for this study.

Powered by Editorial Manager® and ProduXion Manager® from Aries Systems Corporation

**Format for specific study types**<br><br>**Human Subject Research (involving human participants and/or tissue)**<br>• Give the name of the institutional review board or ethics committee that approved the study<br>• Include the approval number and/or a statement indicating approval of this research<br>• Indicate the form of consent obtained (written/oral) or the reason that consent was not obtained (e.g. the data were analyzed anonymously)<br><br>**Animal Research (involving vertebrate animals, embryos or tissues)**<br>• Provide the name of the Institutional Animal Care and Use Committee (IACUC) or other relevant ethics board that reviewed the study protocol, and indicate whether they approved this research or granted a formal waiver of ethical approval<br>• Include an approval number if one was obtained<br>• If the study involved *non-human primates*, add *additional details* about animal welfare and steps taken to ameliorate suffering<br>• If anesthesia, euthanasia, or any kind of animal sacrifice is part of the study, include briefly which substances and/or methods were applied<br><br>**Field Research**<br><br>Include the following details if this study involves the collection of plant, animal, or other materials from a natural setting:<br>• Field permit number<br>• Name of the institution or relevant body that granted permission	
Data Availability<br><br>Before publication, Authors are required to make fully available and without restriction all data underlying their findings. Sharing the data underlying your findings increases the visibility, credibility, and impact of your work, while supporting transparency and future scientific discovery. Please see our PLOS Data Policy page for detailed information on this policy.</p><br>A Data Availability	All relevant data are within the manuscript as Supporting Information files

Statement, detailing where the data can be accessed, is required at first submission. Insert your Data Availability Statement in the box below.</p><br><br>Please see the data reporting section of our submission guidelines for instructions on what you need to include in your Data Availability Statement. Examples of acceptable Data Availability Statements are included here for reference:</p><br> <br> - The minimal data set [and accompanying code, where generated] is available at [repository name] via [DOI hyperlink] <br> - No data was generated by this study. The following existing data sources were used: [dataset name] from [dataset location] available via [web address or DOI hyperlink]. <br> - For Study Protocols: No datasets were generated or analysed during the current study. All relevant data from this study will be made available upon study completion. <br> <br><br><i>PLOS allows rare exemptions to address legal and ethical concerns. If you have legal or ethical restrictions, please use the box below to detail these in full sentences for the Journal team to consider.</i></p><br><br>This statement will be typeset if the manuscript is accepted for publication.</p>	

Manuscript Click here to access/download;Manuscript;Manuscript.docx

Factors affecting the infestation status of African sugarcane stalk borer (*Eldana saccharina* Walker) (Lepidoptera: Pyralidae) in Tanzania

Hamis Daniel Wambura[1,2*], Gration Mutashoberwa Rwegasira[2], and Martin John Martin[3]

[1]Mwalimu Nyerere University of Agriculture and Technology, P.O. Box 976, Butiama, Tanzania.

[2]Department of Crop Science and Horticulture, Sokoine University of Agriculture, P.O. Box 3005, Morogoro, Tanzania

[3]Institute of Pest Management, Sokoine University of Agriculture, P. O Box 3110, Morogoro, Tanzania.

***Correspondence:** hamisdaniel@gmail.com (HDW)

Abstract

Sugarcane is a commercially important crop that contributes substantially to sugar production globally and across Sub-Saharan Africa. However, its productivity is constrained by stem borer infestations, particularly *Eldana saccharina.* The pest reduces sugarcane yield by up to 30% and about 18% of sucrose content is compromised. Nonetheless, the influence of ecological parameter on its pest status in Tanzania was poorly understood. This study, therefore, evaluated the influence of season, altitude, crop age, and sugarcane variety on *E. saccharina* infestation ~~across different altitudes~~. A stratified multistage cross-sectional survey was conducted and samples were collected from randomly selected stalks. *E. saccharina* infestation was assessed using larvae per 50 stalks alongside other damage parameters. Data were analyzed using generalized linear mixed models in R, with likelihood ratio tests and $Type\ III\ Wald\ x^2$ tests. *E. saccharina* infestation was highest at high altitude in rainy and dry seasons and the infestation levels were

significantly ($x^2 = 9.58, \mathrm{P} < 0.0083$) influenced by the interaction of season, altitude, and crop ages. Internal stalk tissue damage was significantly greater at high altitude during the rainy season ($\mathrm{P} = 0.04$). Boring intensity was also significantly higher in medium and high altitudes during the rainy season ($\mathrm{P} = 0.013; \mathrm{P} = 0.004$, respectively), with the bottom part of the stem being most affected ($\mathrm{P} = 0.001$). Blocks planted with mixed varieties and cultivars N41 and N49 showed greater susceptibility to infestation. Therefore, ecological parameters exert influence on *E. saccharina* infestation in Tanzania hence should be considered in strategic management of the pest.

Key words

Altitudes, crop age, ~~*Eldana saccharina*~~, incidence, seasons, sugarcane varieties, agroecological zones

1.0 Introduction

Sugarcane is a perennial, high-value commercial crop cultivated globally to meet increasing sugar demand (1,2). Current production exceeds two billion metric tons annually, with Brazil leading global output at approximately 798 million metric tons, followed by India at 421.15 million metric tons (3). In Sab-Saharan Africa, South Africa is the dominant producer, contributing about 17.94 million metric tons, while in East Africa, Uganda leads regional production with approximately 6.19 million metric tons (2). Despite its economic significance, sugarcane productivity is constrained by multiple biotic and abiotic factors (4,5). Abiotic factors such as drought, extreme temperature and soil nutrient limitations affect sugarcane growth and sucrose accumulation (6). Biotic factors, particularly insect pests, pose substantial threats to sugarcane yields. Among these, stem borers are recognized as the most destructive pests in sugarcane production. Key species of global importance include *Eldana saccharina* in Africa, *Diatraea saccharalis* in the Americas, *Chilo sacchariphagus* in Asia and the Indian Ocean region, and *Scirpophaga exerptalis* in South Asia (7,8). In Sub-Saharan Africa, *E. saccharina* is regarded as the most economically important

stem borer because it causes substantial losses in cane yield and sucrose content (9). Like other stem borers, *E. saccharina* is also difficult to manage effectively, as its larvae develop internally within sugarcane stalk tissues, where they are protected from contact insecticides and less accessible to natural enemies, thus, reducing the effectiveness of conversional control measures (10).

The pest was first recorded in West Africa more than a century ago and subsequently spread across major sugarcane-producing regions including Mozambique, South Africa, Zimbabwe, Benin, Kenya, Uganda and Tanzania (11). In South Africa, *E. saccharina* became established in the early 1900s and was formally recognized as a serious economic pest by 1939 after documented yield reductions up to 30% and sucrose losses approaching 18% (12). Similar economic importance was later reported in East Africa, where the pest was recorded in Tanzania in the early twentieth century before spreading regionally (13).

The distribution of *E. saccharina* has not remained static. Historically, confined largely to coastal and lowland regions in countries like South Africa and Ethiopia (14–16), the pest has progressively expanded into inland and higher altitude sugarcane-producing zones (17). Such shifts have been associated with environmental and climatic changes (18,19). Seasonal rainfall patterns, altitude-related temperatures, crop age and sugarcane variety susceptibility have been reported to influence infestation level, larval survivability, and the extent of internal stalk damage (12,20–22). For example, increased infestation levels have been documented during dry seasons in lowland areas of South Africa and Ethiopia (14,15,17), whereas in some coastal high-altitude environments, peak infestation occurs during the rainy season (12). Older sugarcane (> 12 months) is frequently associated with higher larval densities and greater internal tissue redness, partly due to favorable microclimatic conditions and increased oviposition sites (1,23,24) . In addition, cultivation of susceptible varieties, including N46 and N49, has been linked to increased infestation intensity and concentrated damage in bottom stalk part (9).

Despite extensive regional documentation, information on the status and spatial distribution of *E. saccharina* in Tanzania remained obscured. The existed information were mainly speculations on the

history of occurrence and a few unpublished reports that were insufficiently quantitative with limited evidence on infestation incidence, intensity, and within-stalk-damage patterns across Tanzanian agroecological zones. Given the environmental variability and recent expansion on sugarcane cultivation into new production areas, an updated assessment of the pest's status was required. Therefore, this study aimed at establishing the status of *E. saccharina* in Tanzania by quantifying infestation across high, medium, and low altitude agroecological zones. Specifically, the study assessed larval density (number of *E. saccharina* per 50 stalks), percentage of stalks bored, percentage of internal stalk tissue damage, and distribution of bored plant parts to determine spatial variation in infestation intensity and damage severity (9,20,25). The study also explored the sugarcane varieties that contributed to the increased *E. saccharina* infestations across the altitudes. By providing a cross-sectional evaluation across environmental gradients, the findings contribute fundamental information necessary for deployment of strategic zone-specific management of the pest in Tanzania sugarcane production systems.

2.0 Materials and methods

2.1 Description of the study area

The study was conducted across three agroecological zones categorized by altitude: high, medium, and low (3). The low-altitude area included sugarcane fields situated at altitudes below 600 meters above sea level (m a.s.l), while fields at elevations between 601 and 1000 m a.s.l were classified as medium altitude. The high-altitude zone comprised sugarcane fields located above 1000 m a.s.l (26). Each altitude zone represented sugarcane fields belonging to different sugarcane producing companies. The companies included Kagera Sugar Company Limited, located at high altitude between S 1° 00' to 2° 45', and E 30° 25' to 32° 40' at altitude of 1400 m a.s.l in Kagera Region northwestern part of Tanzania; Tanganyika Planting Company Limited, located at medium altitude S 3° 45' and E 37° 37' at an altitude of 950 m a.s.l in Kilimanjaro Region in northeastern Tanzania; and two companies, Kilombero Sugar Company Limited

and Mtibwa Sugar Estates at low-altitude zone, located at S 7º 44’, E 37º 00’ and S 6º 09’, E 37º 50’ respectively, in Morogoro Region in eastern Tanzania.

2.2 Experimental design

A stratified, multistage cross-sectional sampling design was implemented in the survey undertaken to assess and quantify *E. saccharina* infestation across altitudes. Altitude (high, medium, and low) constituted the primary strata, within each stratum, sugarcane fields in a company serving as the sampling frame. Sugarcane block disposition maps were obtained from each company and used to generate a complete list of cultivated units. This was followed with selection of blocks using a finite population sampling approach. The required number of blocks per stratum was estimated using finite population formula by Mcmaugh, (2005) as follows:

$n = \frac{N}{(1+N(e)^2}$ (1) . Whereby n is the required sample size, N is the total number of cultivated blocks in respective sugarcane-producing company, and “e” is the allowable margin of error (set at 5%).

From the total numbers of cultivated blocks which was 2933 blocks computed from 2022 units in the high altitude, 317 units in the medium altitude, and 594 units in the low altitude, the established sample sizes were; 325 blocks in the high altitude, 175 blocks in the medium altitude, and 211 blocks in the low altitude zones.

Field surveys were conducted during both rainy and dry seasons in 2025 to capture seasonal variation in *E. saccharina* infestation. Assessments were performed in February-March (rainy season) and May-June (dry season) at high altitude zone, March-April (rainy season) and July-August (dry season) at the medium altitude and March (rainy season) and August-September (dry season) at the low altitude.

2.3 Sampling and sampling design

Random sampling was made from the complete list of identified blocks to ensure spatial coverage across the entire estate. Sugarcane crop aged ≥ 3 months were selected for assessment targeting the stalks that have developed sufficient internodes and tissue structure to allow *E. saccharina* larval boring and establishment for detection of infestation and internal damage as per other workers(9,28). Each randomly selected block constituted a primary sampling unit. Assessment of *E. saccharina* infestation within blocks followed the standardized field survey protocol developed by SASRI (2023). Within each selected block, sampling was conducted along diagonal transects to ensure representative spatial coverage. A total of 50 sugarcane stalks were randomly selected per block, distributed evenly along the transects extending from one corner of the block to the opposite corner. To capture both edge and central field conditions, 40 stalks were sampled along the block peripheral (edges) at regular intervals, while an additional 10 stalks were sampled along the central transect. At each sampling point, stalks were selected at random and assessed for infestation and damage parameters following the established protocol. This systematic spatial alaltitude reduced edge or clustering effects and ensured coverage of both peripheral and interior block sections.

Sampling was conducted at the block level, with block sizes ranging from 5 to 22 ha. Although the SASRI protocol recommends sampling up to 100 stalks in blocks of ≤ 5ha, estate management restricted destructive sampling to a maximum of 50 stalks per block. Consequently, a fixed sample size of 50 stalks was applied uniformly across all blocks, regardless of block size. This approach ensured methodological consistency and comparability of infestation estimates across blocks and altitudes, as each block constituted the primary management unit in the estates and was therefore treated as the independent sampling unit for infestation assessment.

2.4 Data collection

Within each sampled block, 50 sugarcane stalks were randomly selected and assessed individually, each stalk was cut approximately 5 cm above ground level and initially examined for visible signs of *E. saccharina* infestation, including characteristic entry or exit holes. The position of damage along the stalk was recorded as bottom, middle, or top. Each stalk was then split longitudinally into two halves (half of four parts) to allow internal inspection. The total number of internodes per stalk and the number of bored internodes were recorded. The presence and number of *E. saccharina* larvae and/or pupae were documented. Internal tissue damage was quantified by measuring the length of red discoloration within the stalk (stalk length red (m)), which served as proxy for pest-induced internal tissue. The recorded data therefore included block name, sugarcane variety, collection date, crop age (months), stalk length (m), number of internodes per stalk, number of internodes bored, stalk length red (m), position of damage along the stalk, and number of *E. saccharina* larvae and/or pupae detected.

2.5 Data analysis

Raw field data were first summarized into infestation and damage metrics following standard procedures described by the South African Sugarcane Research Institute SASRI (2023). Infestation intensity was expressed as the number of *Eldana saccharina* larvae and/or pupae per 50 stalks (e/50 stalks). Boring intensity was calculated as the proportion of internodes bored per stalk, while internal tissue damage was quantified as the proportion of the stalk length exhibiting red discoloration (total stalk length red/total stalk length). In addition, the presence of boring along the stalk was categorized by position (bottom, middle, top) for subsequent analysis.

Prior to modelling, the distribution of the response variables was examined using graphical and diagnostic methods to guide model selection. All responses were analysed within a generalized linear mixed modelling (GLMM) framework to account for abnormal distribution of data and the hierarchical sampling

design. Count data were modelled using a negative binomial error distribution with a log link function, as this distribution accommodates the discrete, non-normal and overdispersion characteristics of count responses. Proportion data (bounded between 0 and 1) were analysed using GLMMs with a beta error distribution and logit link function.

Specifically, infestation intensity (e/50 stalks) was analysed using GLMMs with a negative binomial distribution and log link function in R using glmmTMB package (29). Season (rainy and dry), altitude (high, medium and low), and crop age (centred continuous variable) were included as fixed effects with their interactions, while Block names were included as random factor to account for the hierarchical sampling structure and non-independence of observations within blocks. The fitted model was:

$$\log(\mu) = \beta_0 + \beta_1 \text{season} + \beta_2 \text{altitude} + \beta_3 \text{crop age} + \text{ interactions} + b_{block} \quad (2)$$

Where (μ) is the expected number of *E. saccharina individuals* per 50 stalks, β_0 is the intercept, β terms represent fixed effects and their interactions, and b_{block} is the random effect associated with block.

Proportion-based response variables (internal tissue damage and boring intensity) were analyzed using GLMMs with a beta error distribution and logit link function. Seasons, altitude and crop age (centered continuous) were included as fixed effects, together with their interactions to evaluate whether damage severity varied across altitudes and developmental stages while block name was fitted as a random factor. The fitted models were:

i. Beta model for percentage stalk length red

$$\text{logit}(\pi) = \text{season x altitude x poly}(\text{crop age}_c, 2) + \mathrm{b_{block}} \quad (3)$$

Where π is the expected proportion of internodes bored and b_{block} is the random effect of the block. The polynomial term for crop age was included to accommodate potential non-linear age effects.

ii. Binomial model for percentage stalk bored

$$\text{logit}(\theta) = \text{season x altitude x crop age}_c + b_{block} \quad (4)$$

Where θ is expected proportion of the internodes bored and b_{block} is the random block effect

Boring incidence along sugarcane stalk parts (bottom, middle, and top) was also analysed using a generalized linear mixed model (GLMM) with a binomial error distribution and logit function, as the response variable was binary (presence/absence of boring). Stalk part, season, altitude, and their interactions were included as fixed effects to evaluate dependent variation in infestation along the sugarcane stalk. Block name was fitted as a random factor to account for non-independence of observations within sampling units. The variation in boring incidence along sugarcane stalk parts was modelled as:

$$\text{Logit}_{pijkl} = \beta_0 + \beta_1\text{part}_i + \beta_2\text{season}_j + \beta_3\text{altitude}_k + \beta_4\text{crop age}_l + \text{interactions} + b_{block} \quad (5)$$

Where $pijkl$ is the probability of boring occurrence, β_0 is the intercept, β terms represent fixed effects and their interactions, and b_{block} is the random effect associated with block.

model adequacy was assessed using simulation-based residual diagnostics implemented in the *DHARMa* package (30), including tests for dispersion, zero inflation, and deviations from model assumptions, supported by visual inspection of residual patterns The statistical significance of fixed effects and their interactions for infestation intensity and proportional based responses was assessed using likelihood ratio tests. For the binomial model of boring incidence along stalk parts, statistical significance was assessed using Type III Wald x^2 tests implemented in the *car package* (31). Where significant effects were detected, post hoc pairwise comparisons of estimated marginal means were performed using the *emmeans* package (32), with Tukey-adjusted p-values. Estimated marginal means were presented with 95% confidence intervals ($p \leq 0.05$).

3.0 Results

3.1 Variations in *E. saccharina* infestation across altitudes

The Likelihood Ratio Test (LRT) at 95% confidence level (Table 1) revealed that season, altitude and crop age significantly influenced the varied *E. saccharina* infestation across the three altitudes($P < 0.05$). A significant two-way interaction was also observed between season and crop age ($P < 0.001$). Further to this, a three-way interaction between season, altitude, and crop age was highly significant($x^2 = 9.58$, $P < 0.0083$). However, when crop age was not considered, LRT indicated that seasonal differences in infestation did not differ consistently across altitudes ($x^2 = 0.39, P = 0.82$). The post-hoc analyses indicated that the significant season, altitude, and crop age interaction was primarily driven by a strong positive effect of crop age on *E. saccharina* infestation at high altitude during the rainy season, whereas crop-age effects were non-significant at low and medium altitudes and during the dry season (Fig 1).

Fig 1: Post-hoc estimates of crop-age effect (centered crop age. Asterisks (***) denotes a significant difference at p<0.001, ns = not significant

Table 1: Likelihood Ratio Test (LRT) evaluating the effect of season, altitude, crop age, and their interactions on *E. saccharina* infestations in Tanzania.

Source of variation	Chi-square (x^2)	P-value
Season	56.44	<0.001
Altitude	341.42	<0.001
Crop age	43.91	<0.001
Season*altitude	0.39	0.82
Season*crop age	23.57	<0.001
Altitude*crop age	12.81	0.017
Season*altitude*crop age	9.58	0.008

The *E. saccharina* infestation generally increased with crop age (Fig. 2). However, such observation was only statistically significant at high altitude, particularly beyond 12 months of crop age, and was most pronounced during the rainy season.

Fig 2: *E. saccharina* infestation across crop age by season and altitude. Shaded bands represent 95% confidence intervals

The post-hoc analyses further revealed that there was a significant three-way interaction of season, altitude and crop age. However, during the dry season, significant seasonal differences ($P < 0.05$) were detected primarily in sugarcane crops with ≤ 12 months crop age, whereas in older crops (> 12 months), significant seasonal variation was evident ($P < 0.01$) only during the rainy season. At low altitude, infestation levels remained close to zero across all crop ages in both seasons. At medium altitude, a significant seasonal difference was detected only at early crop age approximately 6 months, while no significant seasonal differences were observed at later crop ages, indicating weak seasonal effect (Fig. 3).

Fig 3: Seasonal effects on *E. saccharina* infestation across altitudes and crop ages; † $P > 0.1$, * $P < 0.05$, ** $P < 0.01$, *** $P < 0.001$, ns = not significant

3.2 Extent of internal red stalk tissue damage caused by *E. saccharina* infestation

The likelihood ratio test (Table 2) showed that damage intensity expressed as the percentage stalk length red was highly significantly influenced by season, altitude and crop age rather than being driven by any single factor (all $\mathbf{P < 0.001}$).

Table 2: Likelihood ratio test results for effects of season, altitude, and crop age, and their interactions on percentage stalk length red caused by *E. saccharina*

Source of variation	Chi-square (x^2)	P - value
Season	480.1	< 0.001
Altitude	218.1	< 0.001
Crop age	109.1	< 0.001
Season*altitude	89.71	< 0.001
Season*crop age	14.92	< 0.002
Altitude*crop age	34.77	< 0.001
Season*altitude*crop age	11.52	<0.003

The post-hoc analyses (Table 3) showed that seasonal effects on internal tissue damage varied across altitudes. At low altitude, internal stalk damage was significantly higher during the dry season ($\mathbf{P < 0.0072}$). In contrast, at high altitude, the negative seasonal estimate indicated significantly higher internal tissue damage during the rainy season ($\mathbf{P < 0.04}$). At medium altitude, seasonal differences in the internal stalk tissue damage were not statistically significant.

Table 3: Seasonal effect on damages of internal sugarcane stalk tissues caused by *E. saccharina*

Altitude	Contrasts	Estimate	SE	Z-ratio	P – value
Low altitude	Dry-Rain	0.07	0.03	2.69	0.0072
Medium altitude	Dry-Rain	-0.15	0.21	-0.71	0.48
High altitude	Dry-Rain	-0.06	0.03	-1.97	0.04

With respect to crop age, post-hoc analyses further indicated that the percentage of stalk length red increased with increasing crop age (Fig. 4). At low altitude, internal stalk tissue damage remained predominantly within the average class across crop ages. At high altitude, the damage increased markedly

with crop age, reaching significantly high levels from approximately 12 months onward in both dry and rainy seasons. In contrast, internal stalk damage tended to increase with crop age at medium altitude and significantly high infestation ($P < 0.01$) was observed during the dry season (Fig. 4).

Fig 4: Damages of internal sugarcane stalk tissues caused by *E. saccharina* across crop ages in each season and altitude

3.3 Spatiotemporal variation in sugarcane stalk boring intensity

The percentage sugarcane stalk bored by *E. saccharina* infestation varied significantly with season, altitude, and crop age (Table 4). Likelihood ratio tests revealed statistically significant main effects of season, altitude and crop age, as well as significant two-way and three-way interactions among these factors ($P < 0.003$)**.** These results indicate that stalk-boring intensity is structured by the combined influence of seasonal conditions, altitudinal differences, and developmental stages, rather than by a single factor acting independently.

Table 4: Likelihood ratio test for the percentage sugarcane stalk bored caused by *E. saccharina*

**Source of variation**	**Chi-square (x^2)**	**P – value**
Season	480.05	< 0.001
Altitude	218.71	< 0.001
Crop age	109.13	< 0.001
Season*altitude	89.71	< 0.001
Season*crop age	14.92	0.002
Altitude*crop age	34.77	< 0.001
Season*altitude*crop age	11.51	0.003

Crop age was revealed by the post-hoc analysis to have positive effect on the percentage of sugarcane stalks bored across altitudes, as stipulated by significantly positive crop-age slopes in both seasons (Table 5). The negative estimate values in the medium and high altitudes showed that the percentage boring was significant ($P < 0.013$, and 0.004), during the rainy season.

Table 5: Seasonal effect on the percentage sugarcane stalks bored across altitudes and crop ages

**Altitude**	**Contrasts**	**Estimate**	**SE**	**Z-ratio**	**P – value**
Low altitude	Dry-Rain	0.02	0.02	0.95	0.34
Medium altitude	Dry-Rain	-0.10	0.04	-2.48	0.013
High altitude	Dry-Rain	-0.05	0.02	-2.83	0.004

The boring percentage was further observed to be high in both high and medium altitudes, specifically to crop ages with approximately 12 months and above (Fig. 5). On the other hand, low to average boring percentages were noted in low altitude

Figure 5: Influence of crop age on the percentage sugarcane stalk bored caused by *E. saccharina* across the seasons and altitudes

3.4 Determinants of sugarcane stalk part boring by *E. saccharina*

Type III Wald test revealed that the probability of *E. saccharina* boring a sugarcane stalk was strongly influenced by stalk part, season, altitude, and crop age, as indicated by highly significant main effects and the interactions (Fig. 6). This confirms that infestation is not uniform within the plant across seasons and altitudes.

Figure 6: Effects of season, altitude, crop age and their interaction in different bored parts of sugarcane stalks due to *E. saccharina* infestation

Predicted boring probabilities of *E. saccharina* differed among sugarcane stalk parts depending on season and altitude (Fig. 7). In all season and altitude combinations, the bottom stalk part exhibited the highest predicted probability of boredom, whereas the top part consistently showed the lowest values, with the middle part intermediate. Post-hoc analysis revealed significant differences among stalk parts at medium and high altitudes in both dry and rainy seasons. At low altitude, part-specific differences were less pronounced during the rainy season, where the middle and top stalk parts did not differ significantly, although both remained less bored than the bottom part. Generally, infestation followed a clear vertical gradient along the stalk, modulated by the environmental conditions (Fig. 7)

Figure 7: Model-predicted probabilities of *E. saccharina* boring among sugarcane stalk parts across seasons and altitudes, with bars showing estimated means ± 95% confidence intervals and brackets with asterisks indicating significant pairwise differences within each season and altitude combination

3.5 Sugarcane varietal influence on *E. saccharina* infestation within altitudes

In the high altitude, infestation intensity was markedly greater than in the low altitude (Fig.8). Within the high-altitude, sugarcane fields with mixed varieties and N49 exhibited the highest predicted infestation levels, although several varieties did not differ statistically from each other. In contrast, infestation at low altitude remained uniformly low, with no significant varietal differences, despite slightly higher mean counts in N36 and N25. Similarly, varietal differences at medium altitude were not statistically significant, although mixed varieties and N30 showed comparatively higher numerical infestation levels.

Figure 8: *E. saccharina* infestation intensity across altitudes and sugarcane varieties. Bars sharing the same letter suggest a statistical insignificance in infestation intensity at $P \leq 0.05$

4.0 Discussion

The presented findings suggest that *E. saccharina* infestation and resultant damages were influenced by significant interactions among season, altitude, and crop age. Infestation increased with crop age at high altitude during both rainy and dry seasons across crop ages. More counts of *E. saccharina* were observed in older crops (>12 months), especially during the rainy season. Despite the generally low infestations at low altitude, the recorded internal stalk tissue damage was relatively higher particularly during the dry season. At high altitude the internal damage was high in both dry and rainy seasons, especially in older crops. Medium-altitude sites, however, was manifested with significantly elevated infestation during the dry season, especially in crops older than 11 months. Elevated boring intensity was recorded at medium and high altitudes with the bottom parts of the stalk being mostly affected. Fields with mixed varieties (particularly N41 and N49) exhibited higher infestation with *E. saccharina* at medium and high altitudes, while N36 and N25 sugarcane varieties had higher mean counts of the borer at low altitude.

Similar observations have been reported in northern Ivory Coast (20,33) that *E. saccharina* infestation increased during the rainy season in sugarcane fields. Whereas their study was primarily structured around establishing the effect of cropping seasons, the present study further incorporates altitudinal gradients and crop age to increase clarity on the interacting environmental drivers of infestation intensity. Similarly, Mulcahy et al. (2023), in evaluating push-pull technology for *E. saccharina* management in South Africa, documented higher incidences of the pest in highland areas compared to mid-altitude and coastal regions, underscoring the role of altitudinal variations in shaping infestation risks. The elevated infestations observed at high altitude during the rainy season in this study may be attributed to prolonged crop growth particularly in fields exceeding 12 months, which led to thicker stalks and greater biomass favoring suitable environment for the pest survival. Such structural characteristics increase oviposition opportunities and provide favorable larval refugia, thereby enhancing establishment and survival of the pest (22).

Studies elsewhere suggested that water stress increases susceptibility of sugarcane to *E. saccharina*, resulting in greater larva penetration and elevated boring intensity that increase the internal stalk tissue damage (9,35). A comparable pattern was observed in the present study, whereby significantly higher internal tissue damage occurred at low altitude during the dry season, when moisture limitation was most pronounced. Under such conditions, physiological stress likely reduces stalk resistance and facilitates larvae infestation and establishment (20). Consistent with stem borer ecology, neonates rapidly penetrate plant tissue, where the internal environment provides a buffered microclimate that enhances survival relative to exposed conditions (13,36). However, the greater boring intensity and internal tissue damage recorded at high and medium altitudes during the rainy season likely reflect a different pattern and mechanisms that triggers boring of canes. In these environments, increased moisture and favorable thermal conditions could have enhanced *E. saccharina* survival, development, and population growth (21,25), thereby increasing infestation pressure.

Adult *E. saccharina* preferentially oviposit beneath older leaves of sugarcane which are predominantly located at the bottom part of the stalk (9,25). Because eggs deposition occurs near these bottom nodes, newly hatched larvae typically penetrate the nearest accessible internodes, resulting in a greater concentration of boring in the bottom part compared to middle and top parts (37). The vertical distribution of leaf sheaths therefore directly influences the spatial pattern of larval establishment (14). In the present study, sugarcane crops older than 12 months exhibited significantly higher boring intensity in the bottom third part of the stalk, consistently matching the reported pattern that *E. saccharina* is a pest of mature sugarcane (38). Available reports further suggest that females *E. saccharina* prefer to oviposit on dead leaves which concentrate around the lower stalk parts (6,38). Thus, the significant damage to the bottom part of the stalk may reflect on the greater suitability of older cane for *E. saccharina* infestation.

The varieties N41 and N49 had the highest *E. saccharina* infestation, particularly at high altitude. These cultivars have previously been classified as susceptible under South African production conditions (9),

suggesting that varietal characteristics could play the predisposition factor to the elevated infestation as observed in fields at high altitude. The comparatively greater pest pressure was recorded in fields with mixtures of varieties with inclusion of susceptible varieties within the resistant/tolerant ones (20). Whenever highly susceptible varieties are incorporated in resistant ones, the former tends to act as focal points for *E. saccharina* oviposition with subsequent larval build-up, thereby increasing overall infestation intensity and associated internal tissue damage.

The obtained results have great implications for sugarcane-producers as the crop age has been reported to have a significant influence on increasing *E. saccharina* infestations across the seasons and altitude. Thus, early harvesting should be considered when developing an Integrated Pest Management (IPM) package against the pest, specifically in the sugarcane fields located at the high and medium altitudes where the *E. saccharina* infestation and boring intensity are highly pronounced. As the method has been observed to reduce the yield losses associated with *E. saccharina* infestation (33,39).

Although the present study provides important insights into the infestation patterns of *E. saccharina* in Tanzania, its scope was limited to sugarcane-producing companies located across the selected altitude gradient translated to different altitude. These companies typically grow improved varieties reported to possess intermediate to substantial resistance to *E. saccharina* (9,39,40), and the farming is characterized by year-round irrigation service which supplements the cane abilities to resist the pest infestation due to improved crop vigor and limited water stress which buffers the pest damage (41). Thus, a different scenario is expected in smallholder production systems where access to irrigation and fertilization are probably limiting. Because such provisions are not commonly practiced, findings from the current study should be interpreted with caution when considering the management options for *E. saccharina* under small holder farming systems. When extrapolating these results to the broader national context, such dynamics should be taken on-board to avoid misguiding the sugarcane growers. Based on the limitations of the current study that the pest infestation trend and dynamics under small holing farming systems was

not covered, we recommend that future studies should endeavor to include farmers' fields to enable a more representative and reliable conclusion on the infestation status of *E. saccharina* in Tanzania.

5.0 Conclusion

Eldana saccharina infestation has been demonstrated to be strongly structured by season, altitude, and crop age. The high-altitude agroecological zone consistently recorded the highest *E. saccharina* infestation in both seasons. Elevated infestation levels were observed during the dry season across younger than or approximately equal to 9 months old stalk, whereas in the rainy season infestation was predominantly concentrated in older crops (>12 months), showing elevated boring intensity and internal stalk tissue damage. Although infestation levels were lower at the low-altitude, internal tissue damage was comparatively high, indicating that visible pest density does not always reflect physiological injury. Damage was concentrated in the bottom stalk part, while the top sections were least affected. Mixed-variety planting, especially involving N41 and N49, increased susceptibility at high altitude. These findings suggest that management practices against *E. saccharina* should be tailored to specific agroecological conditions and crop growth stages rather than applying uniform interventions across all production areas.

Acknowledgement

The management of sugarcane-producing companies in Tanzania namely; Kagera Sugar Company Limited, Tanganyika Planting Company Limited (TPC), Kilombero Sugar Company Limited, and Mtibwa Sugar Estates are acknowledged for granting the permission to use their sugarcane fields for this study and their experienced technical personnel to support the data collection phase.

6.0 References

1. Singels A, Jackson P, Inman-Bamber G. Sugarcane. Crop Physiology Case Histories for Major Crops. 2020; 674–713. doi:10.1016/B978-0-12-819194-1.00021-9

2. Food and Agriculture Organization. Agricultural production statistics 2010–2023. Rome, Italy; 2024.

3. Food and Agriculture Organization of the United Nations. Global Agro-Ecological Zones (GAEZ v4) – Model Documentation. Rome, Italy and Laxenburg, Austria; 2021.

4. Schultz N, Pereira W, de Albuquerque Silva P, Baldani JI, Boddey RM, Alves BJR, et al. Yield of sugarcane varieties and their sugar quality grown in different soil types and inoculated with a diazotrophic bacteria consortium. Plant Prod Sci. 2017; 20(4):366–74. doi:10.1080/1343943X.2017.1374869

5. Mehdi F, Cao Z, Zhang S, Gan Y, Cai W, Peng L, et al. Factors affecting the production of sugarcane yield and sucrose accumulation: suggested potential biological solutions. Front Plant Sci. 2024; 15:1374228. doi:10.3389/fpls.2024.1374228

6. Li AM, Chen ZL, Liao F, Zhao Y, Qin CX, Wang M, et al. Sugarcane borers: species, distribution, damage and management options. Journal of Pest Science. 2024; 97(3):1171–201. doi:10.1007/s10340-024-01750-9

7. Wilson BE, Salgado LD, Villegas JM. Optimizing chemical control for *Diatraea saccharalis* (Lepidoptera: Crambidae) in sugarcane. Crop Protection. 2022; 152: 105843. doi: 10.1016/j.cropro.2021.105843

8. He-Ling L, Ji-Li W, Yong-Hao Y, Xian-Ru Z, Ren-Fu M, Yuan-Yuan G, et al. Rhythms of pheromone titer in 2 sugarcane pest species *Chilo sacchariphagus* and *Chilo infuscatellus*

(Lepidoptera: Crambidae). Journal of Insect Science. 2025 Oct 1;25(5): ieaf084. doi:10.1093/jisesa/ieaf084 PubMed PMID: 41060987.

9. South African Sugarcane Research Institute (SASRI). Controlling Eldana in the South African Sugar Industry. Mount Edgecombe, South Africa; 2023.

10. Msele KP, Munyai C, Albertse EH, Malinga LN. Baseline Susceptibility of *Eldana saccharina* to Coragen® SC: Implications for Resistance Monitoring and Management in Sugarcane. Biology and Life Sciences Forum 2025, Vol 54, Page 8. 2026 Jan 16;54(1):8. doi:10.3390/blsf2025054008

11. Peter B, Anne W, Silke S. Express-PRA for *Eldana saccharina*. Indonesia; 2020 Oct.

12. Mélanie B, Yah CO, Goebel FR. Outbreaks of the African sugarcane stalk borer *Eldana saccharina* Walker (Lepidoptera: Pyralidae) in sugarcane plantations of the northern Ivory Coast: management strategies under implementation. Proceedings of the International Society of Sugar Cane Technologists. 2019; 30:880–9.

13. Kfir R, Overholt WA, Khan ZR, Polaszek A. Biology and management of economically important lepidopteran cereal stem borers in Africa. Annu Rev Entomol. 2002 Jan 1;47(Volume 47, 2002):701–31. doi: 10.1146/annurev.ento.47.091201.145254 PubMed PMID: 11729089.

14. Assefa Y, Conlong DE, van den Berg J, Mitchell A. Distribution of sugarcane stem borers and their natural enemies in small-scale farmers' fields, adjacent margins and wetlands of Ethiopia. Int J Pest Manag. 2010 Jul;56(3):233–41. doi:10.1080/09670870903470223

15. Assefa Y, Conlong DE, Mitchell A. Status of *Eldana saccharina* (Lepidoptera: Pyralidae), its host plants and natural enemies in Ethiopia. Bull Entomol Res. 2006 Oct;96(5):497–504. doi:10.1079/ber2006451 PubMed PMID: 17092361.

16. Goebel FR, Way MJ, Gossard C. Proceedings of the South African Sugar Technologists' Association. In: The status of *Eldana saccharina* (Lepidoptera: Pyralidae) in the South African sugar industry based on regular survey data. Mount Edgecombe, South Africa: South African Sugar Technologists' Association (SASTA); 2005. p. 337–46.

17. Assefa Y, Van Den Berg J, Mitchell A, Le Rü BP, Conlong DE. Record of *Eldana saccharina* Walker (Lep, Pyralidae) in inland South Africa and its genetic relationship with the coastal population. Journal of Applied Entomology. 2009 Jul;133(6):449–55. doi:10.1111/j.1439-0418.2009. 01390.x

18. King H, Conlong DE, Mitchell A. Genetic differentiation in *Eldana saccharina* (Lepidoptera: Pyralidae): Evidence from the mitochondrial cytochrome oxidase I and II genes. Proc S Afr Sug Technol Ass. 2002.

19. Peng Y, Jin M, Li Z, Li H, Zhang L, Yu S, et al. Population genomics provide insights into the evolution and adaptation of the Asia corn Borer. Mol Biol Evol. 2023; 40(5). doi:10.1093/molbev/msad112 PubMed PMID: 37155936.

20. Msomba BH, Ndaki PM, Joseph CO. Pest dynamics in relation to climate and varietal resistance: implications for sustainable management in Kagera sugar plantation, Tanzania. Discover Agriculture. 2025; 3(1). doi:10.1007/s44279-025-00336-y

21. Kleynhans E, Conlong DE, Terblanche JS. Host plant-related variation in thermal tolerance of *Eldana saccharina*. Entomol Exp Appl. 2014; 150(2):113–22. doi:10.1111/eea.12144

22. Ntirenganya E, Goftishu M, Sokame BM, Assefa Y, Nsengimana V. Pest status and dynamic of sugarcane stemborer (*Eldana saccharina*) in sugarcane plantations of Nyabugogo tropical Wetland, Rwanda. Int J Trop Insect Sci. 2025. doi:10.1007/s42690-025-01690-6

23. Crépin BP, Mélanie BB, François RG, Yah CO. Early Harvesting of Rainfed Plant Cane to Prevent Heavy Infestations of *Eldana saccharina* (Lepidoptera, Pyralidae) in Ferké Sugarcane plantations, Ivory Coast. JASAE. 2019; 15(3):1–9.

24. Ramburan S. Interactions affecting the optimal harvest age of sugarcane in rainfed regions of South Africa. Field Crops Res. 2015; 183:276–81. doi: 10.1016/j.fcr.2015.08.003

25. Kleynhans E, Barton MG, Conlong DE, Terblanche JS. Population dynamics of *Eldana saccharina* Walker (Lepidoptera: Pyralidae): Application of a biophysical model to understand phenological variation in an agricultural pest. Bulletin of entomological research. Cambridge University Press; 2018: 283–94. doi:10.1017/S0007485317000712 PubMed PMID: 28786374.

26. United Republic of Tanzania. Mwongozo wa uzalishaji mazao kulingana na kanda za kiikolojia. Dodoma; 2022.

27. Mcmaugh T. Guidelines for surveillance for plant pests in Asia and the Pacific. 2005 [cited 2026 Feb 14]. Available from: https://ageconsearch.umn.edu/record/114089/files/119.pdf

28. Brooks M, Bolker B, Kristensen K, Maechler M, Magnusson A, Skaug H, et al. Generalized Linear Mixed Models using template model builder [R package glmmTMB version 1.1.14]. CRAN: Contributed Packages. 2026. doi: 10.32614/CRAN.package.glmmTMB

29. Hartig F. DHARMa: Residual diagnostics for hierarchical (multi-level/mixed) regression models. R package (CRAN); 2024.

30. Fox J, Weisberg S, Price B. Companion to applied regression [R package car version 3.1-5]. CRAN: contributed packages. 2026 Feb 3. doi: 10.32614/CRAN.package.car

31. Russell V. Lenth, Julia Piaskowski. emmeans: Estimated marginal means, aka least-squares means. R package (CRAN); 2025.

32. Mulcahy MM, Conlong DE, Hill MP. Determining the efficacy of push-pull for management of *Eldana saccharina* (Walker (Lepidoptera: Pyralidae) in sugarcane through on-farm field trials. African Entomology. 2023;31(1). doi:10.17159/2254-8854/2023/a15654

33. Kvedaras OL, Keeping MG, Goebel FR, Byrne MJ. Larval performance of the pyralid borer *Eldana saccharina* Walker and stalk damage in sugarcane: Influence of plant silicon, cultivar and feeding site. International journal of pest management. 2007: 183–94. doi:10.1080/09670870601110956

34. Kfir R, Overholt WA, Khan ZR, Polaszek A. Biology and management of economically important Lepidopteran cereal stem borers in Africa. Annu Rev Entomol; 2002(47): 701–31. doi: 10.1146/annurev.ento.47.091201.145254 PubMed PMID: 11729089.

35. Rutherford RS. IPM for Eldana control: An Integrated Pest Management (IPM) approach for the control of the stalk borer *Eldana saccharina* Walker (Lepidoptera: Pyralidae). 2015 [cited 2026 Mar 13]; Available from: http://www.sasa.org.za/Files/BM_IPM for eldana.pdf

36. Péné CB, Boua MB, Coulibaly-Ouattara Y, Goebel FR. Stem Borer (*Eldana saccharina* W) Infestation outbreak in sugarcane plantations of Northern Ivory Coast: Management strategies under implementation. American Journal of Bioscience and Bioengineering. 2019; 6(4):20–28. doi: 10.11648/j.bio.20180604.11

37. Horton PM, Hearne JW, Apaloo J, Conlong DE, Way MJ, Uys P. Investigating strategies for minimising damage caused by the sugarcane pest *Eldana saccharina*. Agric Syst. 2002; 74(2): 271–86. doi:10.1016/S0308-521X (01)00089-0

38. Bhatt R, Kumar R, Kashyap L, Alataway A, Dewidar AZ, Mattar MA. Growth, yield, quality and insect-pests in sugarcane (*Saccharum officinarum*) as affected by differential regimes of irrigation and potash under stressed conditions. Agronomy. 2022; 12(8):1942. doi:10.3390/agronomy12081942

Supporting information

S 1 Table. Raw data used for the analysis process

 Click here to access/download;Figure;Fig 1.tif

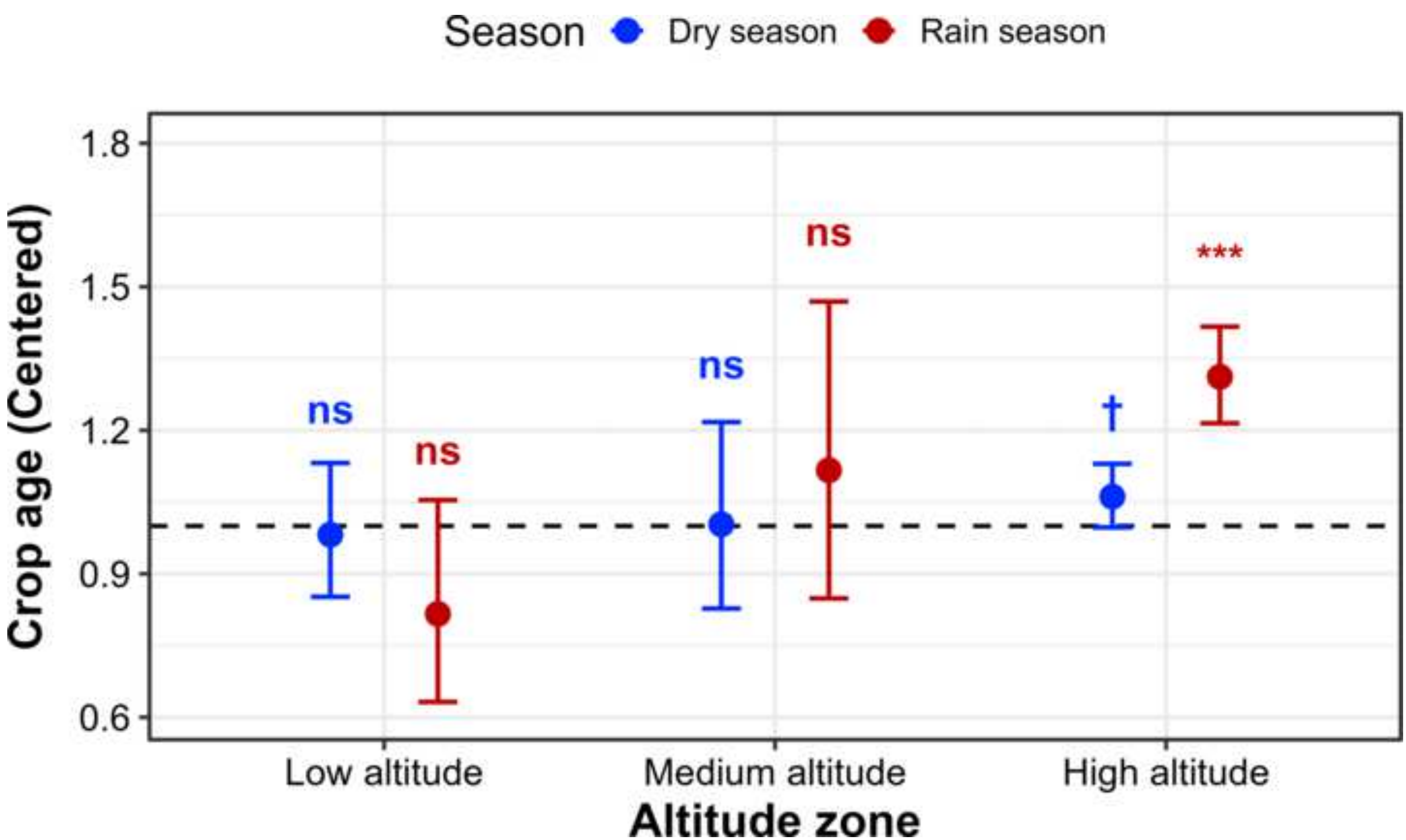

Figure

Click here to access/download;Figure;Fig 2.tif

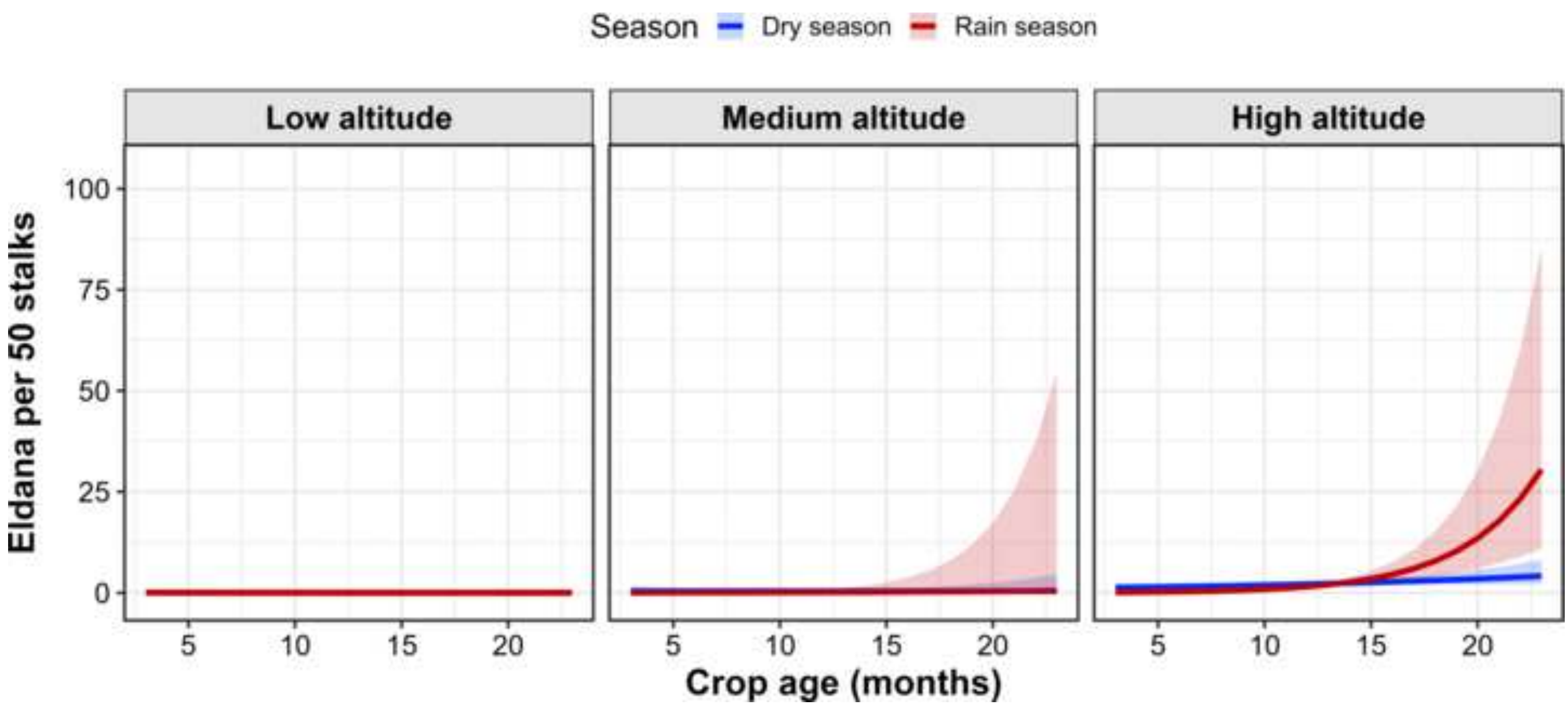

Figure

Click here to access/download;Figure;Fig 3.tif

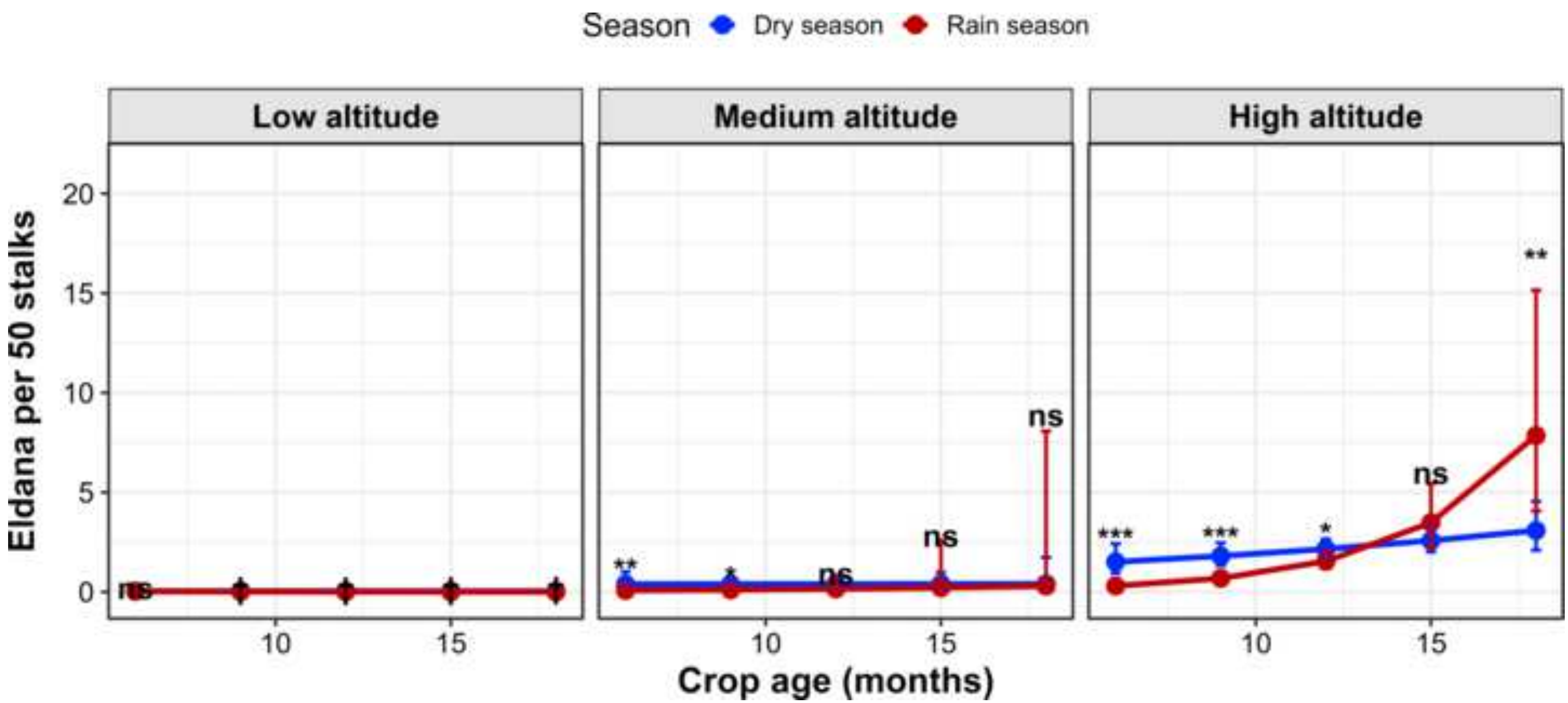

Figure

Click here to access/download;Figure;Fig 4.tif

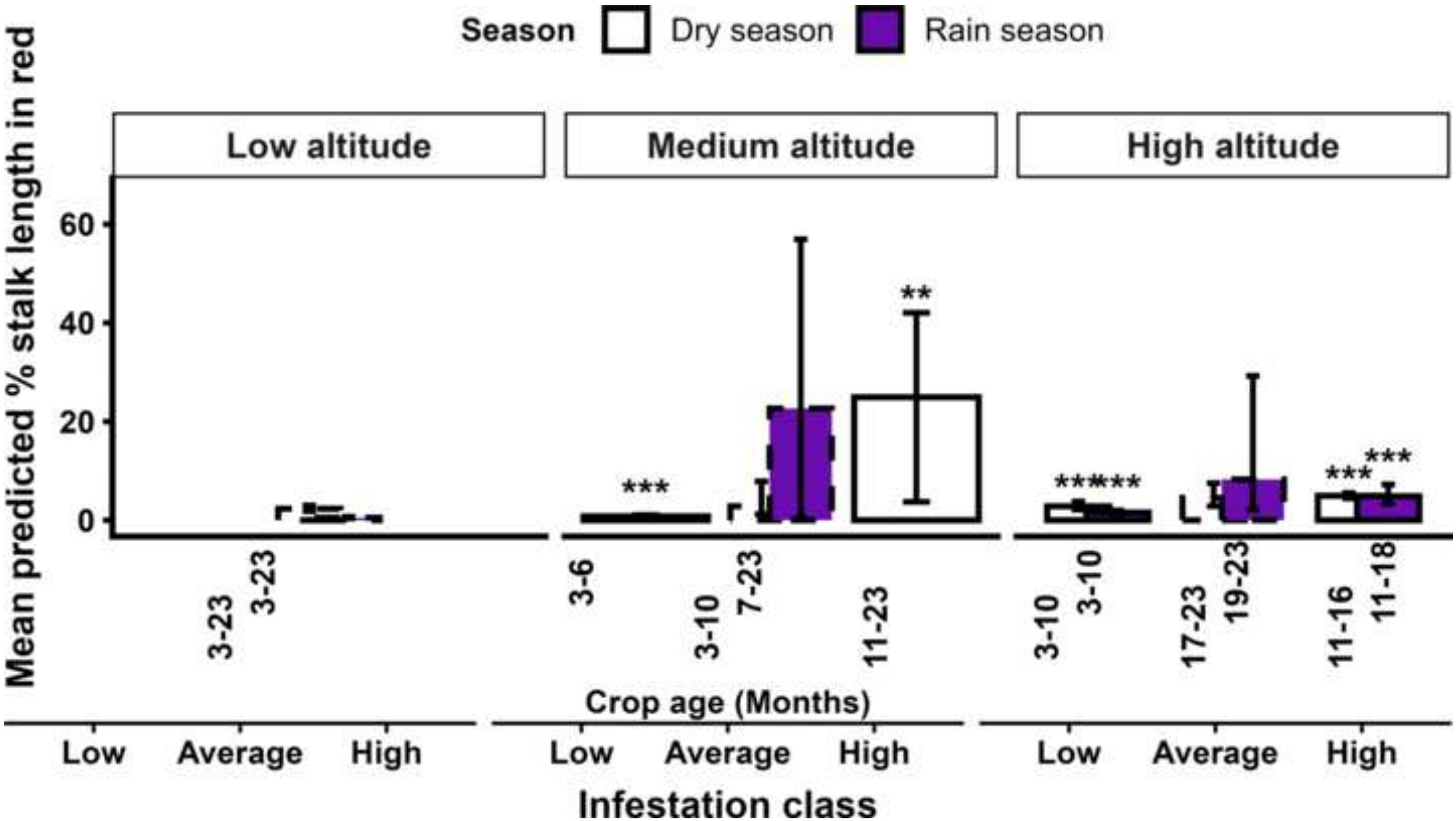

Figure

Click here to access/download;Figure;Fig 5.tif

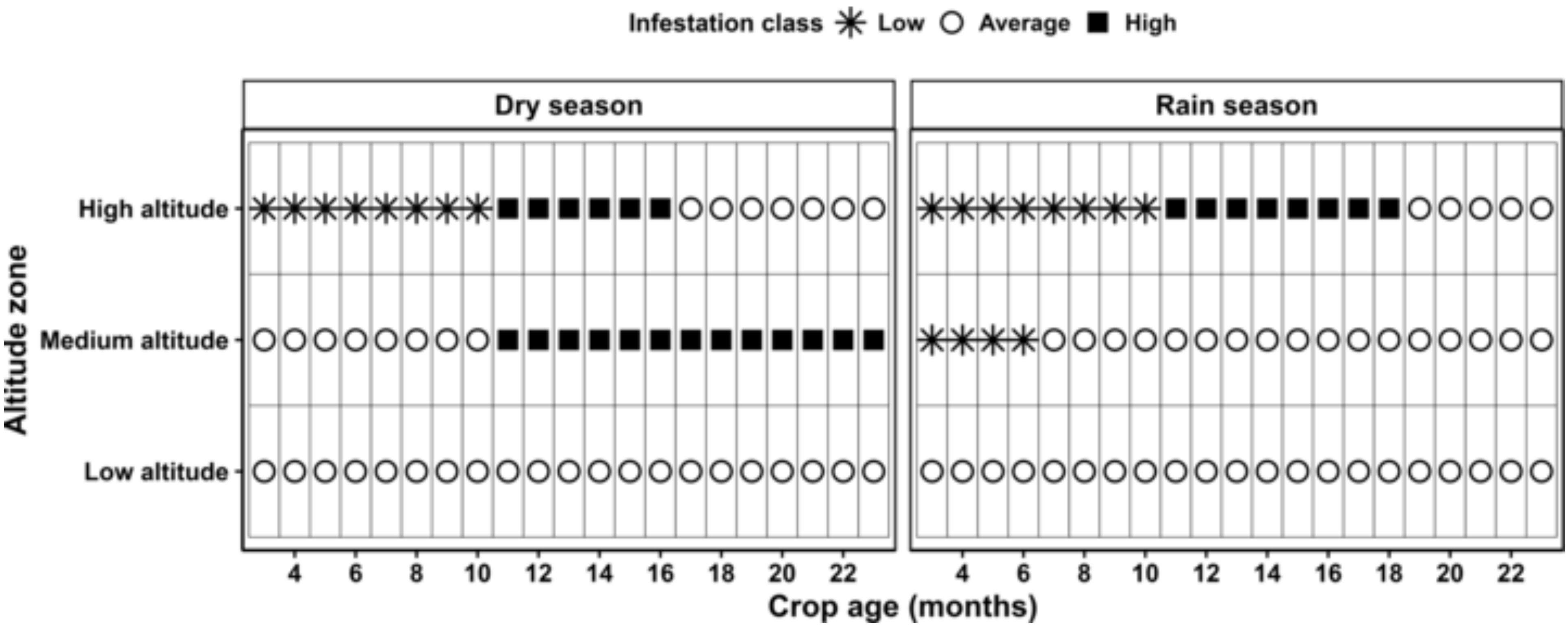

Figure

Click here to access/download;Figure;Fig 6.tif

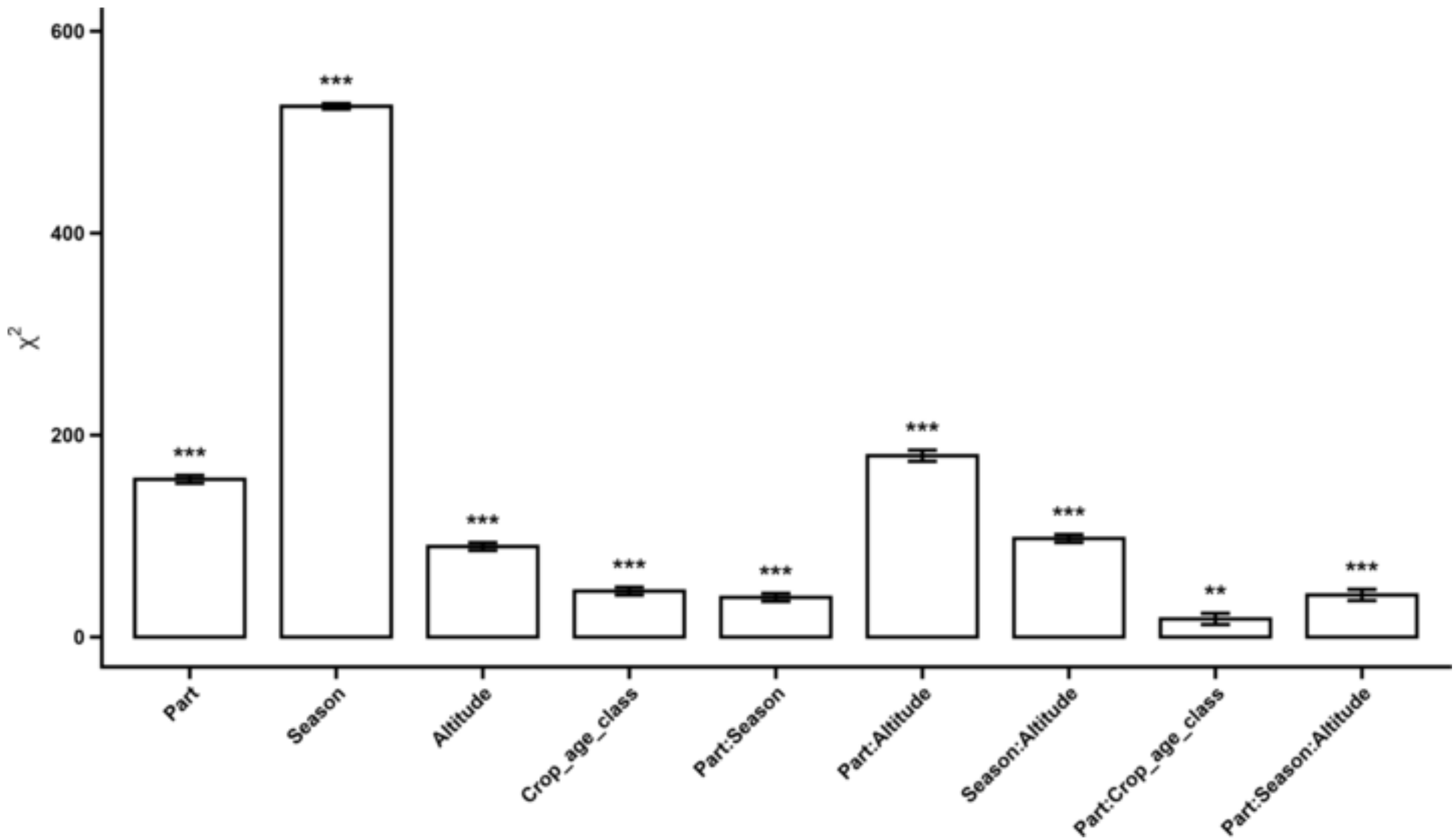

Click here to access/download;Figure;Fig 7.tif

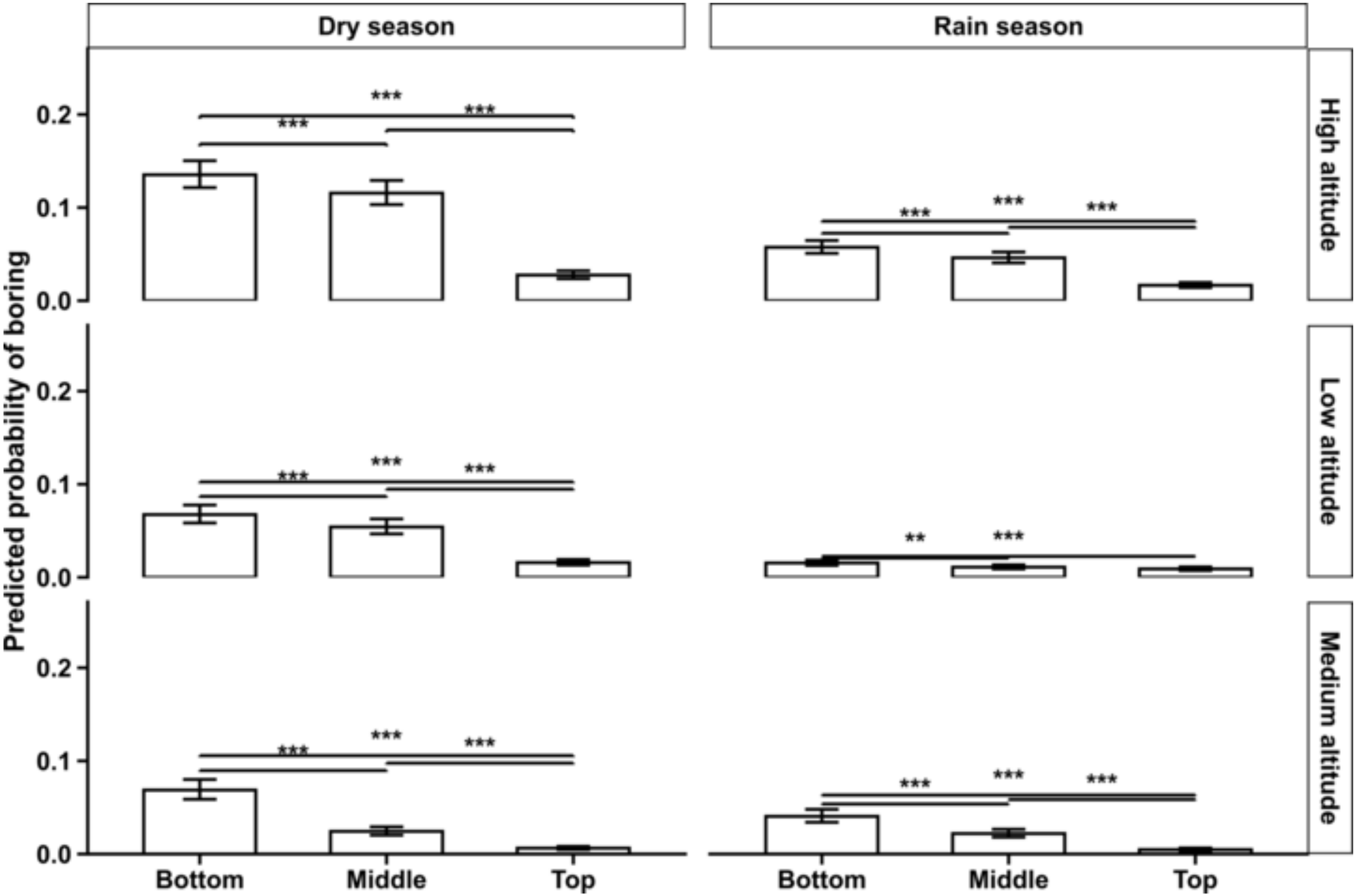

Click here to access/download;Figure;Fig 8.tif

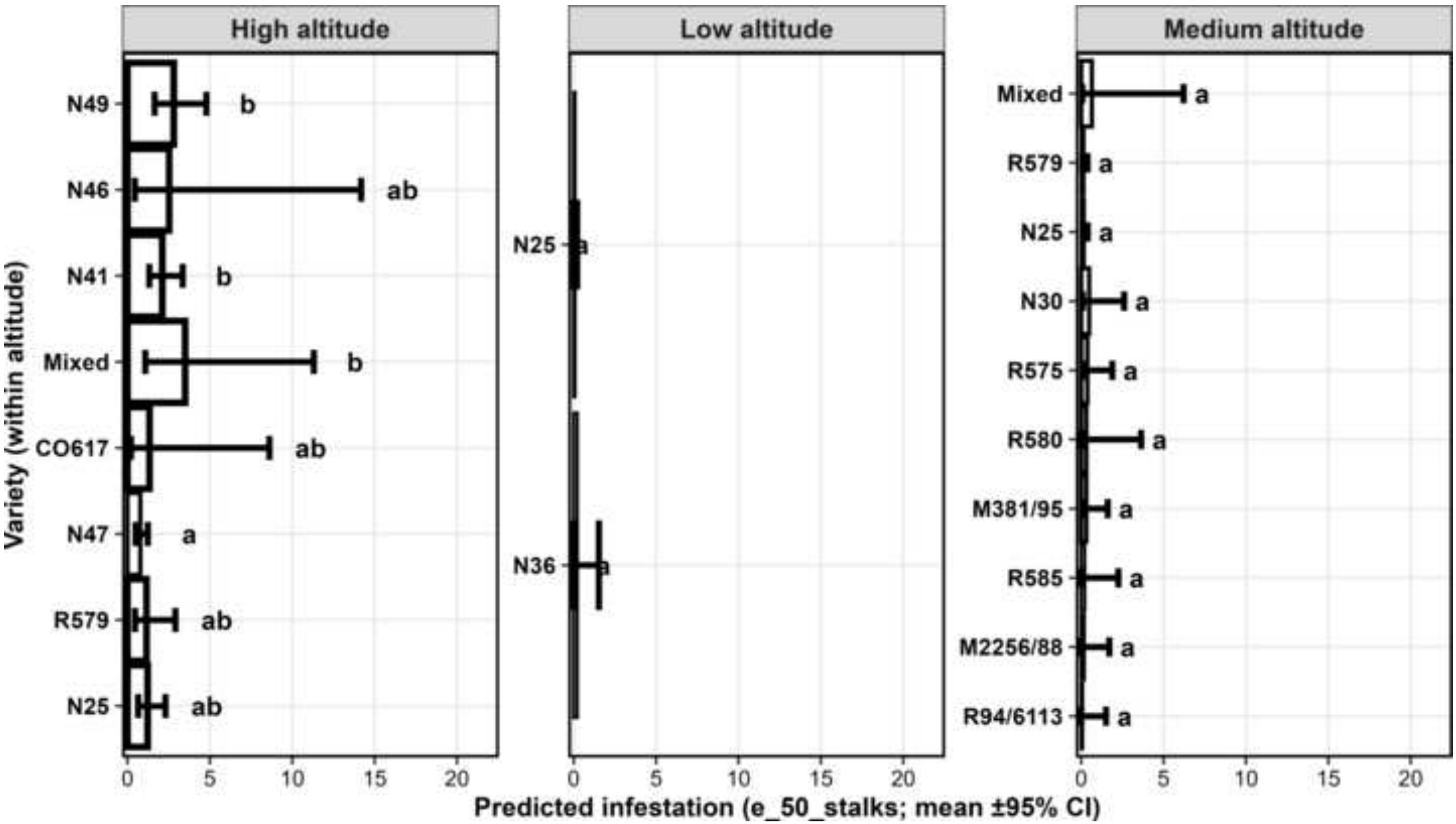

Click here to access/download
Supporting Information - Compressed/ZIP File Archive
S1_Table.pdf
