# Supplementary material for: Factors affecting the infestation status of African sugarcane stalk borer (Eldana saccharina Walker) (Lepidoptera: Pyralidae) in Tanzania
Source: PLoS One. 2026 Aug 31;21(8):e0355844. doi: 10.1371/journal.pone.0355844 (PMC13528964; doi:10.1371/journal.pone.0355844)
Supplement: Supplementary file 5 [file pone.0355844.s005.docx]

The Academic Editor and Reviewers

PLOS ONE

Dear academic editor and reviewers

**RE: AUTHOR’S RESPONSES TO THE RAISED EDITOR AND REVIEWERS’ COMMENTS**

The authors for the manuscript titled “**Factors affecting the infestation status of African sugarcane stalk borer (*Eldana saccharina* Walker) (Lepidoptera: Pyralidae) in Tanzania”** sincerely thank the reviewers for their careful evaluation of our manuscript and for the constructive comments that have helped us improve the manuscript. The following are the authors responses to the editor’s and reviewers’ comments;

# Changes to financial disclosure

I would like to make a slight change to the financial disclosure; The statement should now read as follows “This research was supported by the Mwalimu Nyerere University of Agriculture and Technology (MNUAT) internal funds. The funder had no role in the study design, data collection and analysis, decision to publish, or preparation of the manuscript

# Abstract

The comments have helped us improve the abstract and clarity of the methodological description. The authors have revised the abstract and relevant sections of the manuscript accordingly. The point-by-point responses are provided below.

1. The abstract does not clearly describe the methodological approach, and the consistency between the statistical analyses and the conclusions should be improved.

**Response:** The abstract has been revised to state more clearly that the study was a stratified multistage cross-sectional survey. We also specified that the study was conducted in major sugarcane areas of Tanzania across three altitude classes representing low, medium, and high altitudes. The authors also stated that a total of 711 blocks were surveyed, and that the infestation was analyzed using generalized linear mixed models with likelihood ratio tests and Type III Wald $X^{2}$ tests. We also revised the concluding statements to ensure that they reflect the observed statistical associations and do not overstate inference.

2. It is not sufficiently clear from the abstract how many treatments were included in the study

**Response:** The authors do appreciate this observation. Because this study was observational rather than experimental, no imposed treatments were applied. We have clarified this by describing the study as a field survey conducted across defined explanatory factors namely season, altitude, crop age, and sugarcane variety, rather than treatments.

3. The term “crop age” is commonly used for cutting ages, not for different months of growth

**Response:** This also is an important comment. In this study, “crop age” referred to the chronological age of the standing cane in months at the time of sampling, rather than cutting or harvest age. To avoid ambiguity, we retained the term but clarified it explicitly in the manuscript as “crop age (months at sampling)” in the materials and methods section and when appropriate in the results

4. In the phrase “*E. saccharina* infestation was assessed using larvae per 50 stalks alongside other damage parameters”, how many larvae were assessed?

**Response:** The authors agree that this wording was unclear. No fixed Number of larvae was assumed. Rather, infestation was quantified as the total number of *E. saccharina* larvae recovered from 50 randomly sampled stalks within each sampled block. The sentence has been revised accordingly in the abstract.

# Introduction

**Reviewer 4 comment:**

1. The introduction would benefit from the inclusion of background information on sugarcane production in Tanzania. Providing information on the importance of the crop, the extent of cultivation, and its economic relevance in the country would help to better justify the study and place the results in an appropriate context

**Response:** The authors agree that the introduction required more Tanzania-specific background to better justify the study. We have therefore revised the introduction to include brief information on the importance of sugarcane in Tanzania, its extent of cultivation, and its economic relevance. These additions strengthen the study background and better situate the significance of *E. saccharina* in the Tanzania sugarcane production systems.

**Reviewer 5 comment**

1. Line 56 – 58 Has the type of management used for sugarcane affected the presence and spread of pests?

**Response:** Thank you for this important comment. The statement has been revised to acknowledge that agronomic management can influence *E. saccharina* infestation. Specifically, the revised text now clarifies that excessive nitrogen application may increase infestation by improving host suitability for larval development, particularly under moisture-stressed conditions, whereas moisture-conserving practices may reduce infestation by maintaining crop vigor and reducing plant stress. The statement has also been expanded to mention other relevant management factors including varietal resistance, carry-over crop management, residue and stubble handling, and habitat-management practices. These revisions have been incorporated in the introduction.

2. Line 58 – 60 Are there other sites where *E. saccharina* behaves in this way (documented)? In introduction, it is necessary to mention the type of agronomic management that is done to sugarcane in the area.

**Response:** The authors agree that the original wording may have overgeneralized the evidence. Our intention was to indicate that seasonal infestation patterns of *Eldana saccharina* may vary across agroecological settings. We have therefore revised the sentence to avoid implying that this rainy-season pattern is widely documented across multiple sites unless specifically supported by published evidence. The revised text now presents this as a context-dependent pattern rather than a broad generalization.

# Materials and methods

**Reviewer 2 comments:**

1. Correct temporal discrepancies — Verify and correct the survey year (2024 or 2025) and ensure all citations have accurate publication years.

**Response:** The manuscript has been carefully checked and the survey year has been corrected throughout the text to ensure consistency. All in-text citations and reference-list entries have also been reviewed to verify that publication years are accurate and consistent between the manuscript text and the reference section. These corrections have been incorporated into the revised manuscript

2. Add a flow diagram of sampling protocol

**Response:** A flow diagram illustrating the sampling protocol has been included to improve clarity and facilitate understanding of the study design, block selection, and within-block sampling process.

3. Clarify equation (3) and (4) with correctly matched variables

**Response**: Equation 3 and 4 and their accompanying definitions have been revised to ensure that the modeled parameters correctly match the corresponding response variables. Specifically, equation 3 now refers to the expected percentage of stalk length reddened, whereas equation 4 refers to the expected proportion of internodes bored.

**Reviewer 4 comments:**

1. I consider that it would be highly valuable to present this information in a more organized manner, so that the number of samples evaluated for each variety (currently not specified in the Materials and Methods section), as well as cutting age, altitude, company, and other relevant details, are clearly indicated. This would facilitate a better understanding and interpretation of the results.

**Response:** The materials and methods section has been revised to provide more detailed information on the study areas. In addition, table summarizing the main characteristics of each study site and a map showing their geographical locations have been included to improve clarity and to assist readers in understanding the environmental differences among the study zones.

2. I consider that it would be highly valuable to present this information in a more organized manner, so that the number of samples evaluated for each variety (currently not specified in the Materials and Methods section), as well as cutting age, altitude, company, and other relevant details, are clearly indicated. This would facilitate a better understanding and interpretation of the results.

**Response:** The sampling and sampling design section has been revised to clarify the observational nature of the study and the block-base sampling framework. The revised text now states more explicitly that sugarcane blocks were the primary sampling units and that blocks were randomly selected within each estate to ensure spatial coverage. Its also clarifies that crop age and sugarcane variety were not used as pre-stratification criteria, but were recorded as characteristics of the randomly selected commercial blocks during field assessment. This explains why the distribution of crop ages and varieties was not uniform across altitude classes. These revisions improve transparency of the sampling framework and facilitate interpretation of the results.

3. Line 106 – 109 Do the rainy seasons coincide with the infestation seasons? It would be better to explain the relationship between E. saccharina and the environment (rainfall), relating the insect's life stages, sugarcane growth, and the amount of precipitation in each location.

**Response:** The paragraph has been revised to clarify that the rainy and dry seasons used in the survey were defined according to the local rainfall patterns of each altitude classes and were not assumed a priori to coincide with peak infestation periods. The revised text now explains that precipitation influences sugarcane growth, crop moisture status, and plant stress, which in turn affect host suitability for *E. saccharina* oviposition, larval establishment, survival, and internal stalk damage. It also clarifies that rainfall amount and seasonal distribution differ among locations and locally defined rainy and dry periods were therefore used to compare infestation patterns across the three study zones.

# Results

**Reviewer 1 comments**

1. Why variety is not included as a fixed factor in the model and likelihood ratio test table?

**Response:** The inclusion of sugarcane variety in the main generalized linear mixed model was initially explored alongside season, altitude, crop age, and their interactions. However, the variety variable was highly unbalanced across the study sites, with several varieties occurring only within a single altitude zone or represented by very few observations. As a result, including variety in the full ecological model produced rank-deficient design matrices, convergence problems, and unstable parameter estimates, which reduced the reliability of statistical inference. Therefore, variety was excluded to maintain model stability and interpretability. The influence of sugarcane variety was therefore assessed separately using a nested analytical framework, where varieties were compared within their respective altitude zones.

This approach provided a more appropriate ecological description of varietal differences while avoiding confounding between variety and altitude due to the unbalanced distribution of varieties across attitudes. The methods and results sections have been revised to clarify this analytical decision

2. Table 5. Why seasonal
3. Table 5. Why was the contrast column was included while it has the same information?

**Response:** The table was intended to present the seasonal contrast between dry and rain seasons within each altitude class. The caption has been revised to make this clearer, and the term “across crop ages” has been changed to “averaged over crop age” because crop age was included in the model but not displayed as a separate grouping factor in the table. The “contrast” column has also been removed because all rows represented the same comparison, dry season-rain season. The information is now provided in the table caption to avoid redundancy.

**Reviewer 2 comments**

1. Results section Restructuring -- Combine Results 3.1 and 3.3, as they address related infestation metrics.

**Response:** The infestation count and percentage stalk-bored results have been merged into a single subsection because both represent closely related measures of stalk-boring incidence. The revised section now reports the likelihood ratio test results once, followed by post-hoc and predicted-response outputs to explain the significant $season X altitude X crop age$ interaction. The percentage stalk-bored results were used to show how the observed infestation pattern translated into visible stalk damage across altitude class. The seasonal contrast has also been clarified as dry season-rainy season, with negative estimates indicating higher predicted boring during the rainy season.

2. Provide full model output including random effect variances

**Response:** Full model output has now been added to the results section. In addition to the likelihood-ratio test table, the revised results now include the negative-binomial dispersion parameter, convergence information, Akaike Information Criterion (AIC), Bayesian Information Criterion, Random effect variance, and standard deviation.

3. Conduct sensitivity analysis to justify n=50

**Response:** A count-based sensitivity analysis has now been added to justify the 50-stalk sampling protocol. Because the main response variable was *Eldana saccharina* larval count per 50 stalks, the sensitivity analysis was conducted on the count scale using the fitted negative binomial distribution. The analysis compared detection probability and expected relative variability under the observe 50-stalk and a hypothetical 100-stalk sampling protocols. The results showed that 50-stalk sampling was less sensitive when expected infestation was very low, particularly below 1-2 individuals per 50 stalks. However, detection probability increased rapidly with expected count and 93% at 5 individuals per 50 stalks, 97.9% at 10 individuals per 50 stalks, and 99.5% at 20 individuals per 50 stalks. The manuscript has therefore been revised to clarify that the 50-stalk sampling protocol was adequate for detecting moderate to high infestation levels and for comparing field-level patterns among altitudes, seasons, and crop ages, while acknowledging possible under-detection of very low or highly localized infestation.

4. Validation data for damage assessment (e.g., correlation between red stalk length and larval counts

**Response:** The authors now included an internal validation analysis of the damage assessment. Spearman’s rank correlation was used to test the association between *E. saccharina* larval counts per 50 stalks and measured stalk damage indicators. The analysis indicated positive and significant correlations between larval counts and percentage stalk length red (ρs = 0.61, P < 0.001), total red stalk length (ρs = 0.6, P < 0.001), percentage stalks bored (ρs = 0.56, P < 0.001), and total number of bored internodes (ρs = 0.56, P < 0.001). These findings support the biological consistency of the damage assessment. The manuscript has been revised to include this analysis and clarify that damage indicators represent cumulative feeding injury, while larval counts represent infestation detected at the time of sampling.

**Reviewer 3 comments**

5. This manuscript provides a useful assessment of *Eldana saccharina* infestation across three zones in Tanzania. While the study is well-structured and offers informative data, but it lacks the broader context required for a global audience. The scope of the study is limited to a survey of infestation status; it does not quantify the actual economic losses caused by *E. saccharina* in the surveyed locations. The manuscript fails to connect these findings to practical management strategies or discuss how the current data can be utilized to improve pest control in the region. Apart from this, the manuscript would benefit significantly from the inclusion of high-quality images demonstrating the natural incidence of E. saccharina in the field.

**Response:** The manuscript has been revised to provide broader context on the importance of *E. saccharina* infestation in sugarcane production systems and clarify the relevance of the Tanzanian survey for pest surveillance and regional management planning. Additional text has been included in the discussion to explain how the observed variation across altitudes, seasons, and crop ages can guide practical monitoring, particularly by prioritizing older crops and high-risk altitude production zones.

The limitation regarding economic losses has also been addressed. The revised manuscript now clearly states that yield loss, sucrose reduction, and monetary losses were not directly quantified; therefore, the results should be interpreted as indicators of infestation risk and stalk-boring incidence rather than direct economic-loss estimates. Future studies linking infestation levels with yield and cane-quality data have been recommended.

Field images of natural *E. saccharina* incidence were taken during the survey. However, after reviewing them, the available images were not of sufficient quality for inclusion in a peer-reviewed publication. To avoid compromising the visual quality of the manuscript, they were not included.

**Reviewer 4 comments**

1. It is difficult to interpret the results without an integrated analysis of all the data. How many observations or determinations were performed for each crop age?

**Response:** The manuscript has been revised to clarify the structure of dataset and the integrated modelling approach used in the analysis. Crop age was treated as a continuous covariate in the generalized linear mixed models rather than as a categorical treatment. Therefore, all observations were analyzed jointly in models that included season, altitude, crop age, and their interactions. This approach allowed the effect of crop age to be estimated while accounting for seasonal and altitudinal variation.

The materials and methods have been revised to clarify that the same identified blocks were assessed during both the rainy and dry seasons. Each observation represented one block-season determination based on 50 sampled sugarcane stalks. Because the survey was conducted under commercial field conditions, planting dates differed among estates and blocks.

To improve transparency, the results section now refers to a supplementary table showing the distribution of observations across ages, seasons, and the corresponding number of stalks assessed for each crop age category. This revision allows readers to evaluate the sampling support underlying the crop-age effects reported in the model.

2. In addition, how does the life cycle of the borer vary across the different altitudes, and was its potential influence over altitude on the results considered?

**Response:** The discussion section has been revised to address the potential influence of altitude on the biology of *Eldana saccharina*. The revision now explains that altitude-related differences in temperature, relative humidity, and crop phenology may influence larval development, adult emergence, reproduction, survival, and seasonal population build-up. It has also been clarified that life-cycle parameters were not directly measured in the present study; therefore, the explanation is presented as biologically plausible but inferential. A recommendation has been added for future studies to quantify life-cycle parameters across altitudes under Tanzania sugarcane-growing conditions.

3. Furthermore, how are the varieties distributed among the sampling sites? Since different varieties may exhibit distinct responses, it would be important to clarify how varietal distribution could have influenced the observed results.

**Response:** The manuscript has been revised particularly in the data analysis subsection to clarify the distribution of sugarcane varieties across the sampling sites and altitudes. Because the study was conducted under commercial production conditions, varieties were not equally distributed among all sites; rather, varietal occurrence reflected the planting decisions and production practices of the respective sugarcane-producing company.

To address this concern statically, varieties were analyzed within their respective altitude zones by nesting variety within altitude. This approach was used because several varieties were not represented across all altitudes, making it inappropriate to treat variety as a fully crossed factor across the entire dataset. The model used for this analysis has now been added to the materials and methods section for clarity.

The revised manuscript also acknowledges that varietal differences may have contributed to variation in *Eldana saccharina* infestation. However, because some varieties were altitude-or estate-specific, variety effects could not be fully separated from altitude-related factor, while season, altitude, and crop age remain the main field-level predictors evaluated in the integrated model.

**Reviewer 5 comments**

Line 200 - 201 There is a very strong relationship between climate and sugarcane production. Line 212-214 Do the rainfall values match the insect's environmental requirements? The results are well described, but it is difficult to understand the population variations of the insect versus the environmental conditions, since it is not known whether the damage caused by the insect to the plant is normal.

**Response:** The manuscript has been revised to clarify that the damage recorded during the survey represented natural field incidence of *E. saccharina* infestation, rather than experimentally induced injury. The data collection section now describes the field symptoms used to confirm infestation, including bored stalks, internal stalk tissue red, and the presence of larvae and/or pupae within sugarcane stalk. A corresponding statement has also been added to the results section 3.1 to make clear that the reported infestation patterns were based on confirmed field symptoms of *E. saccharina*

The discussion has also been revised to clarify the role of environmental conditions. Rainfall was not treated as a direct threshold requirement for *E. saccharina* development because life-cycle parameters were not directly measured in this study. Instead, rainfall, season, temperature, relative humidity, altitude, and crop age are discussed as field-level conditions that may influence infestation risk and population build-up.

# Discussion

**Reviewer 2 comments**

1. Discuss potential bias from estate restrictions in limitations section

**Response:** The limitations section has been revised to discuss the potential bias introduced by estate-level restrictions on destructive sampling. Because the study was conducted in commercial sugarcane estates, the number of stalks that could be destructively sampled was limited by estate management. Although the SASRI protocol recommends 100 stalks per 5 ha, estate restrictions allowed only 50 stalks per block in the present study.

The revised manuscript now acknowledges that this restriction may have reduced the probability of detecting very low or highly localized *Eldana saccharina* infestations and may have contributed to underestimation of infestation in some fields. However, the same sampling intensity and field procedure were applied consistently across all sampled blocks, seasons, and altitudes, which allowed standardized comparison among study sites. The limitation has therefore been clarified, and future studies are recommended to use larger sample size.

2. Clarify contradictory findings — Expand discussion on why high-altitude rainy season (moist) and low-altitude dry season (stressed) both show elevated damage, possibly through different mechanisms.

**Response:** The discussion section has been revised to clarify that the observed patterns are not necessarily contradictory because infestation counts and internal stalk tissue damage represent related but different aspects of the *Eldana saccharina*-sugarcane interaction. Infestation counts reflect the number of larvae detected at time of sampling, whereas internal stalk tissue damage may represent cumulative feeding injury that persist after larval movement.

The revised discussion now explains that greater infestation at high altitude during the rainy season may reflect favorable seasonal moisture, suitable temperature, and relative humidity, older crop age, and prolonged exposure to oviposition and larval establishment, the relatively greater internal tissue damage recorded at low altitude during the dry season may reflect plant stress-related susceptibility, reduced stalk vigor, and the persistence of earlier feeding damage even when live pest counts are low. Therefore, high-altitude rainy-season infestation and low-altitude dry-season damage may arise through different mechanisms; one mainly through pest population build-up under favorable conditions, and the other through increased plant vulnerability and accumulated damage under stress conditions.

**Reviewer 5 comments**

1. Line 301 – 311 What quantity is considered "high infestation" or "low infestation"? Are there any international records for economic damage thresholds? If so, please mention them.

**Response:** The manuscript has been revised to avoid using qualitative terms such as “high infestation”, “low infestation”, and “elevated boring intensity” without numerical context. Where these terms are retained, they are now linked to the observed or model-predicted infestation values reported in the results.

2. Line 312 – 316 Are there no more reports? It is one of the most important phytosanitary problems on the African continent; are there no further studies on the insect's biological behavior?

**Response:** The discussion has been revised to include additional reports on *Eldana saccharina* infestation and biological behavior. The revised text now goes beyond field observations from northern Ivory Coast and South Africa by incorporating studies on oviposition behavior, egg hatchability, larval feeding, fecundity, mating biology, temperature-dependent development, adult water-balance traits, and climate-related population phenology.

3. Line 312 How long does the sugarcane last before the harvest? More than 12 months?

**Response:** The manuscript has been revised to clarify the crop-age range represented in the study. crop age was recorded as months after planting at the time of sampling. The surveyed commercial fields included both younger, standard-age and older crops, including fields older than 12 months before harvest. This clarification has been added because crop age is important for interpreting *Eldana saccharina* infestation, as older cane remains exposed to oviposition and larval establishment for a longer period and has more developed stalk tissues in which internal feeding damage can accumulate.

4. Line 346 – 348 What characteristics do these varieties have that make them more susceptible?

**Response:** The discussion section has been revised to provide a more specific explanation of documented varietal characteristics that may be associated with *Eldana saccharina* susceptibility. The revised text now clarifies that N49 has previously been reported as susceptible to *E. saccharina* under South African production conditions, with carry-over in irrigated areas not recommended. Its documented characteristics include very high sucrose content, moderate fibre content, and possible lodging under higher-tonnage conditions. For N41, the revision now presents the evidence more cautiously, because pervious reports indicate high Eldana levels under some production conditions.

The revised discussion also explains that resistance or susceptibility of N41 and N49 to *E. saccharina* has been associated in previous studies with traits such as internode rind hardness, bud-scale properties, fibre content, leaf-sheath traits, stalk surface wax chemistry, flavonoids, and silicon-mediated defense. However, because these traits were not directly quantified in the present study, the manuscript now interpret the greater infestation in N41 and N49 containing fields as a field-level association rather than direct evidence of the exact susceptibility mechanism.

5. Line 357 How many months are early harvests?

**Response:** The manuscript has been revised to define what was meant by “early harvesting.” In the context of the present study, this does not refer to harvesting immature cane, but rather to prioritizing the harvest of matured or near-matured fields before prolonged carry-over, particularly when crops approach or exceed 11-12 months after planting in medium and high altitude areas. The revised text now uses “timely harvest scheduling” instead of the more general term “early harvesting.”

Authors sincerely thank the Academic Editor and reviewers for their constructive comments and valuable suggestions, which have helped us improve the clarity and overall quality of the manuscript. We have carefully addressed each comment and revised the manuscript accordingly.

Your sincerely;

Hamis Daniel Wambura

On behalf of all co-authors

hamisdaniel@gmail.com
