# Supplementary material for: Factors affecting the infestation status of African sugarcane stalk borer (Eldana saccharina Walker) (Lepidoptera: Pyralidae) in Tanzania
Source: PLoS One. 2026 Aug 31;21(8):e0355844. doi: 10.1371/journal.pone.0355844 (PMC13528964; doi:10.1371/journal.pone.0355844)
Supplement: Peer review history

# Peer review: Decision Letter 0

**DOI:** 10.1371/journal.pone.0355844.r001

**Authors:** Noé Aguilar-Rivera

23 Jun 2026

Factors affecting the infestation status of African sugarcane stalk borer (Eldana saccharina Walker) (Lepidoptera: Pyralidae) in Tanzania

PLOS One

Dear Dr. Wambura,

Thank you for submitting your manuscript to PLOS ONE. After careful consideration, we feel that it has merit but does not fully meet PLOS ONE’s publication criteria as it currently stands. Therefore, we invite you to submit a revised version of the manuscript that addresses the points raised during the review process.

Please submit your revised manuscript by Jul 22 2026 11:59PM. If you will need more time than this to complete your revisions, please reply to this message or contact the journal office at plosone@plos.org. When you're ready to submit your revision, log on to https://www.editorialmanager.com/pone/ and select the 'Submissions Needing Revision' folder to locate your manuscript file.

Please include the following items when submitting your revised manuscript:

- A letter that responds to each point raised by the academic editor and reviewer(s). You should upload this letter as a separate file labeled 'Response to Reviewers'.
- A marked-up copy of your manuscript that highlights changes made to the original version. You should upload this as a separate file labeled 'Revised Manuscript with Track Changes'.
- An unmarked version of your revised paper without tracked changes. You should upload this as a separate file labeled 'Manuscript'.

If you would like to make changes to your financial disclosure, please include your updated statement in your cover letter. Guidelines for resubmitting your figure files are available below the reviewer comments at the end of this letter.

If applicable, we recommend that you deposit your laboratory protocols in protocols.io to enhance the reproducibility of your results. Protocols.io assigns your protocol its own identifier (DOI) so that it can be cited independently in the future. For instructions see: https://journals.plos.org/plosone/s/submission-guidelines#loc-laboratory-protocols. Additionally, PLOS ONE offers an option for publishing peer-reviewed Lab Protocol articles, which describe protocols hosted on protocols.io. Read more information on sharing protocols at https://plos.org/protocols?utm_medium=editorial-email&utm_source=authorletters&utm_campaign=protocols.

As the corresponding author, your ORCID iD is verified in the submission system and will appear in the published article. PLOS supports the use of ORCID, and we encourage all coauthors to register for an ORCID iD and use it as well. Please encourage your coauthors to verify their ORCID iD within the submission system before final acceptance, as unverified ORCID iDs will not appear in the published article. *Only*  the individual author can complete the verification step; PLOS staff *cannot*  verify ORCID iDs on behalf of authors.

We look forward to receiving your revised manuscript.

Kind regards,

Noé Aguilar-Rivera

Academic Editor

PLOS One

Journal Requirements:

When submitting your revision, we need you to address these additional requirements.

1.Please ensure that your manuscript meets PLOS ONE's style requirements, including those for file naming. The PLOS ONE style templates can be found at

https://journals.plos.org/plosone/s/file?id=wjVg/PLOSOne_formatting_sample_main_body.pdf and

https://journals.plos.org/plosone/s/file?id=ba62/PLOSOne_formatting_sample_title_authors_affiliations.pdf

2. Please include your full ethics statement in the ‘Methods’ section of your manuscript file. In your statement, please include the full name of the IRB or ethics committee who approved or waived your study, as well as whether or not you obtained informed written or verbal consent. If consent was waived for your study, please include this information in your statement as well.

3. If the reviewer comments include a recommendation to cite specific previously published works, please review and evaluate these publications to determine whether they are relevant and should be cited. There is no requirement to cite these works unless the editor has indicated otherwise.

[Note: HTML markup is below. Please do not edit.]

Reviewers' comments:

Reviewer's Responses to Questions

**Comments to the Author**

1. Is the manuscript technically sound, and do the data support the conclusions?

Reviewer #1: Yes

Reviewer #2: Partly

Reviewer #3: Partly

Reviewer #4: No

Reviewer #5: Partly

**********

2. Has the statistical analysis been performed appropriately and rigorously?

Reviewer #1: Yes

Reviewer #2: Yes

Reviewer #3: Yes

Reviewer #4: No

Reviewer #5: Yes

**********

3. Have the authors made all data underlying the findings in their manuscript fully available?

The PLOS Data policy

Reviewer #1: Yes

Reviewer #2: Yes

Reviewer #3: Yes

Reviewer #4: No

Reviewer #5: Yes

**********

4. Is the manuscript presented in an intelligible fashion and written in standard English?

Reviewer #1: No

Reviewer #2: No

Reviewer #3: Yes

Reviewer #4: No

Reviewer #5: Yes

**********

Reviewer #1: The manuscript addresses an important issue related to sugarcane pest ecology and provides valuable information on the influence of ecological parameters on Eldana saccharina infestation in Tanzania. The study is comprehensive, statistically supported, and relevant for integrated pest management strategies. However, the manuscript requires substantial language editing, clarification of methodology, improvement in statistical presentation, and refinement of discussion and interpretation before it can be considered for publication.

Reviewer #2: The study addresses a relevant pest management question with appropriate spatial scope, but methodological transparency issues, statistical reporting gaps, and temporal/citation inconsistencies prevent acceptance in current form. The core findings are potentially valuable, particularly the altitude × season × age interaction, but the manuscript requires following revisions:

Correct temporal discrepancies — Verify and correct the survey year (2024 or 2025) and ensure all citations have accurate publication years.

Revise statistical presentation:

a) Clarify equation (3) and (4) with correctly matched variables

b) Provide full model output including random effect variances

c) Add a table of post-hoc comparisons for the three-way interaction

Address sampling limitations:

a) Conduct sensitivity analysis to justify n=50

b) Discuss potential bias from estate restrictions in limitations section

Improve figure integration — Embed figures within manuscript or provide a consolidated figure file with clear captions.

Clarify contradictory findings — Expand discussion on why high-altitude rainy season (moist) and low-altitude dry season (stressed) both show elevated damage, possibly through different mechanisms.

Also do the following additions for better understandings of the study:

a) Map of study sites showing altitude zones and company locations

b) Flow diagram of sampling protocol

c) Validation data for damage assessment (e.g., correlation between red stalk length and larval counts)

d) Results section Restructuring -- Combine Results 3.1 and 3.3, as they address related infestation metrics

Reviewer #3: This manuscript provides a useful assessment of Eldana saccharina infestation across three zones in Tanzania. While the study is well-structured and offers informative data, but it lacks the broader context required for a global audience.

The scope of the study is limited to a survey of infestation status; it does not quantify the actual economic losses caused by E. saccharina in the surveyed locations. The manuscript fails to connect these findings to practical management strategies or discuss how the current data can be utilized to improve pest control in the region.

Apart from this, the manuscript would benefit significantly from the inclusion of high-quality images demonstrating the natural incidence of E. saccharina in the field.

I believe the current submission requires substantial improvements to meet the standards of PLOS ONE. The current version does not sufficiently bridge the gap between survey data and global scientific impact.

Reviewer #4: Abstract

No major concerns are evident from the structure of the abstract. However, a careful evaluation of the Materials and Methods section and the consistency between the statistical analyses and the conclusions drawn is recommended.

The methodological approach should be described more clearly, and the results should be interpreted more thoroughly to ensure that the conclusions are adequately supported. In addition, it is not sufficiently clear from the abstract how many treatments were included in the study.

Introduction

The Introduction would benefit from the inclusion of background information on sugarcane production in Tanzania. Providing information on the importance of the crop, the extent of cultivation, and its economic relevance in the country would help to better justify the study and place the results in an appropriate context.

Materials and methods

More detailed information on the study areas should be provided to facilitate the interpretation of the results. Including a Table summarizing the main characteristics of each site and/or a map showing their geographical locations would greatly improve the clarity of the manuscript and help readers better understand the environmental differences among the study zones.

I consider that it would be highly valuable to present this information in a more organized manner, so that the number of samples evaluated for each variety (currently not specified in the Materials and Methods section), as well as cutting age, altitude, company, and other relevant details, are clearly indicated. This would facilitate a better understanding and interpretation of the results.

Please note that the term crop ages does not refer to cutting ages, but rather to the number of months after planting. I recommend revising this terminology throughout the manuscript to ensure clarity and avoid misinterpretation.

Results

It is difficult to interpret the results without an integrated analysis of all the data. How many observations or determinations were performed for each crop age? In addition, how does the life cycle of the borer vary across the different altitudes, and was its potential influence over altitude on the results considered? I suggest conducting an integrated analysis of all variables and their interactions, as this would allow more robust inferences to be made and would strengthen the conclusions drawn from the study.

Furthermore, how are the varieties distributed among the sampling sites? Since different varieties may exhibit distinct responses, it would be important to clarify how varietal distribution could have influenced the observed results.

I believe that, with a more comprehensive analysis, the results could become more relevant and provide stronger scientific insights. In its current form, it is difficult to draw robust interpretations from the data. At present, the study appears to have primarily local significance, and it may be more suitable for publication in a journal with a specific focus on entomology

Reviewer #5: Factors affecting the infestation status of African sugarcane stalk borer (Eldana saccharina Walker) (Lepidoptera: Pyralidae) in Tanzania

Abstract: Ok

Keywords: Ok

Introduction: Line 56 – 58 Has the type of management used for sugarcane affected the presence and spread of pests? Line 58 – 60 Are there other sites where E. saccharina behaves in this way (documented)? In introduction, it is necessary to mention the type of agronomic management that is done to sugarcane in the area.

Materials and methods: In the description of the study area, it is suggested to include a location map so that the reader knows where the study area is located. Line 106 – 109 Do the rainy seasons coincide with the infestation seasons? It would be better to explain the relationship between E. saccharina and the environment (rainfall), relating the insect's life stages, sugarcane growth, and the amount of precipitation in each location.

Results: Line 200 - 201 There is a very strong relationship between climate and sugarcane production. Line 212-214 Do the rainfall values match the insect's environmental requirements? The results are well described, but it is difficult to understand the population variations of the insect versus the environmental conditions, since it is not known whether the damage caused by the insect to the plant is normal.

Discussion: Line 301 – 311 What quantity is considered "high infestation" or "low infestation"? Are there any international records for economic damage thresholds? If so, please mention them. Line 312 – 316 Are there no more reports? It is one of the most important phytosanitary problems on the African continent; are there no further studies on the insect's biological behavior? Line 312 How long does the sugarcane last before the harvest? More than 12 months? Line 346 – 348 What characteristics do these varieties have that make them more susceptible? Line 357 How many months are early harvests? Further research is needed on the insect's characteristics. The description of the insect's behavior at different altitudes, ages, etc., is well described in the manuscript, but it remains to be determined whether this behavior is typical in the landscapes studied.

Conclusion: It's good, but it's very short

Reference: Ok

**********

what does this mean?). If published, this will include your full peer review and any attached files.

If you choose “no”, your identity will remain anonymous but your review may still be made public.

**Do you want your identity to be public for this peer review?**  For information about this choice, including consent withdrawal, please see our Privacy Policy

Reviewer #1: **Yes:**  Rajeev Kumar

Reviewer #2: **Yes:**  Danish Ibrar

Reviewer #3: No

Reviewer #4: No

Reviewer #5: **Yes:**  LUIS ALBERTO OLVERA-VARGAS

**********

[NOTE: If reviewer comments were submitted as an attachment file, they will be attached to this email and accessible via the submission site. Please log into your account, locate the manuscript record, and check for the action link "View Attachments". If this link does not appear, there are no attachment files.]

To ensure your figures meet our technical requirements, please review our figure guidelines: https://journals.plos.org/plosone/s/figures

You may also use PLOS’s free figure tool, NAAS, to help you prepare publication quality figures: https://journals.plos.org/plosone/s/figures#loc-tools-for-figure-preparation.

NAAS will assess whether your figures meet our technical requirements by comparing each figure against our figure specifications.

Attachment Submitted filename: PONE-D-26-23049.pdf

# Author comment: Author response to Decision Letter 1

**DOI:** 10.1371/journal.pone.0355844.r002

14 Jul 2026

The Academic Editor and Reviewers

PLOS ONE

Dear academic editor and reviewers

RE: AUTHOR’S RESPONSES TO THE RAISED EDITOR AND REVIEWERS’ COMMENTS

The authors for the manuscript titled “Factors affecting the infestation status of African sugarcane stalk borer (Eldana saccharina Walker) (Lepidoptera: Pyralidae) in Tanzania” sincerely thank the reviewers for their careful evaluation of our manuscript and for the constructive comments that have helped us improve the manuscript. The following are the authors responses to the editor’s and reviewers’ comments;

Changes to financial disclosure

I would like to make a slight change to the financial disclosure; The statement should now read as follows “This research was supported by the Mwalimu Nyerere University of Agriculture and Technology (MNUAT) internal funds. The funder had no role in the study design, data collection and analysis, decision to publish, or preparation of the manuscript.”

Abstract

The comments have helped us improve the abstract and clarity of the methodological description. The authors have revised the abstract and relevant sections of the manuscript accordingly. The point-by-point responses are provided below.

The abstract does not clearly describe the methodological approach, and the consistency between the statistical analyses and the conclusions should be improved.

Response: The abstract has been revised to state more clearly that the study was a stratified multistage cross-sectional survey. We also specified that the study was conducted in major sugarcane areas of Tanzania across three altitude classes representing low, medium, and high altitudes. The authors also stated that a total of 711 blocks were surveyed, and that the infestation was analyzed using generalized linear mixed models with likelihood ratio tests and Type III Wald X^2 tests. We also revised the concluding statements to ensure that they reflect the observed statistical associations and do not overstate inference.

It is not sufficiently clear from the abstract how many treatments were included in the study

Response: The authors do appreciate this observation. Because this study was observational rather than experimental, no imposed treatments were applied. We have clarified this by describing the study as a field survey conducted across defined explanatory factors namely season, altitude, crop age, and sugarcane variety, rather than treatments.

The term “crop age” is commonly used for cutting ages, not for different months of growth

Response: This also is an important comment. In this study, “crop age” referred to the chronological age of the standing cane in months at the time of sampling, rather than cutting or harvest age. To avoid ambiguity, we retained the term but clarified it explicitly in the manuscript as “crop age (months at sampling)” in the materials and methods section and when appropriate in the results

In the phrase “E. saccharina infestation was assessed using larvae per 50 stalks alongside other damage parameters”, how many larvae were assessed?

Response: The authors agree that this wording was unclear. No fixed Number of larvae was assumed. Rather, infestation was quantified as the total number of E. saccharina larvae recovered from 50 randomly sampled stalks within each sampled block. The sentence has been revised accordingly in the abstract.

Introduction

Reviewer 4 comment:

The introduction would benefit from the inclusion of background information on sugarcane production in Tanzania. Providing information on the importance of the crop, the extent of cultivation, and its economic relevance in the country would help to better justify the study and place the results in an appropriate context

Response: The authors agree that the introduction required more Tanzania-specific background to better justify the study. We have therefore revised the introduction to include brief information on the importance of sugarcane in Tanzania, its extent of cultivation, and its economic relevance. These additions strengthen the study background and better situate the significance of E. saccharina in the Tanzania sugarcane production systems.

Reviewer 5 comment

Line 56 – 58 Has the type of management used for sugarcane affected the presence and spread of pests?

Response: Thank you for this important comment. The statement has been revised to acknowledge that agronomic management can influence E. saccharina infestation. Specifically, the revised text now clarifies that excessive nitrogen application may increase infestation by improving host suitability for larval development, particularly under moisture-stressed conditions, whereas moisture-conserving practices may reduce infestation by maintaining crop vigor and reducing plant stress. The statement has also been expanded to mention other relevant management factors including varietal resistance, carry-over crop management, residue and stubble handling, and habitat-management practices. These revisions have been incorporated in the introduction.

Line 58 – 60 Are there other sites where E. saccharina behaves in this way (documented)? In introduction, it is necessary to mention the type of agronomic management that is done to sugarcane in the area.

Response: The authors agree that the original wording may have overgeneralized the evidence. Our intention was to indicate that seasonal infestation patterns of Eldana saccharina may vary across agroecological settings. We have therefore revised the sentence to avoid implying that this rainy-season pattern is widely documented across multiple sites unless specifically supported by published evidence. The revised text now presents this as a context-dependent pattern rather than a broad generalization.

Materials and methods

Reviewer 2 comments:

Correct temporal discrepancies — Verify and correct the survey year (2024 or 2025) and ensure all citations have accurate publication years.

Response: The manuscript has been carefully checked and the survey year has been corrected throughout the text to ensure consistency. All in-text citations and reference-list entries have also been reviewed to verify that publication years are accurate and consistent between the manuscript text and the reference section. These corrections have been incorporated into the revised manuscript

Add a flow diagram of sampling protocol

Response: A flow diagram illustrating the sampling protocol has been included to improve clarity and facilitate understanding of the study design, block selection, and within-block sampling process.

Clarify equation (3) and (4) with correctly matched variables

Response: Equation 3 and 4 and their accompanying definitions have been revised to ensure that the modeled parameters correctly match the corresponding response variables. Specifically, equation 3 now refers to the expected percentage of stalk length reddened, whereas equation 4 refers to the expected proportion of internodes bored.

Reviewer 4 comments:

I consider that it would be highly valuable to present this information in a more organized manner, so that the number of samples evaluated for each variety (currently not specified in the Materials and Methods section), as well as cutting age, altitude, company, and other relevant details, are clearly indicated. This would facilitate a better understanding and interpretation of the results.

Response: The materials and methods section has been revised to provide more detailed information on the study areas. In addition, table summarizing the main characteristics of each study site and a map showing their geographical locations have been included to improve clarity and to assist readers in understanding the environmental differences among the study zones.

I consider that it would be highly valuable to present this information in a more organized manner, so that the number of samples evaluated for each variety (currently not specified in the Materials and Methods section), as well as cutting age, altitude, company, and other relevant details, are clearly indicated. This would facilitate a better understanding and interpretation of the results.

Response: The sampling and sampling design section has been revised to clarify the observational nature of the study and the block-base sampling framework. The revised text now states more explicitly that sugarcane blocks were the primary sampling units and that blocks were randomly selected within each estate to ensure spatial coverage. Its also clarifies that crop age and sugarcane variety were not used as pre-stratification criteria, but were recorded as characteristics of the randomly selected commercial blocks during field assessment. This explains why the distribution of crop ages and varieties was not uniform across altitude classes. These revisions improve transparency of the sampling framework and facilitate interpretation of the results.

Line 106 – 109 Do the rainy seasons coincide with the infestation seasons? It would be better to explain the relationship between E. saccharina and the environment (rainfall), relating the insect's life stages, sugarcane growth, and the amount of precipitation in each location.

Response: The paragraph has been revised to clarify that the rainy and dry seasons used in the survey were defined according to the local rainfall patterns of each altitude classes and were not assumed a priori to coincide with peak infestation periods. The revised text now explains that precipitation influences sugarcane growth, crop moisture status, and plant stress, which in turn affect host suitability for E. saccharina oviposition, larval establishment, survival, and internal stalk damage. It also clarifies that rainfall amount and seasonal distribution differ among locations and locally defined rainy and dry periods were therefore used to compare infestation patterns across the three study zones.

Results

Reviewer 1 comments

Why variety is not included as a fixed factor in the model and likelihood ratio test table?

Response: The inclusion of sugarcane variety in the main generalized linear mixed model was initially explored alongside season, altitude, crop age, and their interactions. However, the variety variable was highly unbalanced across the study sites, with several varieties occurring only within a single altitude zone or represented by very few observations. As a result, including variety in the full ecological model produced rank-deficient design matrices, convergence problems, and unstable parameter estimates, which reduced the reliability of statistical inference. Therefore, variety was excluded to maintain model stability and interpretability. The influence of sugarcane variety was therefore assessed separately using a nested analytical framework, where varieties were compared within their respective altitude zones.

This approach provided a more appropriate ecological description of varietal differences while avoiding confounding between variety and altitude due to the unbalanced distribution of varieties across attitudes. The methods and results sections have been revised to clarify this analytical decision

Table 5. Why seasonal

Table 5. Why was the contrast column was included while it has the same information?

Response: The table was intended to present the seasonal contrast between dry and rain seasons within each altitude class. The caption has been revised to make this clearer, and the term “across crop ages” has been changed to “averaged over crop age” because crop age was included in the model but not displayed as a separate grouping factor in the table. The “contrast” column has also been removed because all rows represented the same comparison, dry season-rain season. The information is now provided in the table caption to avoid redundancy.

Reviewer 2 comments

Results section Restructuring -- Combine Results 3.1 and 3.3, as they address related infestation metrics.

Response: The infestation count and percentage stalk-bored results have been merged into a single subsection because both represent closely related measures of stalk-boring incidence. The revised section now reports the likelihood ratio test results once, followed by post-hoc and predicted-response outputs to explain the significant season X altitude X crop age interaction. The percentage stalk-bored results were used to show how the observed infestation pattern translated into visible stalk damage across altitude class. The seasonal contrast has also been clarified as dry season-rainy season, with negative estimates indicating higher predicted boring during the rainy season.

Provide full model output including random effect variances

Response: Full model output has now been added to the results section. In addition to the likelihood-ratio test table, the revised results now include the negative-binomial dispersion parameter, convergence information, Akaike Information Criterion (AIC), Bayesian Information Criterion, Random effect variance, and standard deviation.

Conduct sensitivity analysis to justify n=50

Response: A count-based sensitivity analysis has now been added to justify the 50-stalk sampling protocol. Because the main response variable was Eldana saccharina larval count per 50 stalks, the sensitivity analysis was conducted on the count scale using the fitted negative binomial distribution. The analysis compared detection probability and expected relative variability under the observe 50-stalk and a hypothetical 100-stalk sampling protocols. The results showed that 50-stalk sampling was less sensitive when expected infestation was very low, particularly below 1-2 individuals per 50 stalks. However, detection probability increased rapidly with expected count and 93% at 5 individuals per 50 stalks, 97.9% at 10 individuals per 50 stalks, and 99.5% at 20 individuals per 50 stalks. The manuscript has therefore been revised to clarify that the 50-stalk sampling protocol was adequate for detecting moderate to high infestation levels and for comparing field-level patterns among altitudes, seasons, and crop ages, while acknowledging possible under-detection of very low or highly localized infestation.

Validation data for damage assessment (e.g., correlation between red stalk length and larval counts

Response: The authors now included an internal validation analysis of the damage assessment. Spearman’s rank correlation was used to test the association between E. saccharina larval counts per 50 stalks and measured stalk damage indicators. The analysis indicated positive and significant correlations between larval counts and percentage stalk length red (ρs = 0.61, P < 0.001), total red stalk length (ρs = 0.6, P < 0.001), percentage stalks bored (ρs = 0.56, P < 0.001), and total number of bored internodes (ρs = 0.56, P < 0.001). These findings support the biological consistency of the damage assessment. The manuscript has been revised to include this analysis and clarify that damage indicators represent cumulative feeding injury, while larval counts represent infestation detected at the time of sampling.

Reviewer 3 comments

This manuscript provides a useful assessment of Eldana saccharina infestation across three zones in Tanzania. While the study is well-structured and offers informative data, but it lacks the broader context required for a global audience. The scope of the study is limited to a survey of infestation status; it does not quantify the actual economic losses caused by E. saccharina in the surveyed locations. The manuscript fails to connect these findings to practical management strategies or discuss how the current data can be utilized to improve pest control in the region. Apart from this, the manuscript would benefit significantly from the inclusion of high-quality images demonstrating the natural incidence of E. saccharina in the field.

Response: The manuscript has been revised to provide broader context on the importance of E. saccharina infestation in sugarcane production systems and clarify the relevance of the Tanzanian survey for pest surveillance and regional management planning. Additional text has been included in the discussion to explain how the observed variation across altitudes, seasons, and crop ages can guide practical monitoring, particularly by prioritizi

Attachment Submitted filename: Response to Reviewers.docx

# Peer review: Decision Letter 1

**DOI:** 10.1371/journal.pone.0355844.r003

**Authors:** Noé Aguilar-Rivera

28 Jul 2026

<p>Factors affecting the infestation status of African sugarcane stalk borer (*Eldana saccharina*  Walker) (Lepidoptera: Pyralidae) in Tanzania

PONE-D-26-23049R1

Dear Dr. Hamis Daniel Wambura

We’re pleased to inform you that your manuscript has been judged scientifically suitable for publication and will be formally accepted for publication once it meets all outstanding technical requirements.

Within one week, you’ll receive an e-mail detailing the required amendments. When these have been addressed, you’ll receive a formal acceptance letter and your manuscript will be scheduled for publication.

An invoice will be generated when your article is formally accepted. Please note, if your institution has a publishing partnership with PLOS and your article meets the relevant criteria, all or part of your publication costs will be covered. Please make sure your user information is up-to-date by logging into Editorial Manager at Editorial Manager® and clicking the ‘Update My Information' link at the top of the page. For questions related to billing, please contact billing support.

If your institution or institutions have a press office, please notify them about your upcoming paper to help maximize its impact. If they’ll be preparing press materials, please inform our press team as soon as possible -- no later than 48 hours after receiving the formal acceptance. Your manuscript will remain under strict press embargo until 2 pm Eastern Time on the date of publication. For more information, please contact onepress@plos.org.

Kind regards,

Noé Aguilar-Rivera

Academic Editor

PLOS One

# Editor report: Acceptance letter

**DOI:** 10.1371/journal.pone.0355844.r004

**Authors:** Noé Aguilar-Rivera

PONE-D-26-23049R1

PLOS One

Dear Dr. Wambura,

I'm pleased to inform you that your manuscript has been deemed suitable for publication in PLOS One. Congratulations! Your manuscript is now being handed over to our production team.

At this stage, our production department will prepare your paper for publication. This includes ensuring the following:

* All references, tables, and figures are properly cited

* All relevant supporting information is included in the manuscript submission,

* There are no issues that prevent the paper from being properly typeset

You will receive further instructions from the production team, including instructions on how to review your proof when it is ready. Please keep in mind that we are working through a large volume of accepted articles, so please give us a few days to review your paper and let you know the next and final steps.

Lastly, if your institution or institutions have a press office, please let them know about your upcoming paper now to help maximize its impact. If they'll be preparing press materials, please inform our press team within the next 48 hours. Your manuscript will remain under strict press embargo until 2 pm Eastern Time on the date of publication. For more information, please contact onepress@plos.org.

You will receive an invoice from PLOS for your publication fee after your manuscript has reached the completed accept phase. If you receive an email requesting payment before acceptance or for any other service, this may be a phishing scheme. Learn how to identify phishing emails and protect your accounts at https://explore.plos.org/phishing.

If we can help with anything else, please email us at customercare@plos.org.

Thank you for submitting your work to PLOS One and supporting open access.

Kind regards,

PLOS One Editorial Office Staff

on behalf of

Dr. Noé Aguilar-Rivera

Academic Editor

PLOS One
